# Supplementary material for: Development and Applications of Chimera Platforms for Tyrosine Phosphorylation
Source: ACS Cent Sci. 2023 Aug 9;9(8):1558–66. doi: 10.1021/acscentsci.3c00200 (PMC10450875; doi:10.1021/acscentsci.3c00200)
Supplement: Supplementary file 1 — oc3c00200_si_001.pdf [file oc3c00200_si_001.pdf]

Development and applications of chimera platforms for tyrosine phosphorylation

Rajaiah Pergu,[1,4] Veronika M. Shoba,[1,4] Santosh K. Chaudhary,[1,4] Dhanushka N. P. Munkanatta Godage,[1,4] Arghya Deb,[1,4] Santanu Singha,[1] Uttam Dhawa,[1] Prashant Singh,[1] Viktoriya Anokhina,[1] Sameek Singh,[1] Sachini U. Siriwardena,[1] and Amit Choudhary[1,2,3 *]

[1]Chemical Biology and Therapeutics Science, Broad Institute of MIT and Harvard, Cambridge, MA 02142, USA

[2]Department of Medicine, Harvard Medical School, Boston, MA 02115, USA

[3]Divisions of Renal Medicine and Engineering, Brigham and Women's Hospital, Boston, MA 02115, USA

[4]These authors contributed equally to this work

*To whom correspondence should be addressed:
Amit Choudhary
Chemical Biology and Therapeutics Science
Broad Institute of MIT and Harvard
415 Main Street, Rm 3012
Cambridge, MA 02142
Phone: (617) 714-7445
Fax: (617) 715-8969
Email: achoudhary@bwh.harvard.edu

Table of Contents

1. Compounds characterization. *S2*

1. 1. Synthesis details and characterization of compounds. S2

1. 2. ^{1}H and ^{13}C NMR spectra. S19

2. References. *S49*

1. Compounds characterization.

1. 1. Synthesis details and characterization of compounds.

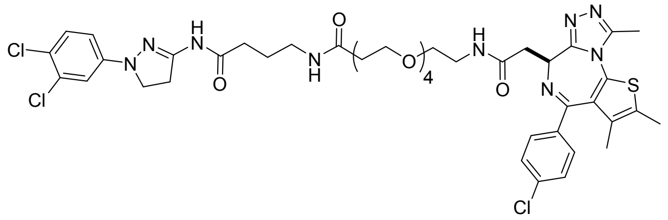

(*S*)-1-(2-(4-(4-chlorophenyl)-2,3,9-trimethyl-6H-thieno[3,2-f][1,2,4]triazolo[4,3-a][1,4]diazepin-6-yl)acetamido)-N-(4-((1-(3,4-dichlorophenyl)-4,5-dihydro-1H-pyrazol-3-yl)amino)-4-oxobutyl)-3,6,9,12-tetraoxapentadecan-15-amide (1). Step 1: 4-(tert-Butoxycarbonylamino)butyric acid (90 mg, 440 μmol), HATU (220 mg, 580 μmol), and DIPEA (220 μL, 1.26 mmol) were dissolved in dichloromethane (4.5 mL) and left to stir at room temperature for 30 min. After 30 min, 1-(3,4-Dichlorophenyl)-4,5-dihydro-1H-pyrazol-3-amine (**32**) (compound 28 in Simpson et al. [1], 90 mg, 393 μmol) was added, and the reaction mixture was stirred at room temperature overnight. The next day, the reaction mixture was quenched with sodium bicarbonate, and the product was extracted with dichloromethane, washed with brine, and concentrated under reduced pressure. The solid residue was purified via flash column chromatography (gradient Hexane:EtOAc from 100:0 to 70:30, two compounds came out from the column and the first one is the desired product), affording 82 mg (50% yield) of the desired product **33** as an off-white solid. ^{1}H NMR (400 MHz, DMSO-d6) δ 10.66 (s, 1H), 7.37 (d, *J* = 8.9 Hz, 1H), 7.01 (d, *J* = 2.7 Hz, 1H), 6.79 (dd, *J* = 8.9, 2.8 Hz, 2H), 3.68 (t, *J* = 9.8 Hz, 3H), 3.34 (t, *J* = 9.8 Hz, 2H), 2.91 (q, *J* = 6.6 Hz, 2H), 2.27 (t, *J* = 7.4 Hz, 2H), 1.69 – 1.57 (m, 2H), 1.36 ppm (s, 9H); ^{13}C NMR (101 MHz, DMSO) δ 171.58, 155.82, 150.73, 146.80, 131.49, 130.80, 118.52, 113.30, 112.46, 77.75, 48.01, 33.23, 32.80, 28.40, 25.29 ppm. Step 2: **33** (20.7 mg, 50 μmol) was dissolved in 2 mL of CH_2Cl_2, cooled to 0 °C and trifluoroacetic acid (0.5 mL) was added. The reaction mixture was allowed to warm up to room temperature and stirred for 30 minutes. before the solvent was concentrated under a reduced pressure. The solid residue was dissolved in 2 mL DMF and (*S*)-JQ1-PEG4-acid (33 mg, 50 μmol) was added followed by PyBOP (26 mg, 50 μmol), and DIPEA (30 μL, 172 μmol). The reaction mixture was stirred for 1h, concentrated under reduced pressure and purified by HPLC, affording 42 mg (89%) of the desired product **1**. ^{1}H NMR (400 MHz, MeOH-d4) δ 7.42 (q, *J* = 8.6 Hz, 4H), 7.23 (d, *J* = 8.9 Hz, 1H), 7.02 (d, *J* = 2.6 Hz, 1H), 6.74 (dd, *J* = 8.8, 2.6 Hz, 1H), 4.65 (dd, *J* = 9.1, 5.1 Hz, 1H), 3.72–3.58 (m, 19H), 3.49–3.37 (m, 5H), 3.35–3.28 (m, 4H), 3.23 (t, *J* = 6.8 Hz, 2H), 2.67 (s, 3H), 2.44–2.41 (m, 5H), 2.37 (t, *J* = 7.4 Hz, 2H), 1.80 (p, *J* = 7.0 Hz, 2H), 1.67 (s, 3H). ^{13}C NMR (101 MHz, MeOH-d4) δ 174.11, 173.66, 172.90, 166.12, 156.99, 152.11, 150.94, 148.27, 138.12, 137.94, 133.51, 133.38, 133.14, 132.01, 131.92, 131.45, 131.35, 129.79, 121.22, 114.95, 113.30, 71.59, 71.53, 71.46, 71.35, 70.64, 68.28, 55.15, 40.56, 39.56, 38.71, 37.76, 34.42, 33.77, 26.08, 14.45, 12.94, 11.64 ppm. HRMS (ESI-TOF): calculated for $C_{43}H_{53}Cl_3N_9O_7S$ (M+H): 944.2849, found: 944.2846.

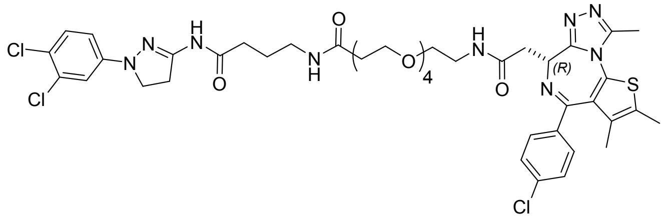

(*R*)-1-(2-(4-(4-chlorophenyl)-2,3,9-trimethyl-6H-thieno[3,2-f][1,2,4]triazolo[4,3-a][1,4]diazepin-6-yl)acetamido)-N-(4-((1-(3,4-dichlorophenyl)-4,5-dihydro-1H-pyrazol-3-yl)amino)-4-oxobutyl)-3,6,9,12-tetraoxapentadecan-15-amide (2). **33** (20.7 mg, 50 µmol), obtained following procedure for **1**, was dissolved in 2 mL of CH_2Cl_2, cooled to 0 °C and trifluoroacetic acid (0.5 mL) was added. The reaction mixture was allowed to warm up to room temperature and stirred for 30 minutes. before the solvent was concentrated under a reduced pressure. The solid residue was dissolved in 2 mL DMF and (*R*)-JQ1-PEG4-acid 33 mg, 50 µmol) was added followed by PyBOP (26 mg, 50 µmol), and DIPEA (30 µL, 172 µmol). The reaction mixture was stirred for 1h, concentrated under reduced pressure and purified by HPLC, affording 25 mg (53%) of the desired product **2**. ^{1}H NMR (400 MHz, MeOH-d4) δ 7.42 (q, *J* = 8.3 Hz, 4H), 7.23 (d, *J* = 8.9 Hz, 1H), 7.02 (d, *J* = 2.7 Hz, 1H), 6.74 (dd, *J* = 8.9, 2.7 Hz, 1H), 4.67–4.62 (m, 1H), 3.80 – 3.54 (m, 19H), 3.54 – 3.34 (m, 6H), 3.34 – 3.25 (m, 4H), 3.23 (t, *J* = 6.8 Hz, 2H), 2.67 (s, 3H), 2.44 – 2.37 (m, 7H), 1.81 (p, *J* = 7.2 Hz, 2H), 1.67 ppm (s, 3H). ^{13}C NMR (101 MHz, MeOH-d4) δ 174.12, 173.71, 172.90, 166.15, 156.99, 152.13, 150.94, 148.27, 138.11, 137.95, 133.51, 133.38, 133.16, 132.02, 131.93, 131.46, 131.35, 129.79, 121.24, 114.96, 113.32, 71.59, 71.53, 71.46, 71.34, 70.63, 68.28, 55.15, 40.57, 39.56, 38.71, 37.76, 34.42, 33.77, 26.08, 14.45, 12.95, 11.64. HRMS (ESI-TOF): calculated for $C_{43}H_{53}Cl_3N_9O_7S$ (M+H): 944.2849, found: 944.2851.

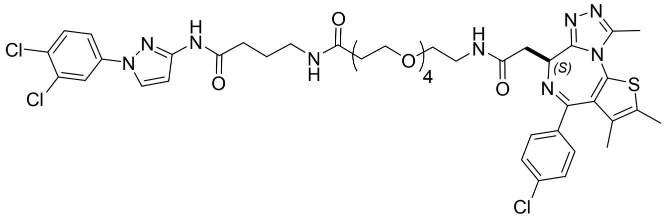

(*S*)-1-(2-(4-(4-chlorophenyl)-2,3,9-trimethyl-6H-thieno[3,2-f][1,2,4]triazolo[4,3-a][1,4]diazepin-6-yl)acetamido)-N-(4-((1-(3,4-dichlorophenyl)-1H-pyrazol-3-yl)amino)-4-oxobutyl)-3,6,9,12-tetraoxapentadecan-15-amide (3). Step 1. Butoxycarbonylamino)butyric acid (132 mg, 650 µmol) 1-(3,4-dichlorophenyl)-1H-pyrazol-3-amine **34** (140 mg, 620 µmol), HATU (240 mg, 630 µmol), and DIPEA (120 µL, 690 µmol) were dissolved in DMF (2 mL) and left to stir at room temperature overnight. The next day, the reaction mixture was quenched with sodium bicarbonate, and the product was extracted with dichloromethane, washed with brine, and concentrated under reduced pressure. The solid residue was purified via flash column chromatography (gradient Hex:EtOAc from 100:0 to 60:40), affording 90 mg (35% yield) of the desired product **35** as an off-white solid. ^{1}H NMR (400 MHz, MeOH-d4) δ 8.15 (d, *J* = 2.7 Hz, 1H), 7.94 (d, *J* = 2.5 Hz, 1H), 7.65 (dd, *J* = 8.8, 2.6 Hz, 1H), 7.55 (d, *J* = 8.8 Hz, 1H), 6.84 (d, *J* = 2.6 Hz, 1H), 3.12 (t, *J* = 6.9 Hz, 2H), 2.42 (t, *J* = 7.5 Hz, 2H), 1.85 (p, *J* = 7.2 Hz, 2H), 1.43 ppm (s, 9H). ^{13}C NMR (101 MHz, MeOH-d4) δ 173.58, 158.56, 150.71, 140.71, 134.17, 132.22, 129.97, 129.04, 120.83, 118.40, 102.02, 79.99, 40.87, 34.62, 28.76, 26.98. Step 2. **35**

(20.6 mg, 50 µmol) was dissolved in 2 mL of CH_2Cl_2, cooled to 0 °C and trifluoroacetic acid (0.5 mL) was added. The reaction mixture was allowed to warm up to room temperature and stirred for 30 minutes. before the solvent was concentrated under a reduced pressure. The solid residue was dissolved in 2 mL DMF and (*S*)-JQ1-PEG4-acid (33 mg, 50 µmol) was added followed by PyBOP (26 mg, 50 µmol), and DIPEA (30 µL, 172 µmol). The reaction mixture was stirred for 1h, concentrated under reduced pressure and purified by HPLC, affording 15 mg (32%) of the desired product **3**. ^{1}H NMR (400 MHz, MeOH-d4) δ δ 8.18 (d, *J* = 2.7 Hz, 1H), 7.94 (d, *J* = 2.5 Hz, 1H), 7.66 (dd, *J* = 8.8, 2.6 Hz, 1H), 7.55 (d, *J* = 8.8 Hz, 1H), 7.49 – 7.29 (m, 4H), 6.86 (d, *J* = 2.6 Hz, 1H), 4.68 (dd, *J* = 9.1, 5.1 Hz, 1H), 3.72 (t, *J* = 6.0 Hz, 2H), 3.67 – 3.59 (m, 13H), 3.54 – 3.37 (m, 3H), 3.32 – 3.18 (m, 2H), 2.69 (s, 3H), 2.53 – 2.33 (m, 7H), 1.88 (p, *J* = 7.1 Hz, 2H), 1.67 (s, 3H). ^{13}C NMR (101 MHz, MeOH-d4) δ 174.10, 173.37, 172.90, 166.09, 157.01, 152.08, 150.73, 140.69, 138.10, 137.93, 134.15, 133.49, 133.13, 132.25, 131.98, 131.94, 131.33, 129.91, 129.76, 129.08, 120.77, 118.38, 101.99, 71.58, 71.53, 71.46, 71.35, 71.33, 70.62, 68.27, 55.17, 40.56, 39.74, 38.74, 37.78, 34.53, 26.39, 14.41, 12.92, 11.61. ppm. HRMS (ESI-TOF): calculated for $C_{43}H_{51}Cl_3N_9O_7S$ (M+H): 942.2692, found: 942.2685.

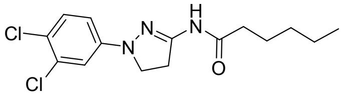

N-(1-(3,4-dichlorophenyl)-4,5-dihydro-1H-pyrazol-3-yl)hexanamide (4). Hexanoic acid (23 mg, 200 µmol) was dissolved in 2 mL DMF and treated with HATU (100 mg, 260 µmol), and DIPEA (100 µL, 570 µmol). The reaction mixture was stirred at room temperature for 30 min and 1-(3,4-Dichlorophenyl)-4,5-dihydro-1H-pyrazol-3-amine **32** (46 mg, 200 µmol) was then added, and the reaction mixture was stirred at room temperature overnight. The next day, the reaction mixture was quenched with sodium bicarbonate, and the product was extracted with dichloromethane, washed with brine, and concentrated under reduced pressure. The solid residue was purified via flash column chromatography (gradient Hex:EtOAc from 100:0 to 70:30) affording 15 mg (23% yield) of the desired product **4** as a yellowish solid.

^{1}H NMR (400 MHz, DMSO-d6) δ 10.69 (s, 1H), 7.40 (d, *J* = 8.9 Hz, 1H), 7.03 (d, *J* = 2.6 Hz, 1H), 6.81 (dd, *J* = 8.9, 2.7 Hz, 1H), 3.70 (t, *J* = 9.8 Hz, 2H), 2.29 (t, *J* = 7.5 Hz, 2H), 1.54 (p, *J* = 7.4 Hz, 2H), 1.36 – 1.22 (m, 4H), 0.87 ppm (t, *J* = 6.9 Hz, 3H). ^{13}C NMR (101 MHz, DMSO-d6) δ 171.73, 150.72, 146.69, 131.35, 130.68, 118.32, 113.15, 112.30, 47.87, 35.61, 32.69, 30.79, 24.44, 21.89, 13.87 ppm. HRMS (ESI-TOF): calculated for $C_{15}H_{20}Cl_2N_3O$ (M+H): 328.0978, found: 328.0978.

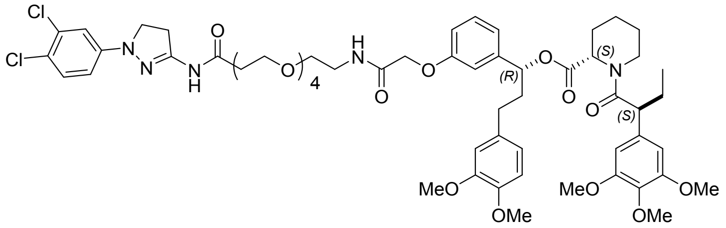

(*R*)-1-(3-((23-((1-(3,4-dichlorophenyl)-4,5-dihydro-1H-pyrazol-3-yl)amino)-2,18,23-trioxo-6,9,12,15-tetraoxa-3,19-diazatricosyl)oxy)phenyl)-3-(3,4-dimethoxyphenyl)propyl (*S*)-1-((*S*)-2-(3,4,5-

trimethoxyphenyl)butanoyl)piperidine-2-carboxylate (5). Step 1. AP1867 (35 mg, 50 µmol) and *tert*-butyl 1-amino-3,6,9,12-tetraoxapentadecan-15-oate (16 mg, 50 µmol), PyBOP (26 mg, 50 µmol), were dissolved in 2 mL DMF and treated with DIPEA (20 µL, 115 µmol). The reaction mixture was stirred for 1h, concentrated under reduced pressure and purified by HPLC affording 43 mg (86% yield) of intermediate **39**. Step 2. Intermediate **39** (43 mg, 43 µmol) was dissolved in 1 mL CH_2Cl_2 and treated with TFA (300 µL). Reaction mixture was stirred for 30 min, concentrated under reduced pressure, redissolved in CH_2Cl_2 (450 µL) and treated with HATU (22 mg, 58 µmol) followed by DIPEA (50 µL, 285 µmol). After stirring for 30 min at room temperature, 1-(3,4-Dichlorophenyl)-4,5-dihydro-1H-pyrazol-3-amine (**32**) (10 mg, 44 µmol) was added, and the reaction mixture was stirred at room temperature overnight. The next day, the reaction mixture was concentrated under reduced pressure and purified by HPLC affording 14 mg (28% yield) of the desired product **5** as an oil. ^{1}H NMR (400 MHz, MeOH-d4) δ 7.37-7.18 (m, 2H), 7.08 (d, J=4 Hz, 1H), 7.07 - 6.96 (m, 1H), 6.88-6.67 (m, 5H), 6.64-6.57 (m, 3H), 5.83 (t, J=8 Hz, 0.26H, minor diastereomer), 5.60 (dd, *J* = 8.3, 5.4 Hz, 1H), 5.40 (d, *J* = 5.3 Hz, 1H) 4.58-4.48 (m, 2H), 4.09 (d, *J* = 13.6 Hz, 1H), 3.88 (t, *J* = 7.2 Hz, 1H), 3.84 – 3.79 (m, 6H), 3.79 – 3.73 (m, 3H), 3.72-3.67 (m, 9H), 3.61 – 3.51 (m, 13H), 3.48-3.39 (m, 5H), 3.35 – 3.19 (m, 6H), 2.83-2.71 (m, 1H), 2.64-2.52 (m, 3H), 2.48 - 2.40 (m, 1H), 2.32-2.28 (m, 1H), 2.10 – 1.96 (m, 2H), 1.88-1.76 (m, 1H), 1.61-1.52 (m, 4H), 1.48 – 1.24 (m, 3H), 0.91 (t, *J* = 8 Hz, 2.5H, major diastereomer), 0.84 (t, *J* = 8.0 Hz, 0.5H, minor diastereomer). ^{13}C NMR (101 MHz, MeOH-d4) δ 174.98, 172.28, 171.88, 171.05, 159.11, 154.88, 154.58, 150.88, 150.43, 148.87, 148.30, 143.62, 137.96, 136.92, 135.14, 133.41, 131.48, 130.92, 121.79, 121.34, 120.67, 115.14, 115.05, 114.46, 113.67, 113.38, 113.27, 106.60, 106.02, 77.14, 71.59, 71.55, 71.48, 71.45, 71.30, 70.41, 68.31, 67.87, 61.10, 57.39, 56.76, 56.57, 56.48, 53.62, 44.98, 40.04, 39.22, 38.05, 33.77, 32.26, 30.66, 29.38, 27.63, 26.32, 21.87, 12.99, 12.67 ppm. HRMS (ESI-TOF): calculated for $C_{58}H_{76}Cl_2N_5O_{15}$ (M+H): 1152.4709, found: 1152.4720.

General Procedure A: Preparation of C-N coupling with aryl pinacolboronates

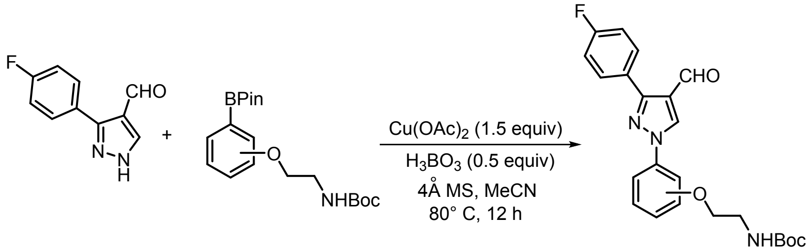

In an oven-dried 50 mL flask with a stir-bar, $Cu(OAc)_2$ (2 mmol, 1 equiv.), 3-(4-fluorophenyl)-1H-pyrazole-4-carbaldehyde **23** (2 mmol, 1 equiv), *tert*-butyl (2-methoxyethyl)carbamate substituted 4,4,5,5-tetramethyl-2-phenyl-1,3,2-dioxaborolane (3 mmol, 1.5 equiv) and H_3BO_3 (0.5 equiv) was added . To this mixture, 200 mg of 4 Å molecular sieves and dry acetonitrile (10 mL) was added at rt. The reaction mixture was stirred at 80 °C for 12 h. The reaction mixture was first evaporated in reduced pressure and then diluted with ethyl acetate (60 mL), washed with saturated NH_4Cl (10 mL x3), followed by Brine (10 mL), dried over anhydrous Na_2SO_4, filtered, and concentrated under reduced pressure. The crude residue was purified by Combiflash ISCO to obtain the pure product.

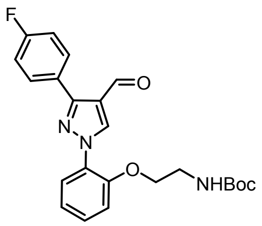

***tert*-butyl (2-(2-(3-(4-fluorophenyl)-4-formyl-1H-pyrazol-1-yl)phenoxy)ethyl)carbamate (25a)** was prepared by following the general procedure **A** using tert-butyl (2-(2-(4,4,5,5-tetramethyl-1,3,2-dioxaborolan-2-yl)phenoxy)ethyl)carbamate (3 mmol). Final product was isolated by flash column chromatography (Hexane: EtOAC 80:20) in 51% yield (433 mg; white solid). ^{1}H NMR (400 MHz, $CDCl_3$) δ 10.02 (s, 1H), 8.65 (s, 1H), 7.94 – 7.87 (m, 2H), 7.82 (dd, J = 8.0, 1.9 Hz, 1H), 7.36 (ddd, J = 8.0, 4.7, 2.0 Hz, 1H), 7.20 – 7.05 (m, 4H), 4.17 (q, J = 4.9 Hz, 2H), 3.58 – 3.49 (m, 2H), 1.40 (s, 9H). ^{13}C NMR (101 MHz, $CDCl_3$) δ 184.71, 163.62 (d, J = 249.5 Hz), 156.07, 152.49, 150.68, 138.02, 131.02 (d, J = 8.1 Hz), 129.62, 128.80, 127.81 (d, J = 3 Hz), 125.59, 121.92 (d, J = 9.1 Hz), 121.88, 115.78 (d, J = 22.2 Hz), 113.94, 79.97, 68.78, 40.22, 28.54.

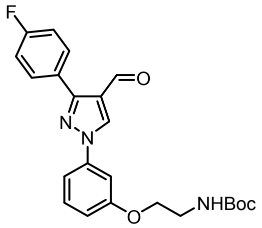

***tert*-butyl (2-(3-(3-(4-fluorophenyl)-4-formyl-1H-pyrazol-1-yl)phenoxy)ethyl)carbamate (25b)** was prepared by following the general procedure **A** using *tert*-butyl (2-(3-(4,4,5,5-tetramethyl-1,3,2-dioxaborolan-2-yl)phenoxy)ethyl)carbamate (3 mmol). Final product was isolated by flash column chromatography (Hexane: EtOAC 80:20) in 56% yield (476 mg; white solid). ^{1}H NMR (400 MHz, $CDCl_3$) δ 9.96 (s, 1H), 8.48 (s, 1H), 7.88 – 7.78 (m, 2H), 7.40 – 7.32 (m, 2H), 7.28 (dt, *J* = 7.6, 1.1 Hz, 1H), 7.17 – 7.09 (m, 2H), 6.86 (dt, *J* = 8.1, 1.6 Hz, 1H), 4.06 (t, *J* = 5.3 Hz, 2H), 3.52 (q, *J* = 5.5 Hz, 2H), 1.42 (s, 11H). ^{13}C NMR (101 MHz, $CDCl_3$) δ ^{13}C NMR (101 MHz, $CDCl_3$) δ 184.58, 163.64 (d, J = 249.5 Hz), 162.41, 159.78, 156.06, 153.38, 140.15, 132.32, 130.96 (d, J = 8.1 Hz), 130.71, 127.62 (d, J = 3 Hz), 122.58, 115.96 (d, J = 22.2 Hz), 114.16, 111.93, 106.56, 79.82, 67.68, 40.12, 28.55.

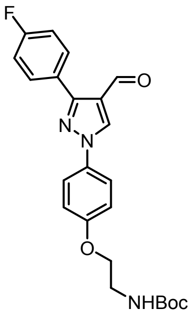

***tert*-butyl (2-(4-(3-(4-fluorophenyl)-4-formyl-1H-pyrazol-1-yl)phenoxy)ethyl)carbamate (25c)** was prepared by following the general procedure **A** using *tert*-butyl (2-(4-(4,4,5,5-tetramethyl-1,3,2-dioxaborolan-2-

yl)phenoxy)ethyl)carbamate (3 mmol). Final product was isolated by flash column chromatography (Hexane: EtOAC 80:20) in 61% yield (510 mg; white solid). ^{1}H NMR (400 MHz, $CDCl_3$) δ 9.99 (s, 1H), 8.41 (s, 1H), 7.83 (dd, J = 8.7, 5.4 Hz, 2H), 7.72 – 7.59 (m, 2H), 7.16 (t, J = 8.7 Hz, 2H), 7.06 – 6.95 (m, 2H), 4.06 (t, J = 5.2 Hz, 2H), 3.55 (d, J = 5.5 Hz, 2H), 1.45 (s, 9H). ^{13}C NMR (101 MHz, $CDCl_3$) δ δ 184.69, 163.61 (d, J= 250.5 Hz), 158.51, 156.05, 153.41, 132.93, 131.81, 130.94 (d, J=8.1 Hz), 127.74 (d, J=3.0 Hz), 122.35, 121.48, 115.87 (d, J=21.2 Hz), 115.48, 79.84, 67.78, 40.23, 28.57.

General Procedure B: Bucherer Berg reaction

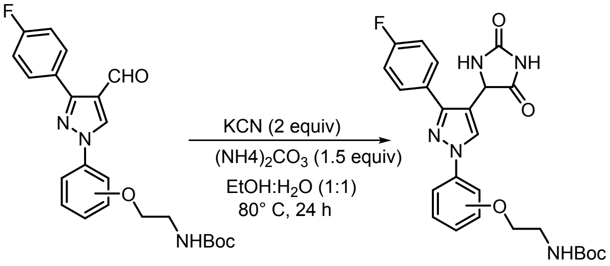

25 **26**

In an oven-dried 50 mL sealed-tube with a stir-bar, starting aldehyde (1 mmol), $(NH_4)_2CO_3$ (1.5 mmol, 1.5 equiv.) and KCN (2 mmol, 2 equiv) was added. To this mixture, 5 mL of ethanol (EtOH) and distilled wated (5 mL) was added at rt. The tube was sealed with a teflon cap and the reaction mixture was stirred at 80 °C for 24 h. The reaction mixture was first evaporated in reduced pressure and then diluted with ethyl acetate (60 mL), washed with saturated NH_4Cl (10 mL x3), followed by Brine (10 mL), dried over anhydrous Na_2SO_4, filtered, and concentrated under reduced pressure. The crude residue was purified by Combiflash ISCO to obtain the pure product.

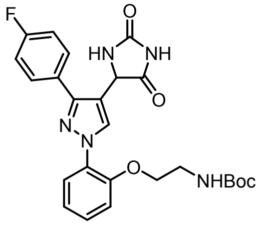

tert-butyl (2-(2-(4-(2,5-dioxoimidazolidin-4-yl)-3-(4-fluorophenyl)-1H-pyrazol-1-yl)phenoxy)ethyl)carbamate (26a) was prepared by following the general procedure **B** using tert-butyl (2-(2-(4,4,5,5-tetramethyl-1,3,2-dioxaborolan-2-yl)phenoxy)ethyl)carbamate as starting aldehyde. Final product was isolated by flash column chromatography (CH_2Cl_2: MeOH 97.5:2.5) in 49% yield (243 mg; yellow solid). ^{1}H NMR (400 MHz, MeOH-d4) δ 8.24 (s, 1H), 7.81 – 7.72 (m, 2H), 7.65 (dd, J = 7.9, 1.6 Hz, 1H), 7.40 – 7.33 (m, 1H), 7.20 (td, J = 8.9, 4.6 Hz, 3H), 7.10 (t, J = 7.7 Hz, 1H), 5.34 (s, 1H), 4.19 – 4.06 (m, 2H), 3.53 (dt, J = 11.7, 5.3 Hz, 1H), 3.44 – 3.35 (m, 1H), 1.37 (s, 9H). 13C NMR (101 MHz, MeOH-d4) δ 176.82, 164.62 (d, J= 247.4 Hz), 159.64, 158.51, 152.51, 152.22, 134.54, 132.04 (d, J= 9.1 Hz), 130.51, 130.37, 130.07(d, J= 3.0 Hz), 126.60, 122.55, 116.55 (d, J= 22.2 Hz), 115.99, 114.92, 80.41, 69.31, 55.79, 41.00, 28.87.

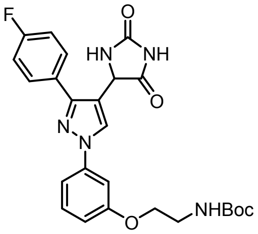

tert-butyl (2-(3-(4-(2,5-dioxoimidazolidin-4-yl)-3-(4-fluorophenyl) -1H-pyrazol-1-yl)phenoxy)ethyl)carbamate (26b) was prepared by following the general procedure **B** using *tert*-butyl (2-(3-(3-(4-fluorophenyl)-4-formyl-1H-pyrazol-1-yl) phenoxy) ethyl) carbamate as starting aldehyde. Final product was isolated by flash column chromatography (CH_2Cl_2: MeOH 97.5:2.5) in 52% yield (257 mg; yellow solid). ^{1}H NMR (400 MHz, MeOH-d4) δ 8.38 (s, 1H), 7.80 (ddd, *J* = 8.6, 4.3, 1.5 Hz, 2H), 7.45 – 7.30 (m, 3H), 7.20 (td, *J* = 8.7, 1.6 Hz, 2H), 6.91 (ddd, *J* = 6.2, 3.6, 2.4 Hz, 1H), 5.32 (s, 1H), 4.09 – 4.02 (m, 2H), 3.45 (tt, *J* = 5.8, 2.9 Hz, 2H), 1.44 (s, 9H). ^{13}C NMR (101 MHz, MeO-d4) δ 176.62, 164.66 (d, J= 247.5 Hz), 161.38, 159.69, 158.68, 153.05, 142.19, 131.98 (d, J= 8.1 Hz), 131.67, 130.05 (d, J= 3.0 Hz), 129.70, 117.64, 116.57(d, J= 22.2 Hz), 114.55, 112.61, 106.84, 80.40, 68.43, 55.67, 41.06, 28.89.

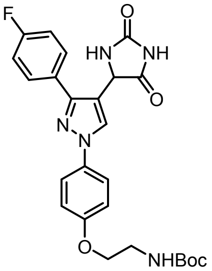

tert-butyl(2-(4-(4-(2,5-dioxoimidazolidin-4-yl)-3-(4-fluorophenyl)-1H-pyrazol-1-yl)phenoxy)ethyl)carbamate (26c) was prepared by following the general procedure **B** using *tert*-butyl (2-(4-(3-(4-fluorophenyl)-4-formyl-1H-pyrazol-1-yl)phenoxy)ethyl)carbamate as starting aldehyde. Final product was isolated by flash column chromatography (CH_2Cl_2: MeOH 97.5:2.5) in 56% yield (277 mg; yellow solid). ^{1}H NMR (400 MHz, MeO-d4) δ 8.25 (s, 1H), 7.78 (dd, *J* = 8.6, 5.5 Hz, 2H), 7.68 (d, *J* = 9.0 Hz, 2H), 7.25 – 7.15 (m, 2H), 7.10 – 7.02 (m, 2H), 5.32 (s, 1H), 4.04 (t, *J* = 5.6 Hz, 2H), 3.46 – 3.39 (m, 2H), 1.45 (s, 9H). ^{13}C NMR (101 MHz, MeO-d4) δ 176.57, 164.46 (d, J= 247.4 Hz), 159.55, 159.39, 158.54, 152.55, 134.74, 131.81 (d, J= 8.1 Hz), 129.99 (d, J= 3.0 Hz), 129.44, 122.08, 117.04, 116.4 (d, J= 22.2 Hz), 116.33, 80.24, 68.33, 55.54, 40.95, 28.74.

General Procedure C: Preparation of Acetylated DPH

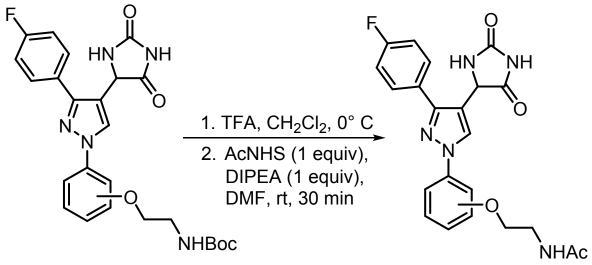

In an oven-dried 20 mL flask with a stir-bar, Boc protected DPH (0.25 mmol) was dissolved in dry dichloromethane and to the mixture 1.5 equivalent of trifluoroacetic acid (TFA) was added at 0 °C. The reaction kept stirring for 30 minutes and was monitored by LCMS. Upon complete deprotection, the solvent was evaporated by under reduced pressure and to remove excess TFA, the reaction mixture was evaporated with additional 10 mL of Toluene. To the same flask, 1 equivalent of AcNHS, 1 equivalent of DIPEA was added in 3 mL DMF. The reaction mixture was stirred at room temparature for 30 min-1hour. The reaction mixture was first evaporated in reduced pressure and then diluted with ethyl acetate (30 mL), washed with saturated NH_4Cl (10 mL x3), followed by Brine (10 mL), dried over anhydrous Na_2SO_4, filtered, and concentrated under reduced pressure. The crude residue was purified by Combiflash ISCO to obtain the pure product.

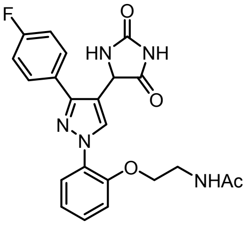

N-(2-(2-(4-(2,5-dioxoimidazolidin-4-yl)-3-(4-fluorophenyl)-1H-pyrazol-1-yl)phenoxy)ethyl)acetamide (6) was prepared by following the general procedure **C** using tert-butyl (2-(2-(4-(2,5-dioxoimidazolidin-4-yl)-3-(4-fluorophenyl)-1H-pyrazol-1-yl)phenoxy)ethyl)carbamate as starting material. Final product was isolated by flash column chromatography (CH_2Cl_2: MeOH 97.5:2.5) in 79% yield (86 mg; white solid). ^{1}H NMR (400 MHz, MeO-d4) δ 8.20 (s, 1H), 7.82 – 7.69 (m, 2H), 7.66 (dd, *J* = 8.0, 1.6 Hz, 1H), 7.38 (td, *J* = 7.9, 1.6 Hz, 1H), 7.20 (td, *J* = 8.5, 5.1 Hz, 3H), 7.10 (td, *J* = 7.7, 1.1 Hz, 1H), 5.35 (s, 1H), 4.17 (td, *J* = 5.3, 4.5, 1.7 Hz, 2H), 3.72 – 3.45 (m, 2H), 1.90 (s, 3H). ^{13}C NMR (101 MHz, MeOH-d4) δ 176.72, 173.65, 164.50 (d, J= 247.4 Hz), 159.46, 152.39, 152.12, 134.35, 131.88 (d, J= 8.1 Hz), 130.46, 130.28, 129.95 (d, J= 3.0 Hz), 126.57, 122.50, 116.41 (d, J= 21.2 Hz), 115.93, 114.82, 68.69, 55.61, 39.99, 22.59. HRMS (ESI-TOF): calculated for $C_{22}H_{21}FN_5O_4$ (M+H): 438.1572, found: 438.1574.

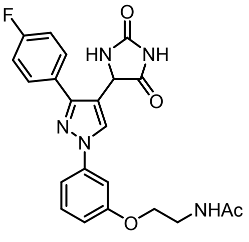

N-(2-(3-(4-(2,5-dioxoimidazolidin-4-yl)-3-(4-fluorophenyl)-1H-pyrazol-1-yl)phenoxy)ethyl)acetamide (8) was prepared by following the general procedure **C** using tert-butyl (2-(3-(4-(2,5-dioxoimidazolidin-4-yl)-3-(4-fluorophenyl) -1H-pyrazol-1-yl)phenoxy)ethyl)carbamate as starting material. Final product was isolated by flash column chromatography (CH_2Cl_2: MeOH 97.5:2.5) in 76% yield (83 mg; white solid). ^{1}H NMR (400 MHz, MeOH-d4) δ 8.40 (s, 1H), 7.84 – 7.74 (m, 2H), 7.41 (ddd, *J* = 19.0, 3.4, 1.5 Hz, 3H), 7.26 – 7.16 (m, 2H), 6.99 – 6.87 (m, 1H), 5.33 (s, 1H), 4.12 (t, *J* = 5.4 Hz, 2H), 3.58 (t, *J* = 5.4 Hz, 2H), 1.97 (s, 3H). ^{13}C NMR (101 MHz, MeOH-d4) δ 176.50, 173.65, 163.54 (d, J= 247.4 Hz), 161.19, 159.56, 152.94, 142.08, 131.85 (d, J= 8.1 Hz), 131.57, 129.92 (d, J= 3.0 Hz), 129.91, 129.57, 117.56, 116.42 (d, J= 21.2 Hz), 114.35, 112.49, 106.67, 67.84, 55.52, 40.11, 22.48. HRMS (ESI-TOF): calculated for $C_{22}H_{21}F_1N_5O_4$ (M+H): 438.1572, found: 438.1573.

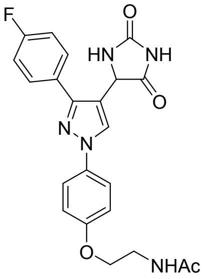

N-(2-(4-(4-(2,5-dioxoimidazolidin-4-yl)-3-(4-fluorophenyl)-1H-pyrazol-1-yl)phenoxy)ethyl)acetamide (10) was prepared by following the general procedure **C** using tert-butyl(2-(4-(4-(2,5-dioxoimidazolidin-4-yl)-3-(4-fluorophenyl)-1H-pyrazol-1-yl)phenoxy)ethyl)carbamate as starting material. Final product was isolated by flash column chromatography (CH_2Cl_2: MeOH 97.5:2.5) in 82% yield (89 mg; white solid). ^{1}H NMR (400 MHz, MeOH-d4) δ 8.25 (s, 1H), 7.82 – 7.73 (m, 2H), 7.72 – 7.66 (m, 2H), 7.19 (t, *J* = 8.8 Hz, 2H), 7.12 – 7.00 (m, 2H), 5.32 (s, 1H), 4.09 (t, *J* = 5.5 Hz, 2H), 3.58 (t, *J* = 5.5 Hz, 2H), 1.97 (s, 3H). ^{13}C NMR (101 MHz, DMSO) δ 174.80, 169.58, 162.24 (d, J= 245.4 Hz), 157.14(d, J= 3.0 Hz), 157.03, 150.04, 133.00, 130.19 (d, J= 9.1 Hz), 128.87 (d, J= 3.0 Hz), 128.11, 119.90, 116.32, 116.27, 115.56 (d, J= 21.2 Hz), 115.22, 66.73, 53.53, 38.29, 22.59. HRMS (ESI-TOF): calculated for $C_{22}H_{21}F_1N_5O_4$ (M+H): 438.1572, found: 438.1574.

General Procedure D: Preparation of Halotag Containing DPH

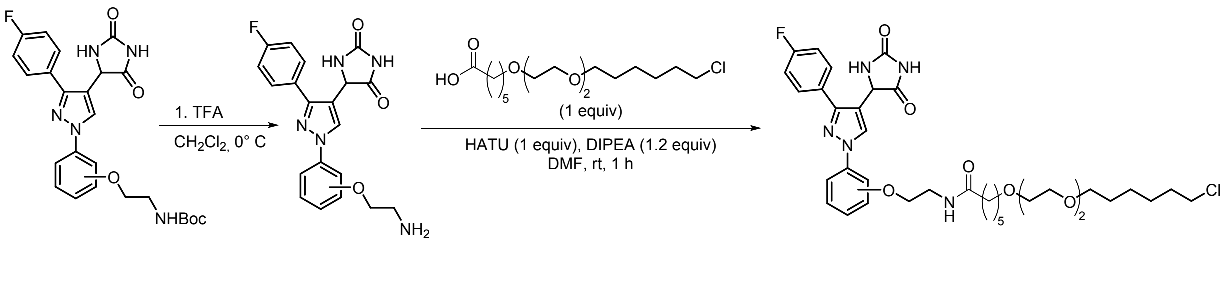

In an oven-dried 5 mL flask with a stir-bar, Boc protected DPH (0.1 mmol) was dissolved in dry dichloromethane and to the mixture 1.5 equivalent of trifluoroacetic acid (TFA) was added at 0 °C. The reaction kept stirring for

30 minutes and was monitored by LCMS. Upon complete deprotection, the solvent was evaporated by under reduced pressure and to remove excess TFA, the reaction mixture was evaporated with additional 10 mL of Toluene. To the same flask, 6-(2-(2-((6-chlorohexyl)oxy)ethoxy)ethoxy)hexanoic acid (0.1 mmol), 1 equivalent of HATU, 1.2 equivalent of DIPEA was added in 2 mL DMF. The reaction mixture was stirred at room temparature for 30 min-1hour. The reaction mixture was first evaporated in reduced pressure and then diluted with ethyl acetate (15 mL), washed with saturated NH_4Cl (5 mL x3), followed by Brine (5 mL), dried over anhydrous Na_2SO_4, filtered, and concentrated under reduced pressure. The crude residue was purified by Combiflash ISCO to obtain the pure product. Then further purified with prep-HPLC in water (0.1% formic acid) and acetonitrile (0.1% formic acid) as eluent.

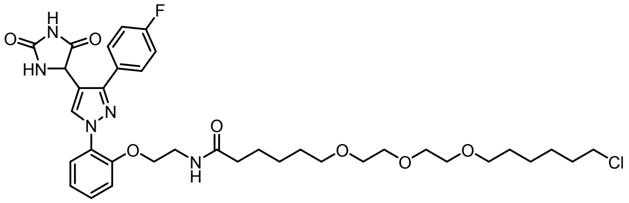

6-(2-(2-((6-chlorohexyl)oxy)ethoxy)ethoxy)-N-(2-(2-(4-(2,5-dioxoimidazolidin-4-yl)-3-(4-fluoroph-enyl)-1H-pyrazol-1-yl)phenoxy)ethyl)hexanamide (7) was prepared by following the general procedure **D** using tert-butyl (2-(2-(4-(2,5-dioxoimidazolidin-4-yl)-3-(4-fluorophenyl)-1H-pyrazol-1-yl)phenoxy)ethyl)carbamate as starting material. Final product was obtained in 42% yield (30 mg, yellow sticky liquid). ^{1}H NMR (400 MHz, $CDCl_3$) δ 9.40 (d, J = 1.6 Hz, 1H), 8.05 (s, 1H), 7.85 – 7.74 (m, 2H), 7.68 (dd, J = 7.9, 1.7 Hz, 1H), 7.64 (s, 1H), 7.31 – 7.23 (m, 1H), 7.17 – 7.07 (m, 2H), 7.06 – 7.00 (m, 1H), 6.95 (dd, J = 8.3, 1.2 Hz, 1H), 6.44 (t, J = 5.9 Hz, 1H), 5.27 (s, 1H), 4.09 (ddd, J = 8.9, 5.2, 3.3 Hz, 1H), 4.00 (td, J = 9.5, 8.8, 2.8 Hz, 1H), 3.82 (dp, J = 11.6, 4.3 Hz, 1H), 3.60 – 3.57 (m, 3H), 3.53 (d, J = 2.4 Hz, 1H), 3.50 – 3.44 (m, 4H), 3.40 (t, J = 6.7 Hz, 2H), 3.29 (td, J = 6.5, 1.8 Hz, 2H), 2.11 – 1.91 (m, 2H), 1.80 – 1.66 (m, 2H), 1.54 (p, J = 6.9 Hz, 2H), 1.40 (dq, J = 8.7, 6.6 Hz, 6H), 1.34 – 1.22 (m, 4H), 1.17 (q, J = 7.5 Hz, 2H), 0.94 – 0.77 (m, 1H). ^{13}C NMR (101 MHz, $CDCl_3$) δ 174.21, 163.15 (d, J = 248.5 Hz), 157.51, 150.59, 150.50, 132.12, 130.37 (d, J = 8.1 Hz), 129.29, 128.82, 128.57 (d, J = 3.0 Hz), 125.37, 121.75, 115.87 (d, J = 22.2 Hz) ,113.73, 113.26, 71.36, 71.21, 70.60, 70.58, 70.17, 70.01, 68.19, 54.77, 45.23, 38.86, 36.27, 32.66, 29.51, 29.02, 26.82, 25.59, 25.52, 25.43. ^{19}F NMR (376 MHz, $CDCl_3$) δ -113.10. HRMS (ESI-TOF): calculated for $C_{36}H_{48}Cl_1F_1N_5O_7$ (M+H): 716.3221, found: 716.3219.

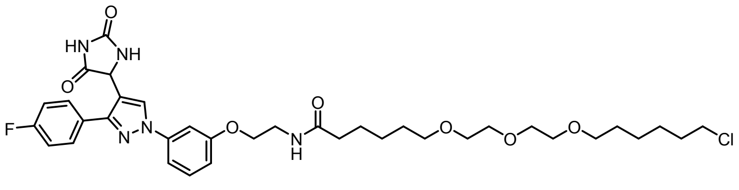

6-(2-(2-((6-chlorohexyl)oxy)ethoxy)ethoxy)-N-(2-(3-(4-(2,5-dioxoimidazolidin-4-yl)-3-(4-fluorophenyl)-1H-pyrazol-1-yl)phenoxy)ethyl)hexanamide (9) was prepared by following the general procedure **D** using tert-butyl (2-(3-(4-(2,5-dioxoimidazolidin-4-yl)-3-(4-fluorophenyl) -1H-pyrazol-1-yl)phenoxy)ethyl)carbamate as starting material. Final product was obtained in 41% yield (29 mg, yellow sticky liquid). ^{1}H NMR (400 MHz, $CDCl_3$)

δ 9.77 (s, 1H), 8.10 (s, 1H), 7.73 (dd, *J* = 8.4, 5.3 Hz, 2H), 7.22 (t, *J* = 5.6 Hz, 3H), 7.15 (s, 1H), 7.04 (t, *J* = 8.4 Hz, 2H), 6.71 (d, *J* = 6.8 Hz, 1H), 6.39 (s, 1H), 5.19 (d, *J* = 26.4 Hz, 1H), 4.32 – 4.08 (m, 1H), 3.98 (s, 2H), 3.55 (dd, *J* = 6.0, 3.2 Hz, 3H), 3.52 – 3.46 (m, 4H), 3.45 (m, 1H), 3.37 (t, *J* = 6.7 Hz, 2H), 3.31 (t, *J* = 6.6 Hz, 2H), 2.10 (t, *J* = 7.3 Hz, 2H), 2.03 (d, *J* = 12.2 Hz, 1H), 1.69 (p, *J* = 6.8 Hz, 2H), 1.49 (m, 5H), 1.36 (dd, *J* = 9.9, 6.2 Hz, 2H), 1.30 – 1.24 (m, 5H), 0.85 (t, *J* = 6.7 Hz, 2H). ^{13}C NMR (101 MHz, $CDCl_3$) δ 174.04, 163.13 (d, *J* = 249.5 Hz), 159.47, 157.78, 151.44, 140.64, 130.41 (d, *J* = 9.1 Hz), 128.23 (d, *J* = 3.0 Hz), 127.37, 115.77 (d, *J* = 21.2 Hz), 115.59, 113.07, 111.48, 105.43, 71.28, 71.18, 70.58, 70.54, 70.08, 70.02, 62.41, 54.50, 45.18, 36.47, 34.22, 32.61, 31.85, 29.80, 29.45, 29.26, 26.76, 25.74, 25.46. ^{19}F NMR (376 MHz, $CDCl_3$) δ -112.74. HRMS (ESI-TOF): calculated for $C_{36}H_{48}Cl_1F_1N_5O_7$ (M+H): 716.3221, found: 716.3216.

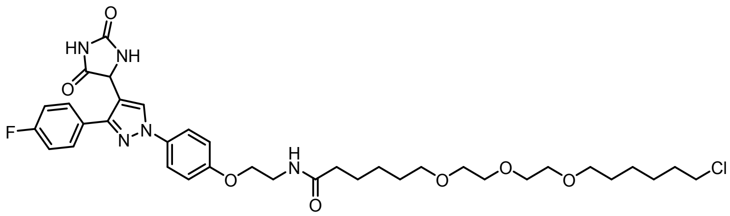

6-(2-(2-((6-chlorohexyl)oxy)ethoxy)ethoxy)-N-(2-(4-(4-(2,5-dioxoimidazolidin-4-yl)-3-(4-fluoroph --enyl)-1H-pyrazol-1-yl)phenoxy)ethyl)hexanamide (11) was prepared by following the general procedure **D** using tert-butyl(2-(4-(4-(2,5-dioxoimidazolidin-4-yl)-3-(4-fluorophenyl)-1H-pyrazol-1-yl)phenoxy)ethyl)carbamate as starting material. Final product was obtained in 48% yield (34 mg, yellow sticky liquid). ^{1}H NMR (400 MHz, $CDCl_3$) δ 9.58 (s, 1H), 7.92 (s, 1H), 7.72 (dd, *J* = 8.5, 5.3 Hz, 2H), 7.50 (d, *J* = 8.4 Hz, 2H), 7.06 (t, *J* = 8.5 Hz, 2H), 6.93 (s, 1H), 6.83 (d, *J* = 8.4 Hz, 2H), 6.31 (s, 1H), 5.18 (s, 1H), 4.41 – 4.05 (m, 1H), 3.93 (s, 2H), 3.58 (dd, *J* = 5.8, 3.0 Hz, 3H), 3.51 (ddd, *J* = 9.7, 5.1, 2.6 Hz, 4H), 3.47 (d, *J* = 6.6 Hz, 1H), 3.37 (dt, *J* = 16.4, 6.7 Hz, 4H), 2.14 (t, *J* = 7.5 Hz, 2H), 2.09 – 2.04 (m, 1H), 1.80 – 1.64 (m, 2H), 1.54 (dq, *J* = 21.7, 7.3 Hz, 5H), 1.45 – 1.34 (m, 2H), 1.33 – 1.23 (m, 5H), 0.84 (dt, *J* = 13.1, 6.9 Hz, 2H). ^{13}C NMR (101 MHz, $CDCl_3$) δ 173.94, 163.12 (d, *J* = 248.5 Hz), 157.66, 157.53, 151.18, 133.55, 130.39 (d, *J* = 8.1 Hz), 128.39 (d, *J* = 4.0 Hz), 127.05, 120.71, 115.94, 115.83 (d, *J* = 21.2 Hz), 115.05, 71.33, 71.24, 70.62, 70.13, 67.18, 54.56, 45.23, 39.05, 36.54, 34.25, 32.65, 29.84, 29.49, 29.34, 26.80, 25.82, 25.57, 25.50. ^{19}F NMR (376 MHz, $CDCl_3$) δ -112.81. HRMS (ESI-TOF): calculated for $C_{36}H_{48}Cl_1F_1N_5O_7$ (M+H): 716.3221, found: 716.3223.

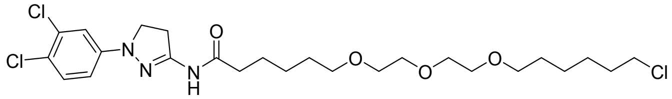

6-(2-(2-((6-chlorohexyl)oxy)ethoxy)ethoxy)-N-(1-(3,4-dichlorophenyl)-4,5-dihydro-1H-pyrazol-3-yl)hexanamide (12). 6-(2-(2-((6-chlorohexyl)oxy)ethoxy)ethoxy)hexanoic acid (17 mg, 50 µmol), HATU (32.2 mg, 85 µmol), and DIPEA (30 µL, 173 µmol) were dissolved in DMF (1 mL) and were stirred at room temperature for 30 min. 1-(3,4-Dichlorophenyl)-4,5-dihydro-1H-pyrazol-3-amine **32** (20 mg, 87 µmol) was then added, and the reaction mixture was stirred at room temperature overnight. The next day reaction mixture was concentrated under reduced pressure and purified by HPLC affording the desired product **15** as an oil. ^{1}H NMR (400 MHz,

MeOH-d4) δ 7.27 (d, *J* = 8.9 Hz, 1H), 7.08 (d, *J* = 2.7 Hz, 1H), 6.80 (dd, *J* = 8.9, 2.7 Hz, 1H), 3.72 (t, *J* = 9.9 Hz, 2H), 3.66 – 3.52 (m, 10H), 3.52 – 3.35 (m, 6H), 2.35 (t, *J* = 7.4 Hz, 2H), 1.82 – 1.51 (m, 8H), 1.51 – 1.24 (m, 6H). ^{13}C NMR (101 MHz, MeOH-d4) δ 174.50, 150.94, 148.35, 133.41, 131.46, 121.32, 115.04, 113.36, 72.17, 72.06, 71.59, 71.20, 71.18, 45.70, 37.28, 33.77, 30.56, 30.40, 27.74, 26.80, 26.50, 26.29 ppm. HRMS (ESI-TOF): calculated for $C_{25}H_{39}Cl_3N_3O_4$ (M+H): 550.2001, found: 550.2007.

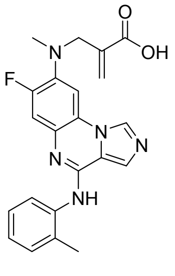

2-(((7-fluoro-4-(o-tolylamino)imidazo[1,5-a]quinoxalin-8-yl)(methyl)amino)methyl)acrylic acid (**41**). To a solution of **40** [2] (165.5 mg, 0.5 mmol) and 2-(bromomethyl)acrylic acid (82.5 mg, 0.5 mmol) in THF (5 mL), LiHMDS (0.5 mL, 1 M in THF, 0.5 mmol) was added dropwise and the mixture was stirred overnight. The reaction was quenched by adding 1 M HCl in methanol and the reaction mixture was evaporated. Purification by silica gel flash column chromatography using 5% MeOH in CH_2Cl_2 as eluent yielded the desired product as white solid (61 mg, 30% yield). ^{1}H NMR (500 MHz, $CDCl_3$): δ 8.40 (s, 1H), 7.29 – 7.19 (m, 3H), 7.18 – 7.09 (m, 2H), 6.86 (d, *J* = 7.9 Hz, 1H), 6.30 (s, 1H), 6.24 (d, *J* = 1.8 Hz, 1H), 5.57 (d, *J* = 1.7 Hz, 1H), 4.41 (s, 2H), 2.89 (s, 3H), 1.92 (s, 3H); ^{13}C NMR (126 MHz, $CDCl_3$): δ 173.81, 155.18 (d, *J* = 248.5 Hz), 148.38, 138.17, 137.74 (d, *J* = 11.1 Hz), 136.14, 135.61, 131.32, 131.14, 130.89, 128.65, 127.76, 127.27, 127.04, 124.98, 117.57 (d, *J* = 2.3 Hz), 116.17, 108.97 (d, *J* = 25.5 Hz), 104.14 (d, *J* = 4.5 Hz), 56.07 (d, *J* = 8.9 Hz), 38.67, 17.40.

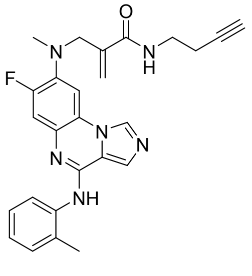

N-(but-3-yn-1-yl)-2-(((7-fluoro-4-(o-tolylamino)imidazo[1,5-a]quinoxalin-8-yl)(methyl)amino)methyl)acrylamide (15). A round bottom flask was charged with **41** (20 mg, 49 μmol), 3-Butyn-1-amine hydrochloride (10.3 mg, 98 μmol) and HATU (22.4 mg, 59 μmol). 1 mL dry DMF added to the flask followed by DIPEA (43 μL, 245 μmol) and the mixture was stirred for 1 h until TLC analysis indicated full conversion. The mixture was concentrated, and the residue was purified by HPLC to get desired product as a solid compound (13.4 mg, 60%). ^{1}H NMR (500 MHz, $CDCl_3$) δ 8.5 (s, 1H), 7.9 (s, 1H), 7.8 (d, *J* = 7.7 Hz, 1H), 7.4 (d, *J* = 7.8 Hz, 1H), 7.4 – 7.4 (m, 1H), 7.3 – 7.3 (m, 2H), 7.3 – 7.2 (m, 1H), 7.2 (s, 1H), 6.2 (d, *J* = 1.3 Hz, 1H), 5.6 (s, 1H), 4.0 (s, 2H), 3.5 (q, *J* = 6.3 Hz, 2H), 2.9 (s, 3H), 2.4 (td, *J* = 6.5, 2.6 Hz, 2H), 2.3 (s, 3H), 1.9 (t, *J* = 2.6 Hz, 1H); ^{13}C NMR (126 MHz, $CDCl_3$) δ 166.83, 155.00 (d, *J* = 246.8 Hz), 147.19, 138.66, 136.88 (d, *J* =

11.5 Hz), 136.14, 132.76, 131.18, 130.60, 127.16, 126.93, 125.73, 125.17, 119.14, 117.56, 112.68 (d, *J* = 24.3 Hz), 105.80 (d, *J* = 4.0 Hz), 81.65, 69.93, 58.25 (d, *J* = 3.6 Hz), 40.38 (d, *J* = 2.4 Hz), 38.19, 19.43, 18.13. HRMS (ESI-TOF): calculated for $C_{26}H_{26}FN_6O$ (M+H): 457.2147, found: 457.2150.

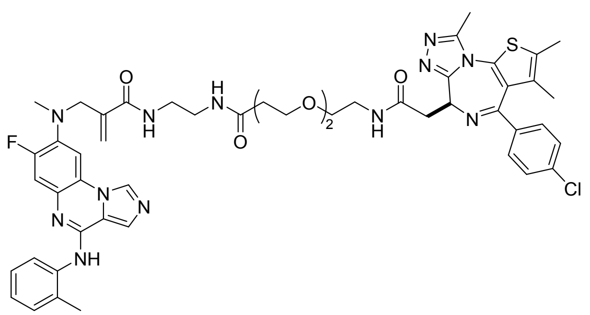

(*S*)-N-(1-(4-(4-chlorophenyl)-2,3,9-trimethyl-6H-thieno[3,2-f][1,2,4]triazolo[4,3-a][1,4]diazepin-6-yl)-2,12-dioxo-6,9-dioxa-3,13-diazapentadecan-15-yl)-2-(((7-fluoro-4-(o-tolylamino)imidazo[1,5-a]quinoxalin-8-yl)(methyl)amino)methyl)acrylamide (**17**). Step1. A round bottom flask was charged with **41** (30 mg, 74 µmol), tert-butyl (2-aminoethyl)carbamate (23.7 mg, 148 µmol) and HATU (33.7 mg, 88 µmol). 1 mL dry DMF was added to the flask followed by DIPEA (51 µL, 296 µmol) and the mixture was stirred for 2 h until TLC analysis indicated full conversion. The mixture was concentrated, and the residue was purified by HPLC to get desired product as a solid compound (25 mg, 62%). The product was treated with TFA (0.5 mL) in CH_2Cl_2 (1 mL) and the reaction was stirred at room temperature for 1 h after which LCMS analysis indicate full deprotection of the Boc group. The reaction mixture was concentrated to get the desired deprotected amine **42** which was used directly in the next step.

Step 2. In a round bottom flask, (*S*)-JQ1-PEG2-acid **43**[i] (25.8 mg, 46 µmol) was mixed with the amine **42** from previous step followed by HATU (21 mg, 55 µmol), and DIPEA (32 µL, 184 µmol). The reaction mixture was stirred for 1h, concentrated under reduced pressure and purified by HPLC to afford the desired product **15 (**21.4 mg, 47%).^{1}H NMR (500 MHz, $CDCl_3$) δ 8.64 (s, 1H), 8.02 (d, *J* = 5.0 Hz, 1H), 7.80 – 7.63 (m, 1H), 7.52 – 7.35 (m, 5H), 7.32 – 7.18 (m, 5H), 5.98 (s, 1H), 5.49 (s, 1H), 4.66 (t, *J* = 7.1 Hz, 1H), 4.00 (s, 2H), 3.73 (t, *J* = 6.0 Hz, 2H), 3.66 – 3.32 (m, 14H), 2.86 (d, *J* = 4.0 Hz, 3H), 2.60 (d, *J* = 2.3 Hz, 3H), 2.49 (dtd, *J* = 15.2, 9.3, 6.1 Hz, 2H), 2.38 (d, *J* = 2.7 Hz, 3H), 2.31 (s, 2H), 1.65 (s, 3H); ^{13}C NMR (126 MHz, $CDCl_3$) δ 172.45, 170.50, 168.05, 163.98, 155.85, 154.33 (d, *J* = 246.1 Hz), 150.02, 146.68, 139.54, 136.98, 136.75 (d, *J* = 31.7 Hz), 136.47, 132.49, 131.99, 131.09, 131.05, 131.01, 130.77, 130.72, 129.96, 128.77, 126.97, 126.40, 125.48, 122.63, 119.27, 117.74, 112.73, 105.12 (d, *J* = 4.4 Hz), 70.37, 70.10, 69.74, 67.27, 57.24 (d, *J* = 3.8 Hz), 54.33, 40.41 (d, *J* = 3.2 Hz), 40.20, 39.43, 39.40, 38.98, 37.06, 18.14, 14.49, 13.19, 11.84. HRMS (ESI-TOF): calculated for $C_{50}H_{54}ClFN_{12}O_5S$ (M+H): 989.3806, found: 989.3798.

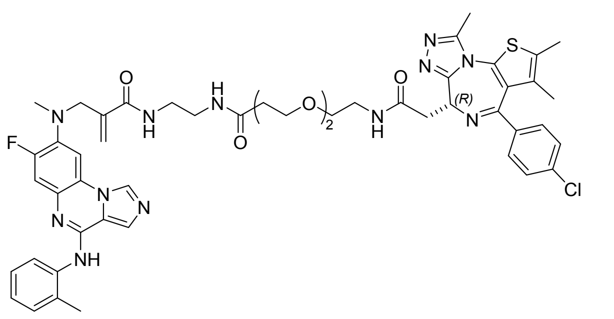

(*R*)-N-(1-(4-(4-chlorophenyl)-2,3,9-trimethyl-6H-thieno[3,2-f][1,2,4]triazolo[4,3-a][1,4]diazepin-6-yl)-2,12-dioxo-6,9-dioxa-3,13-diazapentadecan-15-yl)-2-(((7-fluoro-4-(o-tolylamino)imidazo[1,5-a]quinoxalin-8-yl)(methyl)amino)methyl)acrylamide (**18**). Compound **18** was prepared by following the same procedure as of **17** but (*R*)-JQ1-PEG2-acid was used instead of (*S*)-JQ1-PEG2-acid. Characterization data matched with **17**.

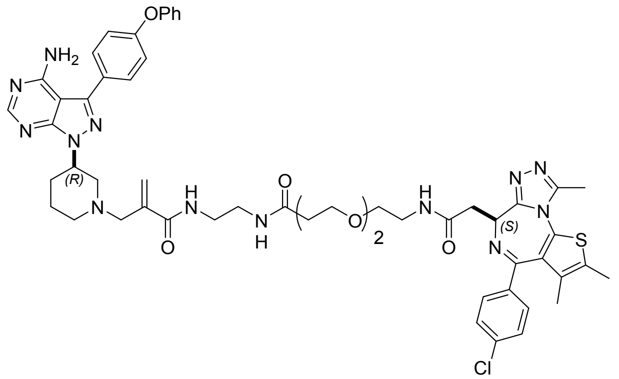

2-(((*R*)-3-(4-amino-3-(4-phenoxyphenyl)-1*H*-pyrazolo[3,4-d]pyrimidin-1-yl)piperidin-1-yl)methyl)-*N*-(1-((*S*)-4-(4-chlorophenyl)-2,3,9-trimethyl-6*H*-thieno[3,2-f][1,2,4]triazolo[4,3-a][1,4]diazepin-6-yl)-2,12-dioxo-6,9-dioxa-3,13-diazapentadecan-15-yl)acrylamide (19)

To a stirred solution of (*S*)-JQ1-PEG2-acid **43** (30.0 mg, 0.05 mmol) in CH_2Cl_2 (0.5 mL), HATU (22.8 mg, 0.06 mmol), DIPEA (21.9 µL, 0.12 mmol) and (R)-2-((3-(4-amino-3-(4-phenoxyphenyl)-1H-pyrazolo[3,4-d]pyrimidin-1-yl)piperidin-1-yl)methyl)-N-(2-aminoethyl)acrylamide[ii] (25.6 mg, 0.05 mmol) were added at 25 °C. The reaction mixture was stirred at room temperature for 1 h. The reaction mixture was monitored by LC-MS. After the completion, solvent was concentrated in *vacuo* and the crude product was purified by preparative HPLC using water: MeCN (0.1% formic acid) solvent gradient to afford **19** as white solid (29.0 mg, 55%). ^{1}H NMR (400 MHz, $CDCl_3$) δ 9.47 – 9.36 (m, 2H), 8.68 (t, *J* = 5.4 Hz, 1H), 8.52 (s, 1H), 7.63 – 7.53 (m, 2H), 7.43 – 7.36 (m, 4H), 7.32 (d, *J* = 8.7 Hz, 2H), 7.20 – 7.14 (m, 1H), 7.12 – 7.05 (m, 4H), 6.28 (d, *J* = 1.8 Hz, 1H), 5.42 (s, 1H), 4.95 (tt, *J* = 11.5, 3.9 Hz, 1H), 4.77 (dd, *J* = 9.2, 5.5 Hz, 1H), 3.95 – 3.52 (m, 13H), 3.37 (tt, *J* = 14.3, 8.0 Hz, 3H), 3.19 – 2.87 (m, 5H), 2.66 (s, 3H), 2.45 (t, *J* = 4.2 Hz, 2H), 2.42 (s, 3H), 2.40 – 1.97 (m, 6H), 1.86 – 1.73 (m, 1H), 1.69 (s, 3H). ^{13}C NMR (101 MHz, $CDCl_3$) δ 173.34, 170.71, 166.66, 164.32, 164.17, 158.43, 158.27, 156.38, 156.12, 155.57, 153.56, 149.86, 144.49, 137.18, 136.87, 136.51, 132.02, 131.00, 130.59, 129.96, 129.93, 129.89, 128.74, 127.63, 126.77, 123.96, 119.54, 119.17, 98.25, 70.86, 70.61, 70.51, 67.81, 61.16, 56.19, 54.84, 53.80, 53.49, 40.98, 39.53, 38.97, 38.67, 37.02, 29.24, 24.99, 14.42, 13.13, 11.78. HRMS (ESI-TOF): calculated for $C_{54}H_{61}Cl_1N_{13}O_6S_1$ (M+H): 1054.4272, found: 1054.4280.

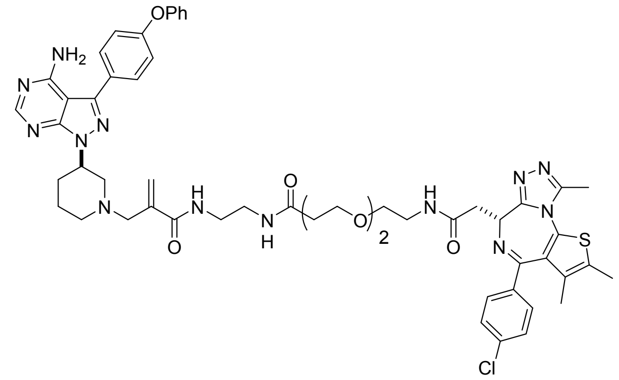

2-(((*R*)-3-(4-amino-3-(4-phenoxyphenyl)-1*H*-pyrazolo[3,4-d]pyrimidin-1-yl)piperidin-1-yl)methyl)-*N*-(1-((*R*)-4-(4-chlorophenyl)-2,3,9-trimethyl-6*H*-thieno[3,2-f][1,2,4]triazolo[4,3-a][1,4]diazepin-6-yl)-2,12-dioxo-6,9-dioxa-3,13-diazapentadecan-15-yl)acrylamide (20)

To a stirred solution of (*R*)-JQ1-PEG2-acid [3] (30.0 mg, 0.05 mmol) in CH_2Cl_2 (0.5 mL), HATU (22.8 mg, 0.06 mmol), DIPEA (21.9 µL, 0.12 mmol) and (R)-2-((3-(4-amino-3-(4-phenoxyphenyl)-1H-pyrazolo[3,4-d]pyrimidin-1-yl)piperidin-1-yl)methyl)-N-(2-aminoethyl)acrylamide[4] (25.6 mg, 0.05 mmol) were added at 25 °C. The reaction mixture was stirred at room temperature for 1 h. The reaction mixture was monitored by LC-MS. After the completion, solvent was concentrated in *vacuo* and the crude product was purified by preparative HPLC using water : MeCN (0.1% formic acid) solvent gradient to afford **20** as a white solid (27.2 mg, 52%). ^{1}H-NMR (400 MHz, $CDCl_3$) δ 9.39 (dd, *J* = 8.0, 4.4 Hz, 1H), 8.98 (t, *J* = 5.6 Hz, 1H), 8.57 (s, 1H), 8.31 (t, *J* = 5.4 Hz, 1H), 7.58 – 7.51 (m, 2H), 7.38 (dt, *J* = 8.4, 3.7 Hz, 4H), 7.32 (d, *J* = 8.7 Hz, 2H), 7.21 – 7.15 (m, 1H), 7.13 – 7.02 (m, 4H), 6.28 (d, *J* = 1.9 Hz, 1H), 5.43 (s, 1H), 4.95 (tt, *J* = 11.1, 3.9 Hz, 1H), 4.74 (t, *J* = 7.1 Hz, 1H), 3.94 – 3.80 (m, 2H), 3.80 – 3.47 (m, 13H), 3.39 (tt, *J* = 13.6, 3.6 Hz, 1H), 3.23 – 3.04 (m, 3H), 3.03 – 2.89 (m, 2H), 2.67 (s, 3H), 2.48 – 2.38 (m, 5H), 2.30 – 1.93 (m, 6H), 1.78 (q, *J* = 13.3 Hz, 1H), 1.67 (s, 3H). ^{13}C-NMR (100 MHz, $CDCl_3$) δ 173.22, 170.70, 166.77, 164.30, 158.39, 158.13, 156.41, 156.37, 155.55, 153.67, 149.90, 144.27, 137.25, 136.91, 136.50, 132.29, 130.98, 130.95, 130.35, 129.93, 129.89, 128.75, 127.70, 126.64, 123.94, 119.51, 119.13, 98.22, 70.68, 70.56, 67.80, 61.21, 56.30, 54.82, 53.68, 53.30, 40.98, 39.67, 39.01, 38.68, 37.06, 29.46, 24.90, 14.44, 13.13, 11.85. HRMS (ESI-TOF): calculated for $C_{54}H_{61}C_{l1}N_{13}O_6S_1$ (M+H): 1054.4272, found: 1054.4279.

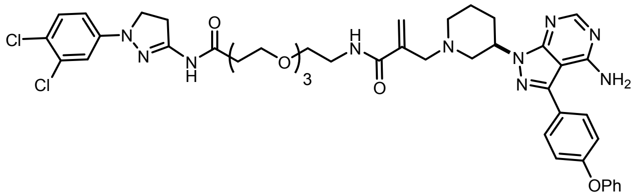

(R)-2-((3-(4-amino-3-(4-phenoxyphenyl)-1H-pyrazolo[3,4-d]pyrimidin-1-yl)piperidin-1-yl)methyl)-N-(2-(2-(2-(3-((1-(3,4-dichlorophenyl)-4,5-dihydro-1H-pyrazol-3-yl)amino)-3-oxopropoxy)ethoxy)ethoxy)ethyl)acrylamide (21). *Step 1*: To a stirred solution of (*R*)-2-((3-(4-amino-3-(4-phenoxyphenyl)-1H-pyrazolo[3,4-d]pyrimidin-1-yl)piperidin-1-yl)methyl)acrylic acid **46** (230 mg, 0.48 mmol) in dichloromethane (6 mL), HATU (190 mg, 0.5 mmol), DIPEA (104 µL, 0.6 mmol) and *tert*-butyl 3-(2-(2-(2-aminoethoxy)ethoxy)ethoxy)propanoate (140 mg, 0.5 mmol) were added at 25 °C. The reaction mixture was

stirred at room temperature for 1 h. After completion of the reaction (as monitored by LC-MS), 20 mL of water was added. The aqueous layer was extracted with CH_2Cl_2 (3 × 20 mL). The combined organic layers were concentrated in vacuo and the crude product was purified by flash column chromatography (gradient CH_2Cl_2: MeOH from 100:0 to 90:10) affording 295 mg of product **47** that was used in the next step without further characterization. *Step 2*: The product after step 1 (295 mg, 0.4 mmol) was dissolved in CH_2Cl_2 (10 mL) and treated with TFA (2 mL). After 30 min stirring at room temperature, the reaction mixture was concentrated under reduced pressure to afford **48** which was used directly in the next step without further purification or characterization. *Step 3*: Crude **48** after step 2 (~0.4 mmol) was redissolved in CH_2Cl_2 (4.5 mL) and treated with HATU (175 mg, 0.46 mmol) followed by DIPEA (500 µL). After stirring for 30 min at room temperature 1-(3,4-dichlorophenyl)-4,5-dihydro-1H-pyrazol-3-amine **32** (80 mg, 0.35 mmol) was added. The reaction mixture was stirred at room temperature overnight. After completion of the reaction (as monitored by LC-MS), 20 mL of water was added. The aqueous layer was extracted with CH_2Cl_2 (3 × 20 mL). The combined organic layers were concentrated in vacuo and the crude product was purified by flash column chromatography (gradient CH_2Cl_2 : MeOH from 100:0 to 90:10) and HPLC affording 15 mg (4.8% yield) of the desired product **21** as a white solid. ^{1}H NMR (400 MHz, acetone-d6) δ 9.96 (s, 1H), 9.20 (t, J = 5.6 Hz, 1H), 8.30 (s, 1H), 7.75 (d, J = 8.4 Hz, 2H), 7.44 (t, J = 7.8 Hz, 2H), 7.28 (d, J = 8.9 Hz, 1H), 7.24 – 7.09 (m, 5H), 7.01 (d, J = 2.7 Hz, 1H), 6.77 (dd, J = 8.9, 2.7 Hz, 1H), 6.09 (d, J = 2.4 Hz, 1H), 5.42 (d, J = 2.3 Hz, 1H), 5.00 (tt, J = 10.3, 4.7 Hz, 1H), 3.84 – 3.66 (m, 8H), 3.60 (m, 6H), 3.47 (m, 3H), 3.42 – 3.27 (m, 3H), 3.13 (dd, J = 10.9, 4.0 Hz, 1H), 2.70 – 2.58 (m, 3H), 1.98 – 1.77 (m, 2H). ^{13}C NMR (101 MHz, acetone-d_6) δ 170.53, 166.73, 159.28, 158.80, 157.71, 156.67, 155.27, 151.25, 147.96, 144.29, 139.98, 132.76, 131.30, 131.01, 130.90, 129.50, 124.91, 124.67, 120.10, 120.03, 119.90, 114.39, 113.06, 98.80, 71.42, 71.13, 71.07, 70.71, 67.48, 61.76, 57.84, 54.40, 53.14, 48.98, 39.75, 37.90, 33.57, 30.59, 25.15. HRMS (ESI-TOF): calculated for $C_{44}H_{51}Cl_2N_{10}O_6$ (M+H): 885.3365, found: 885.3369.

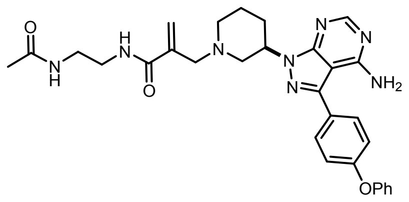

(*R*)-N-(2-acetamidoethyl)-2-((3-(4-amino-3-(4-phenoxyphenyl)-1H-pyrazolo[3,4-d]pyrimidin-1-yl)piperidin-1-yl)methyl)acrylamide (22). (*R*)-2-((3-(4-amino-3-(4-phenoxyphenyl)-1H-pyrazolo[3,4-d]pyrimidin-1-yl)piperidin-1- yl)methyl)-N-(2-aminoethyl)acrylamide[iii] (26 mg, 50 µmol) was dissolved in DMF (1 mL) and treated with succinimidyl acetate (10 mg, 64 µmol) and DIPEA (50 µL, 287 µmol). The reaction mixture was stirred at rt for 30 min, concentrated under reduced pressure, and purified by HPLC affording 16 mg (58% yield,) of the desired product **22** as a white powder. ^{1}H NMR (400 MHz, MeOH-d4) δ 8.24 (s, 1H), 8.15 (s, 2H), 7.78 – 7.61 (m, 2H), 7.45 – 7.27 (m, 2H), 7.20 – 6.96 (m, 5H), 6.08 (s, 1H), 5.73 (s, 1H), 5.09 (s, 1H), 3.72 (d, J = 12.4 Hz, 1H), 3.61 (d, J = 13.0 Hz, 1H), 3.42 – 3.17 (m, 9H), 3.12 – 2.98 (m, 1H), 2.22 – 2.04 (m, 2H), 2.00 (m, 3H), 1.89 (m, 4H). ^{13}C NMR (101 MHz, MeOH-d4) δ 173.75, 169.38, 165.87, 160.07, 159.99, 157.91, 156.86, 154.89, 146.20, 131.45, 131.12, 128.53, 127.61, 125.15, 120.58, 119.91, 99.14, 61.25, 56.84, 53.62, 53.36,

40.29, 40.01, 29.56, 22.68 ppm. HRMS (ESI-TOF): calculated for $C_{30}H_{35}N_8O_3$ (M+H): 555.2827, found: 555.2825.

1. 2. ^{1}H and ^{13}C NMR spectra.

33: ^{1}H NMR (400 MHz, $CDCl_3$):

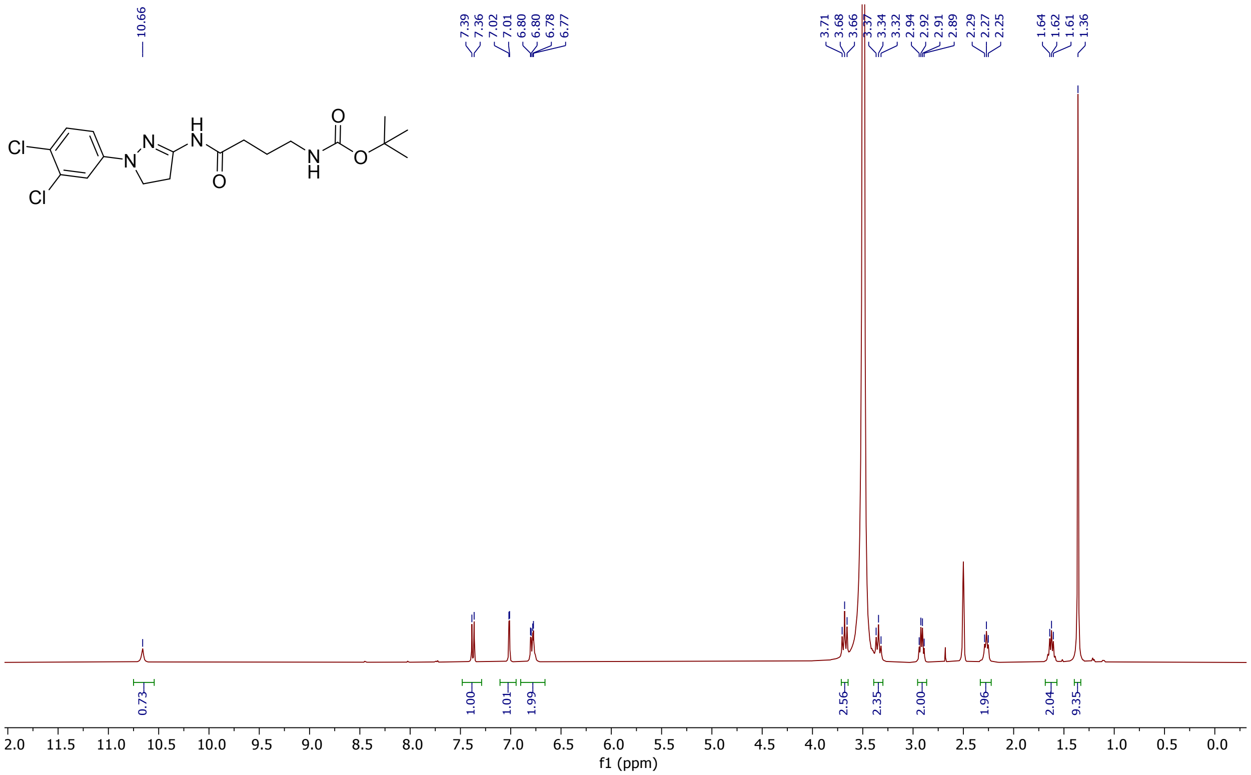

33: ^{13}C NMR (101 MHz, $CDCl_3$):

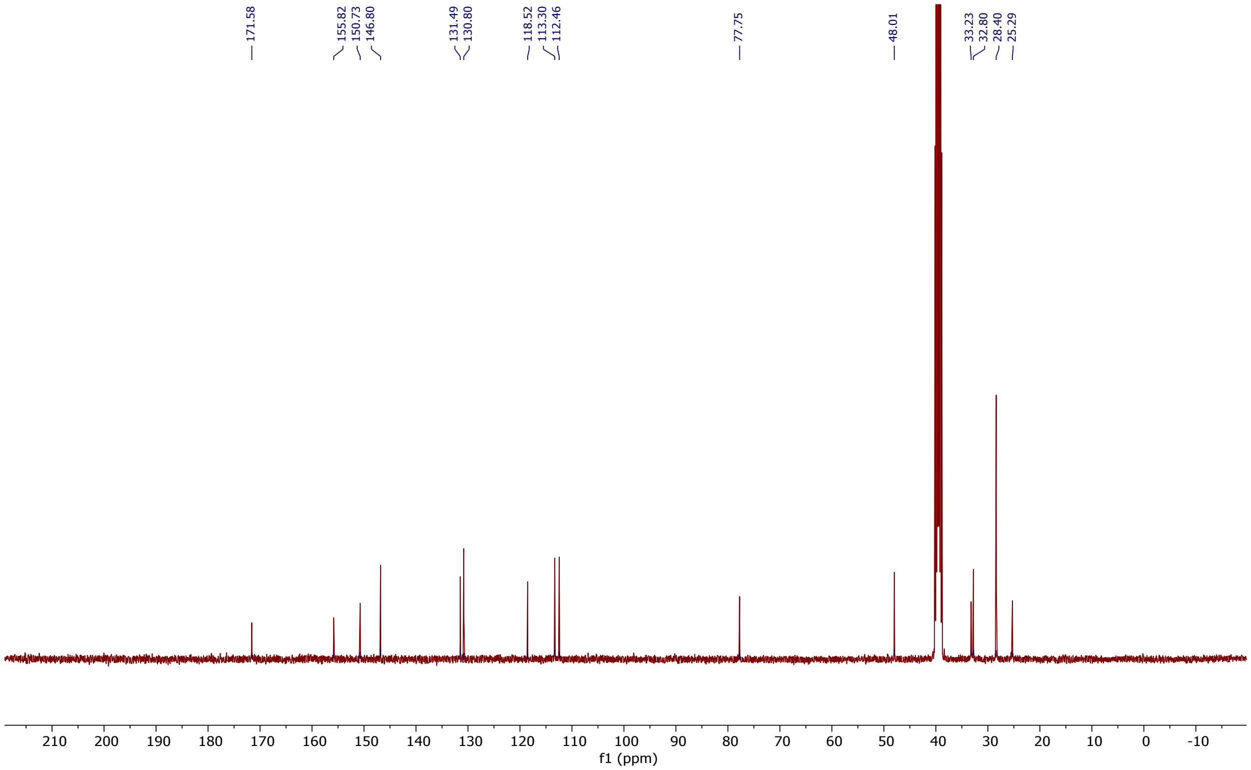

1: ^{1}H NMR (400 MHz, $CDCl_3$):

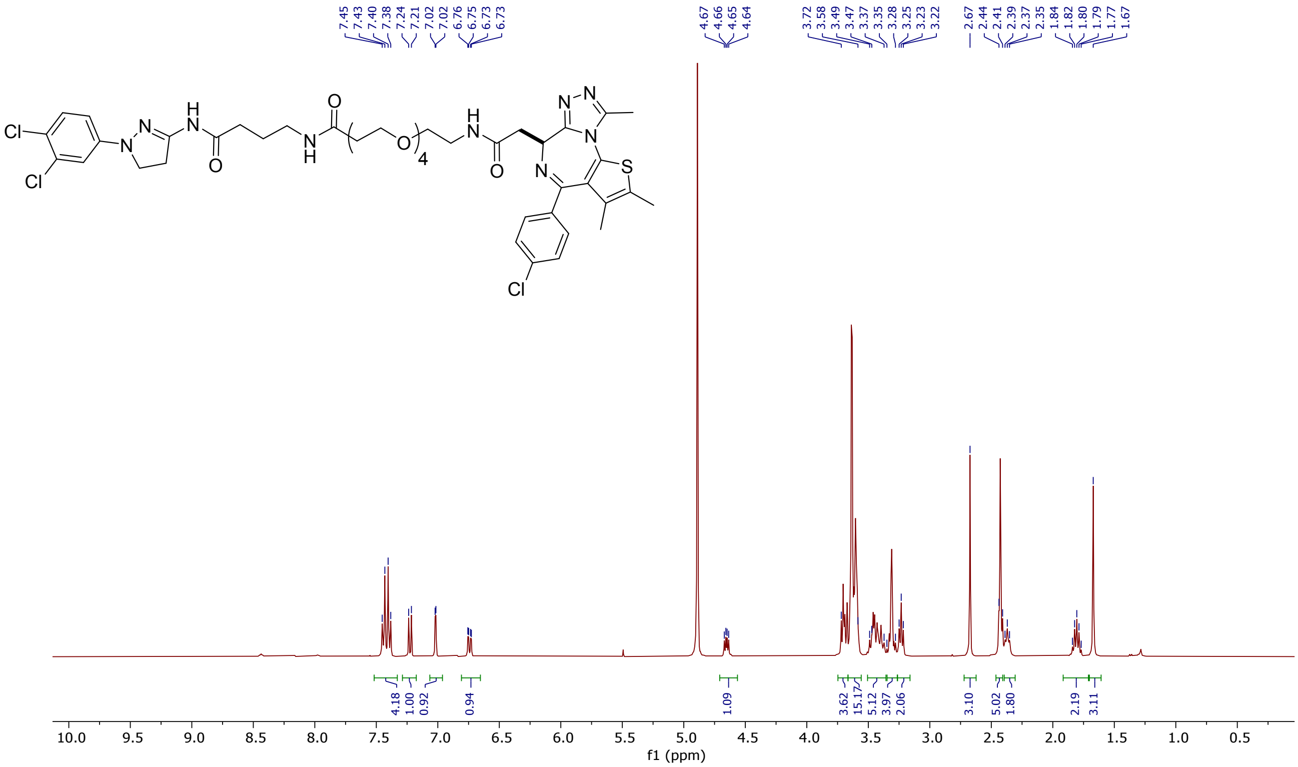

1: ^{13}C NMR (101 MHz, $CDCl_3$):

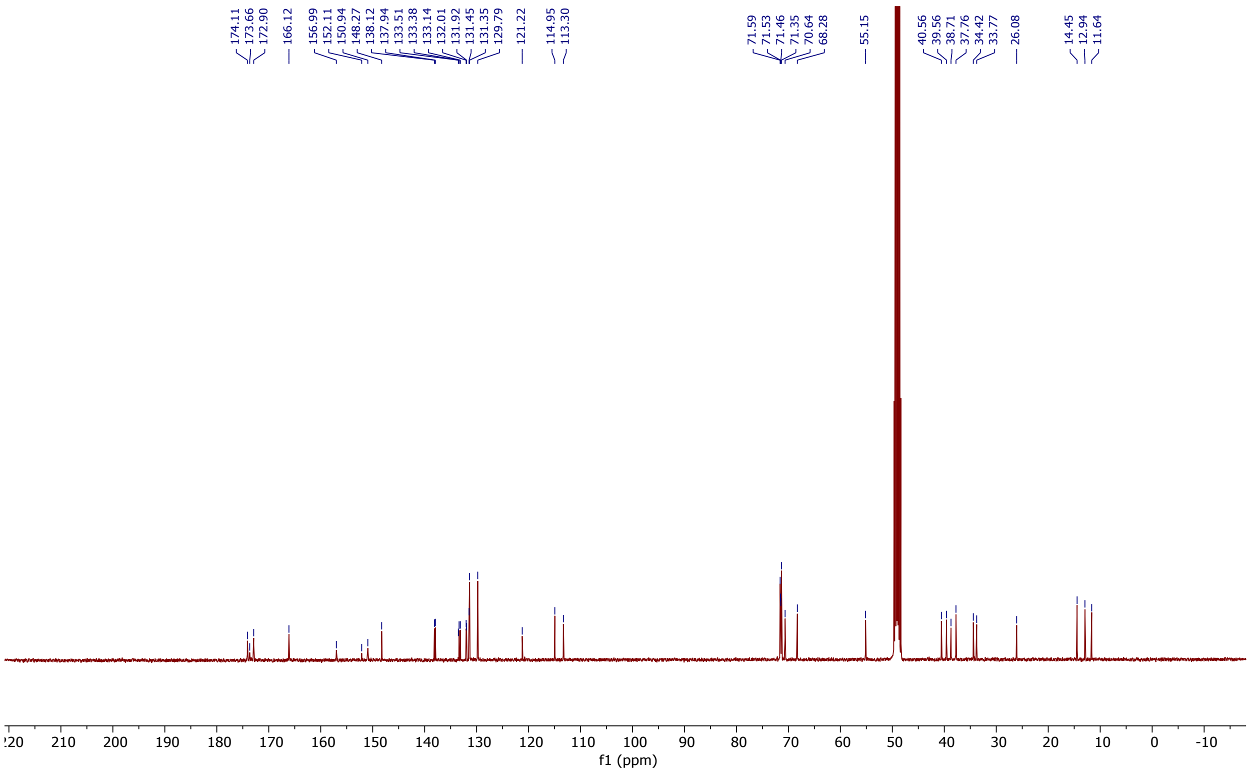

2: ^{1}H NMR (400 MHz, $CDCl_3$):

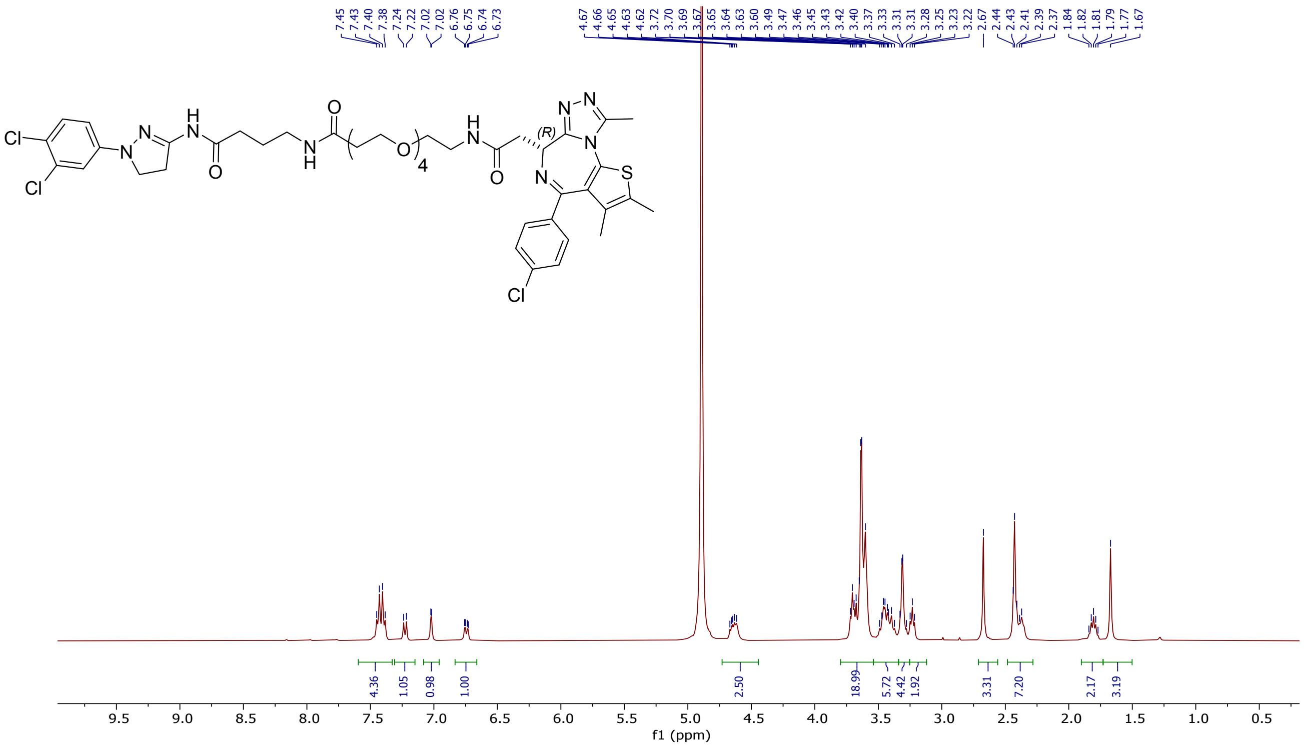

2: ^{13}C NMR (101 MHz, $CDCl_3$):

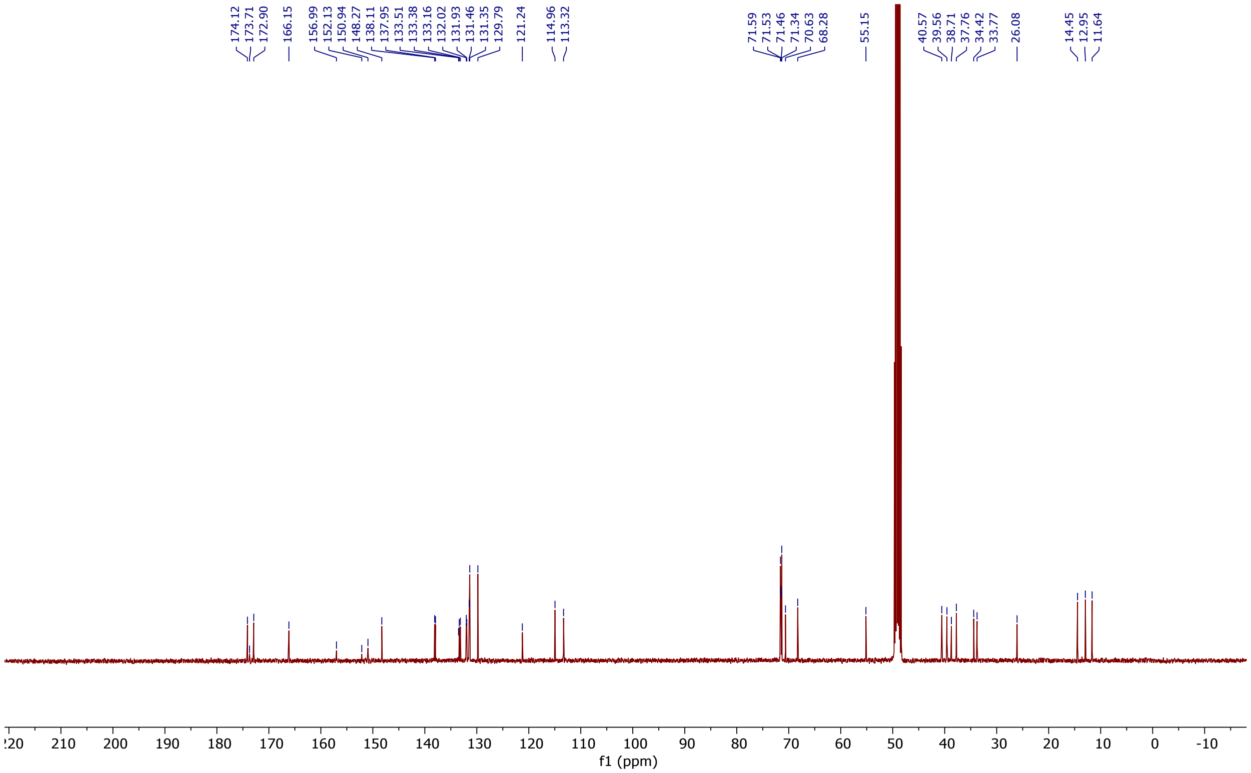

34: ^{1}H NMR (400 MHz, $CDCl_3$):

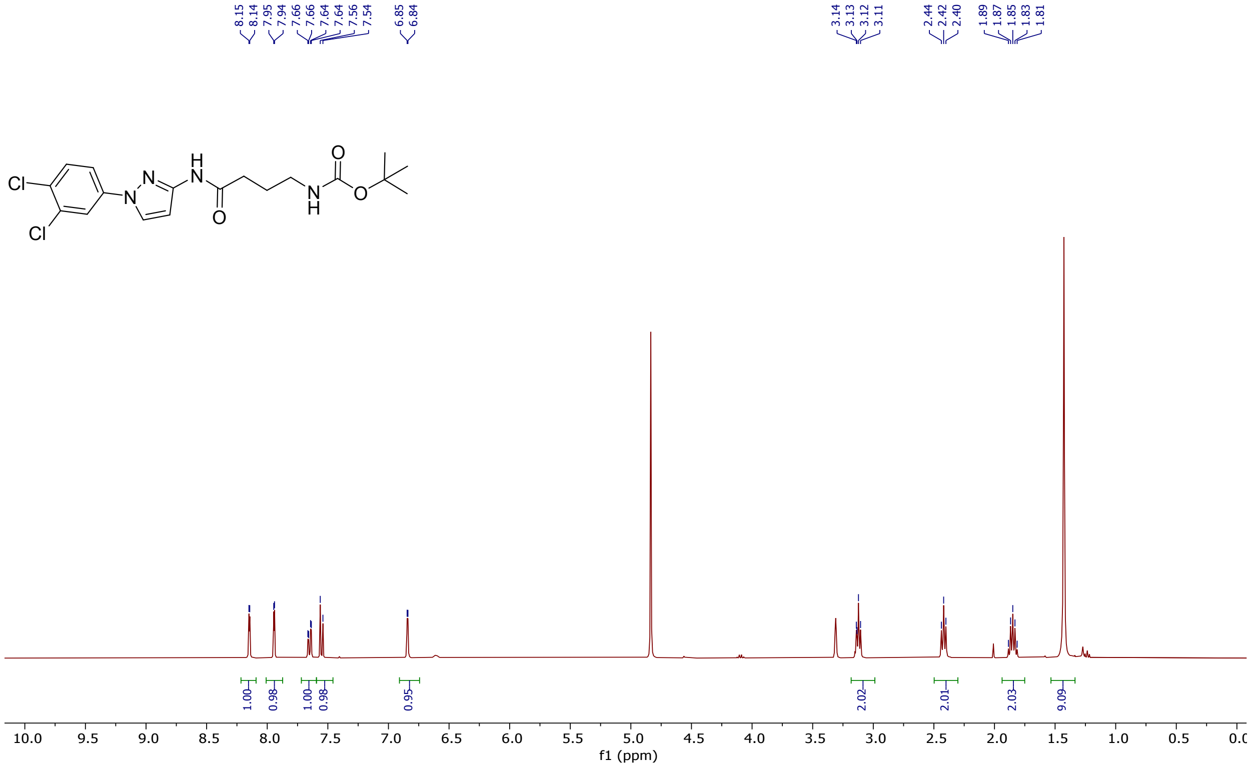

34: ^{13}C NMR (101 MHz, $CDCl_3$):

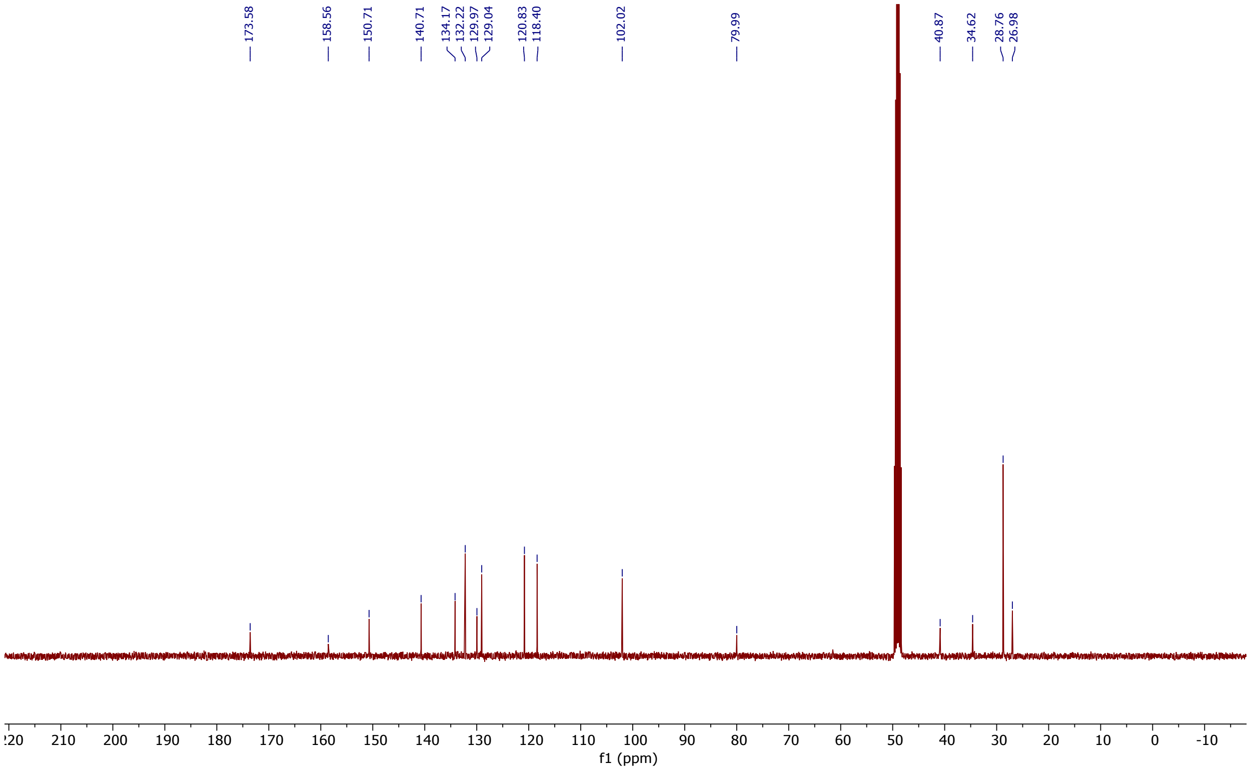

3: ^{1}H NMR (400 MHz, $CDCl_3$):

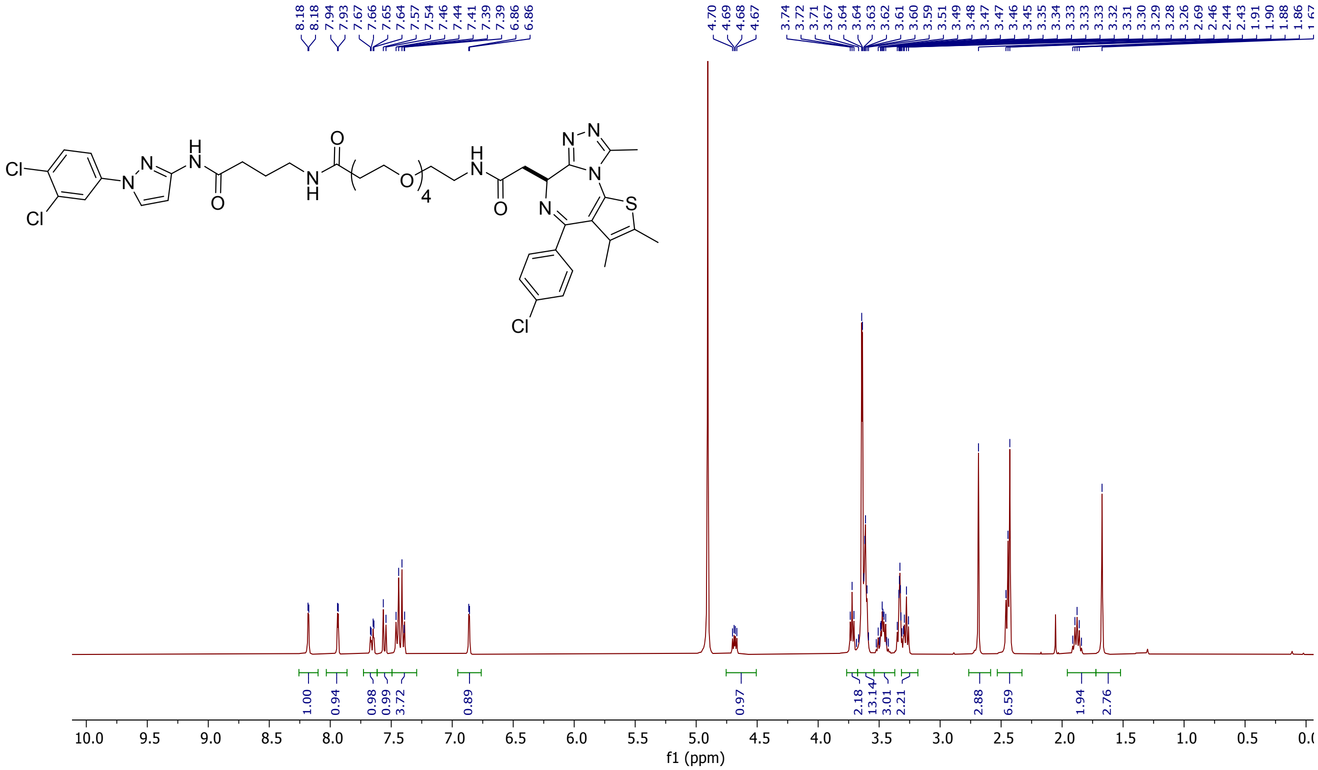

3: ^{13}C NMR (101 MHz, $CDCl_3$):

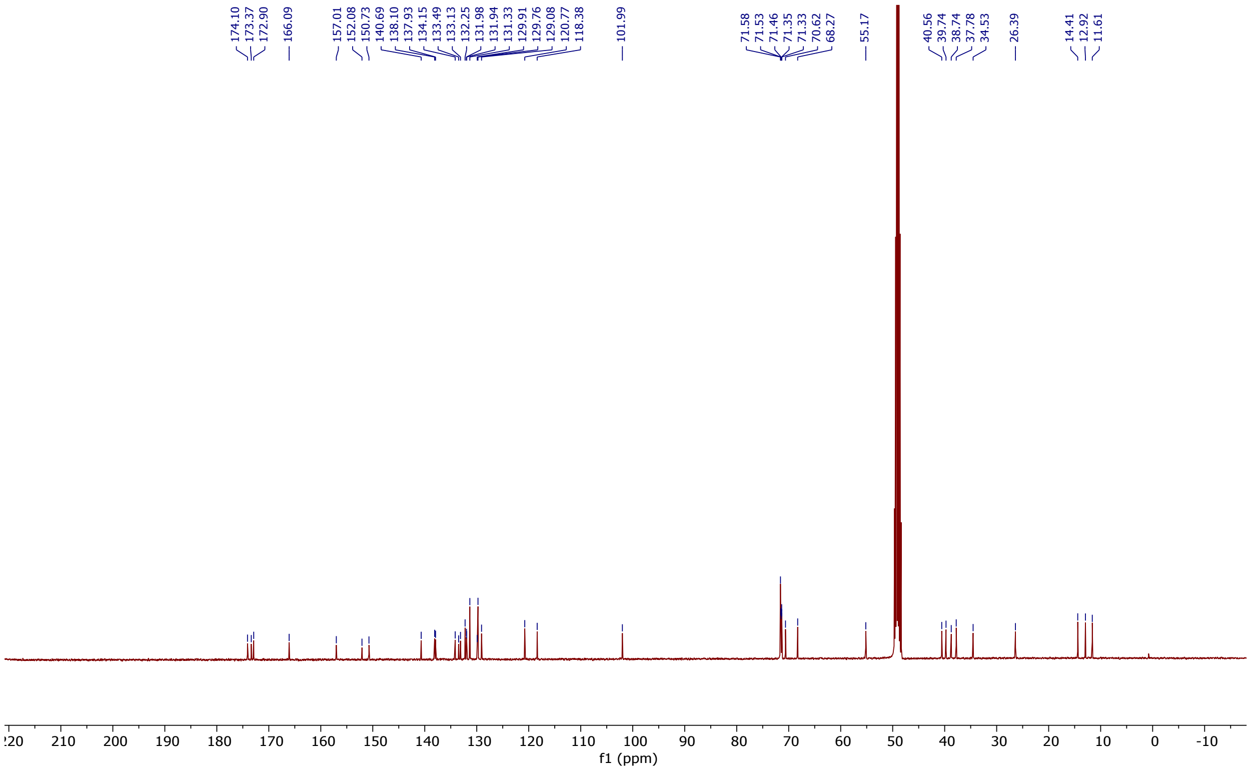

4: ^{1}H NMR (400 MHz, $CDCl_3$):

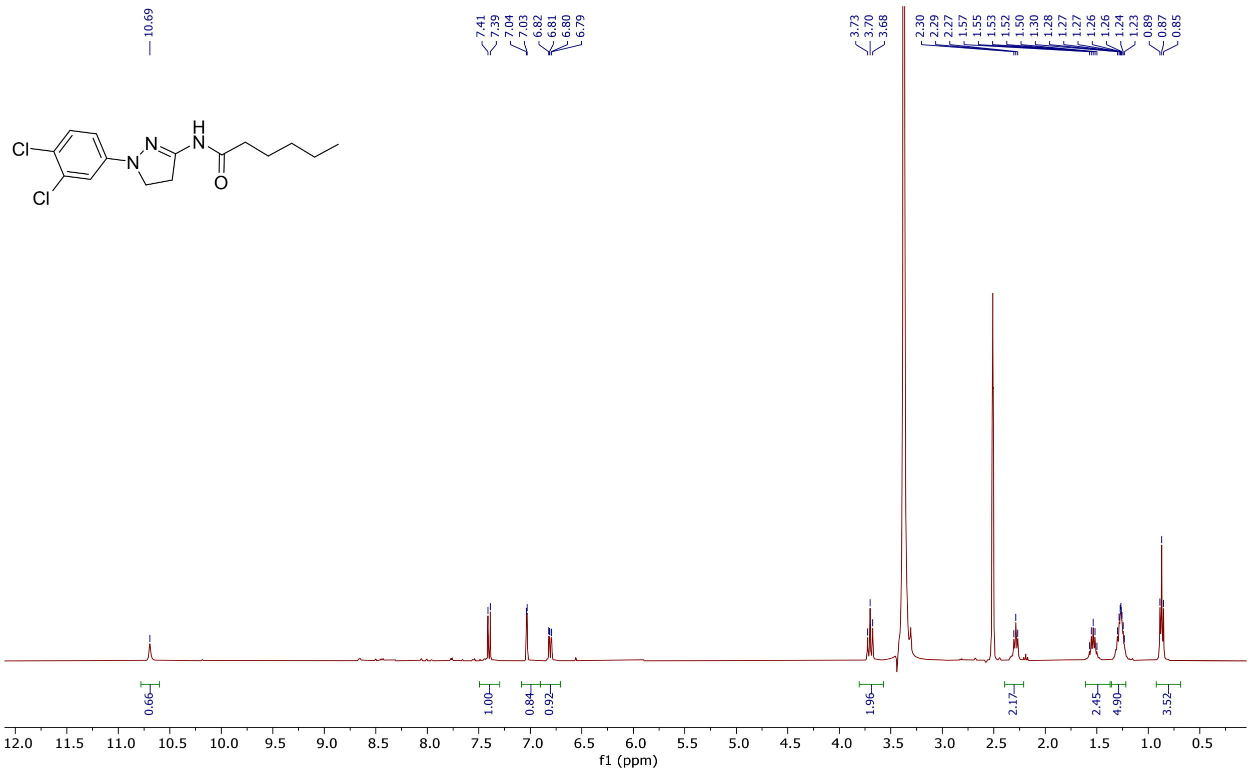

4: ^{13}C NMR (101 MHz, $CDCl_3$):

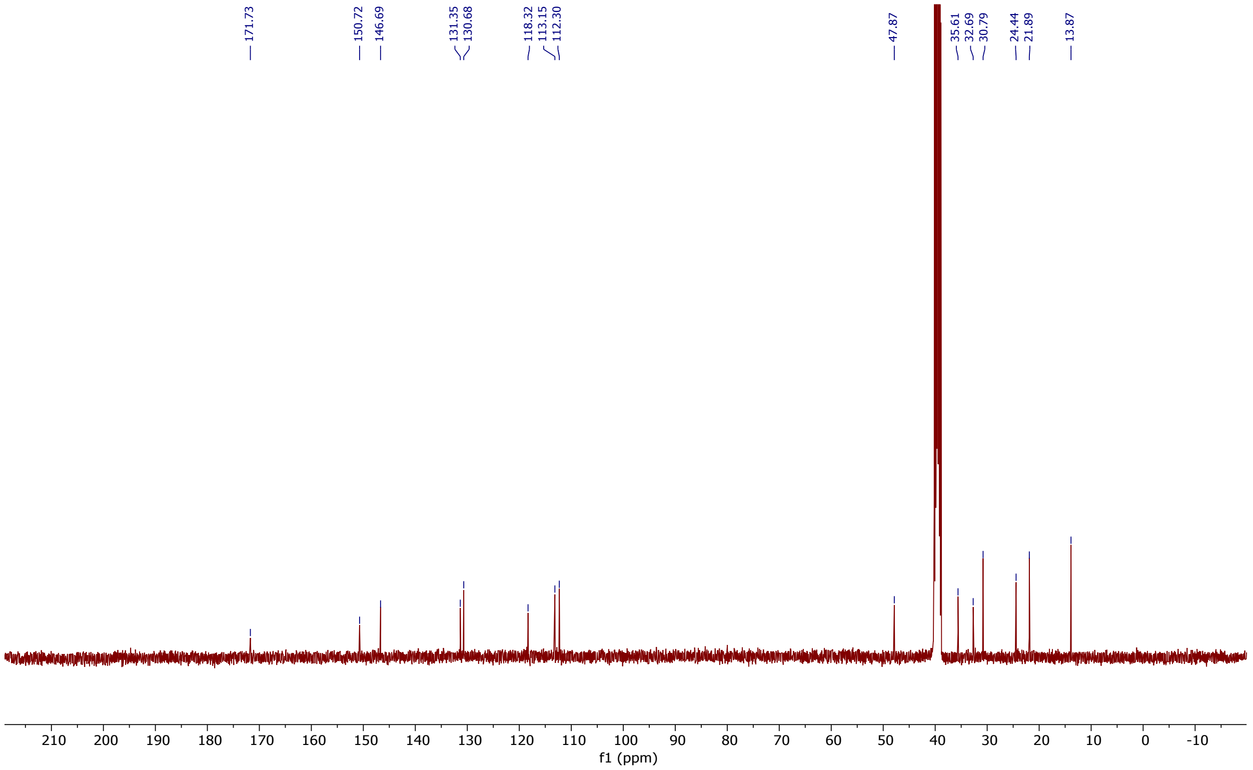

5: ^{1}H NMR (400 MHz, $CDCl_3$):

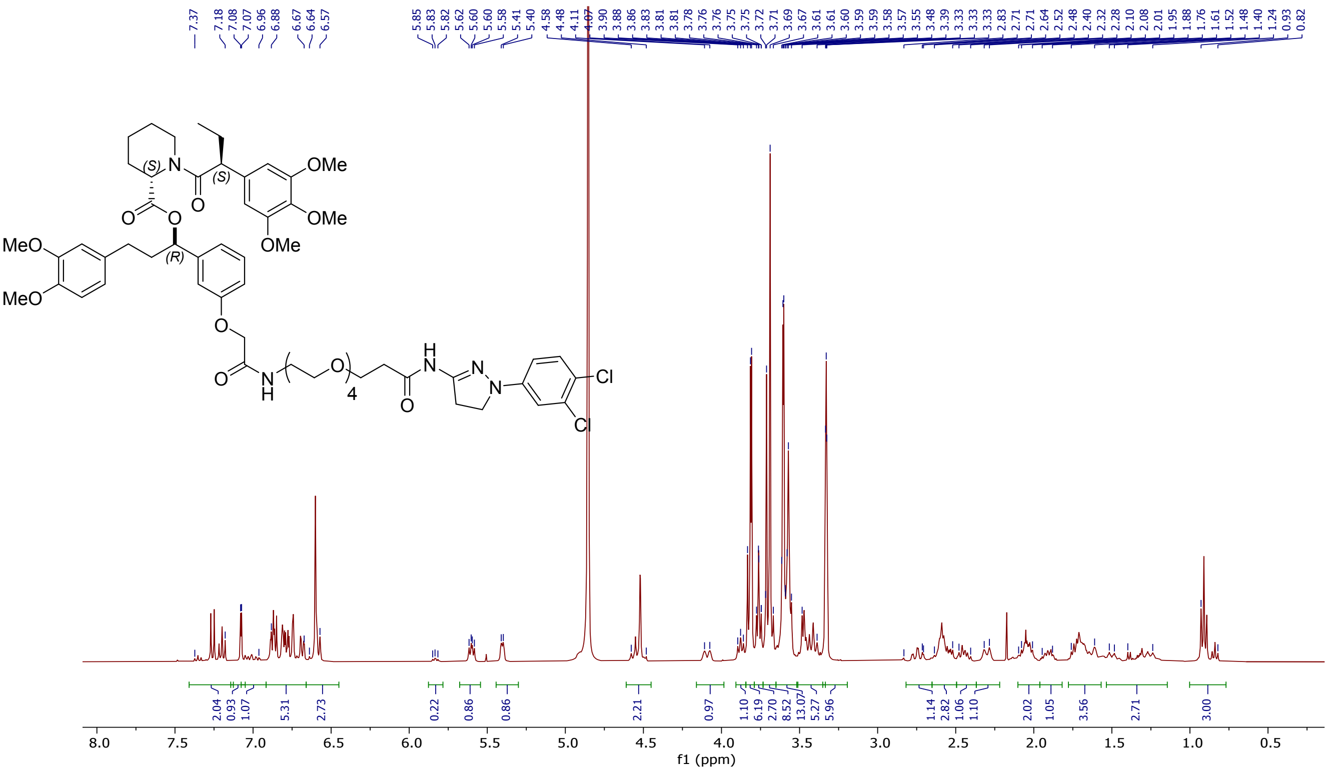

5: ^{13}C NMR (101 MHz, $CDCl_3$):

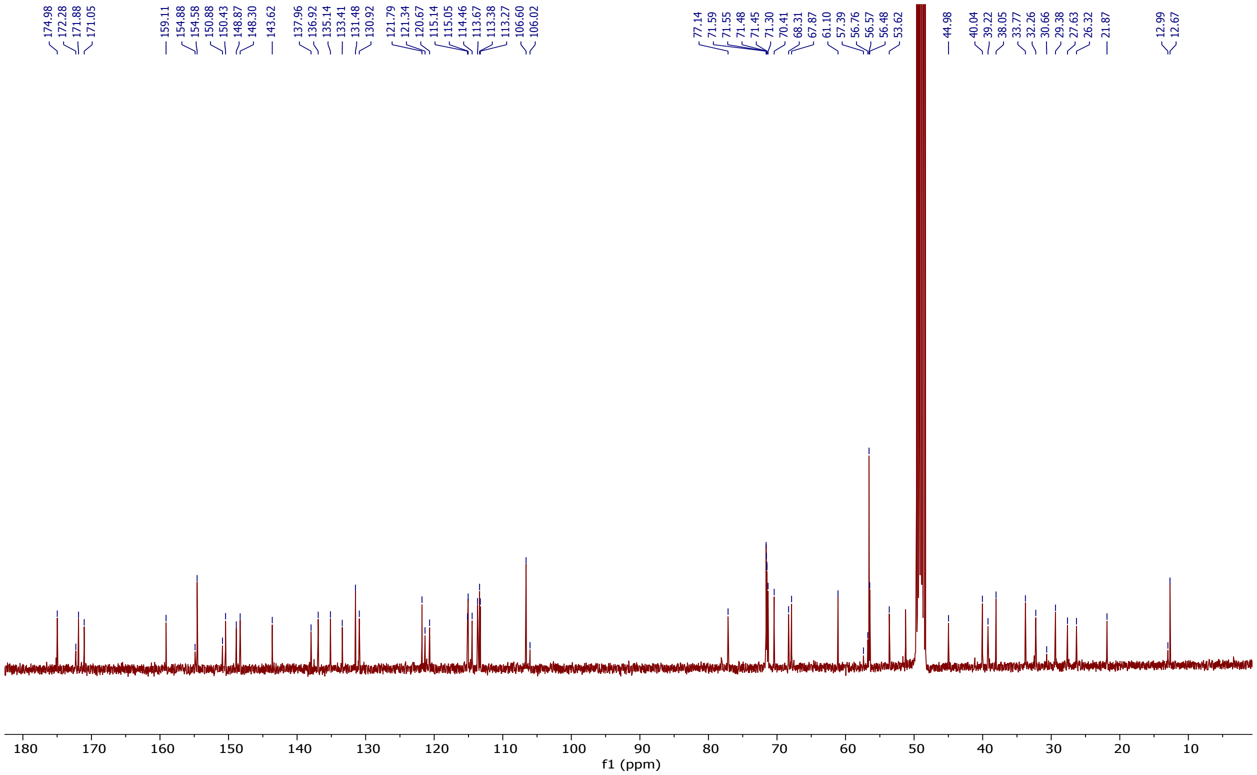

25a: ^{1}H NMR (400 MHz, $CDCl_3$):

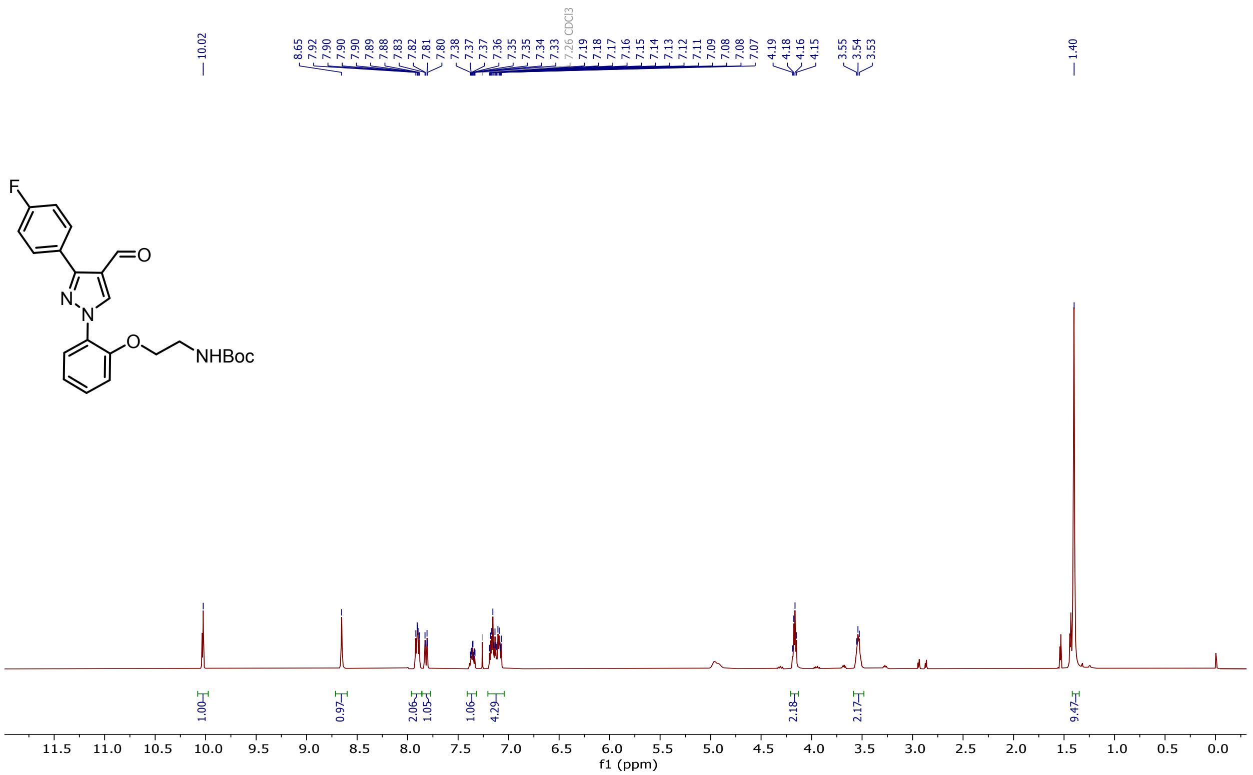

25a: ^{13}C NMR (101 MHz, $CDCl_3$):

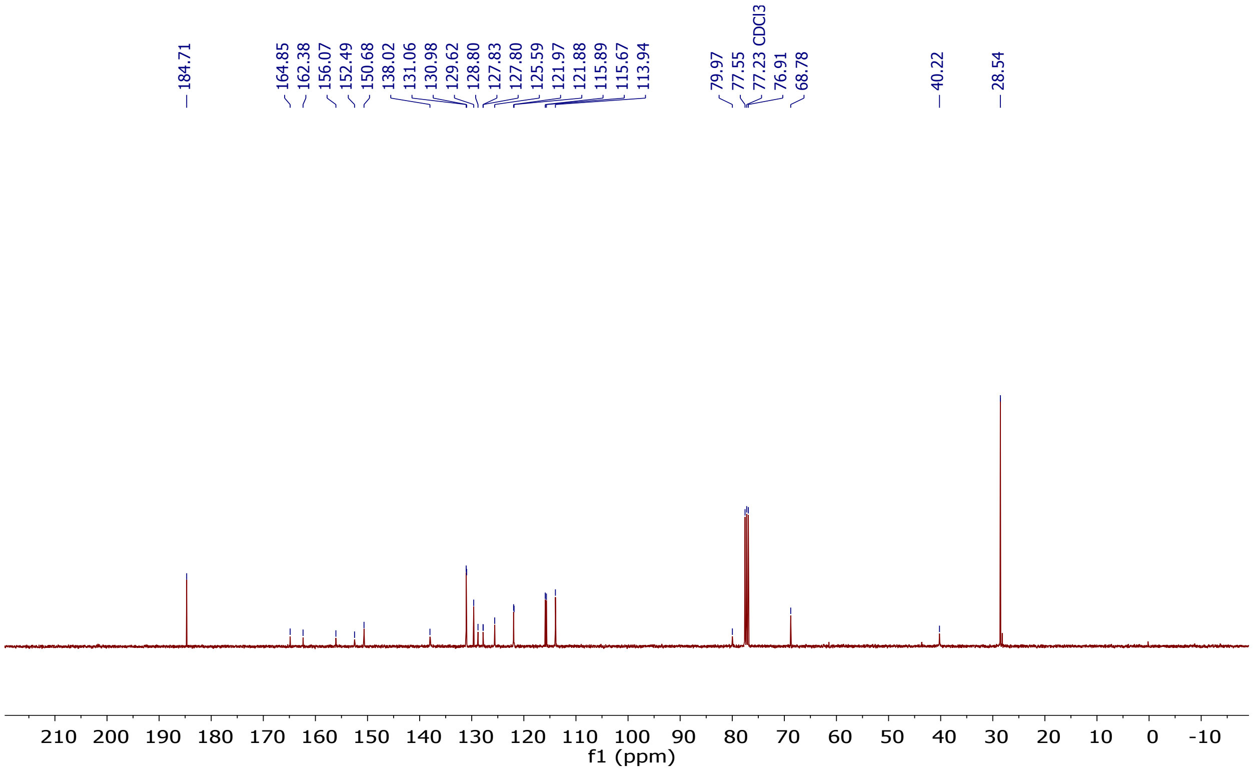

25b: ^{1}H NMR (400 MHz, $CDCl_3$):

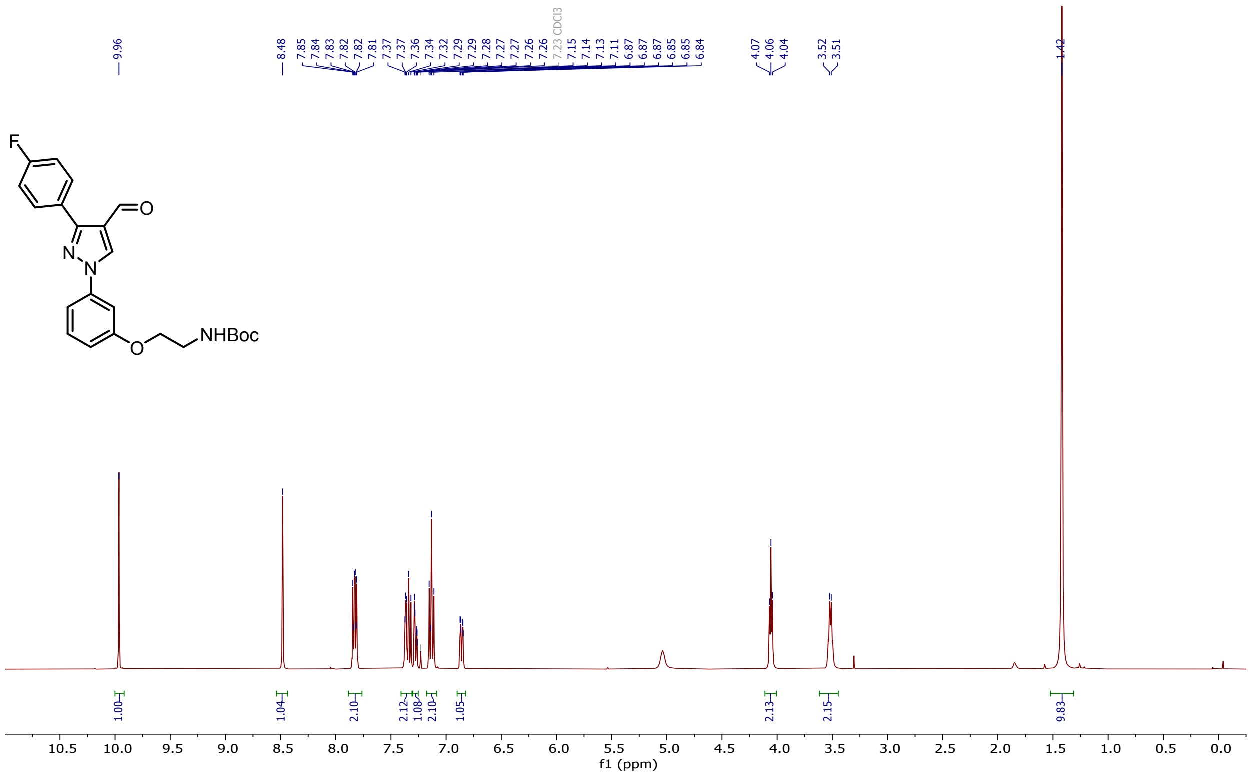

25b: ^{13}C NMR (101 MHz, $CDCl_3$):

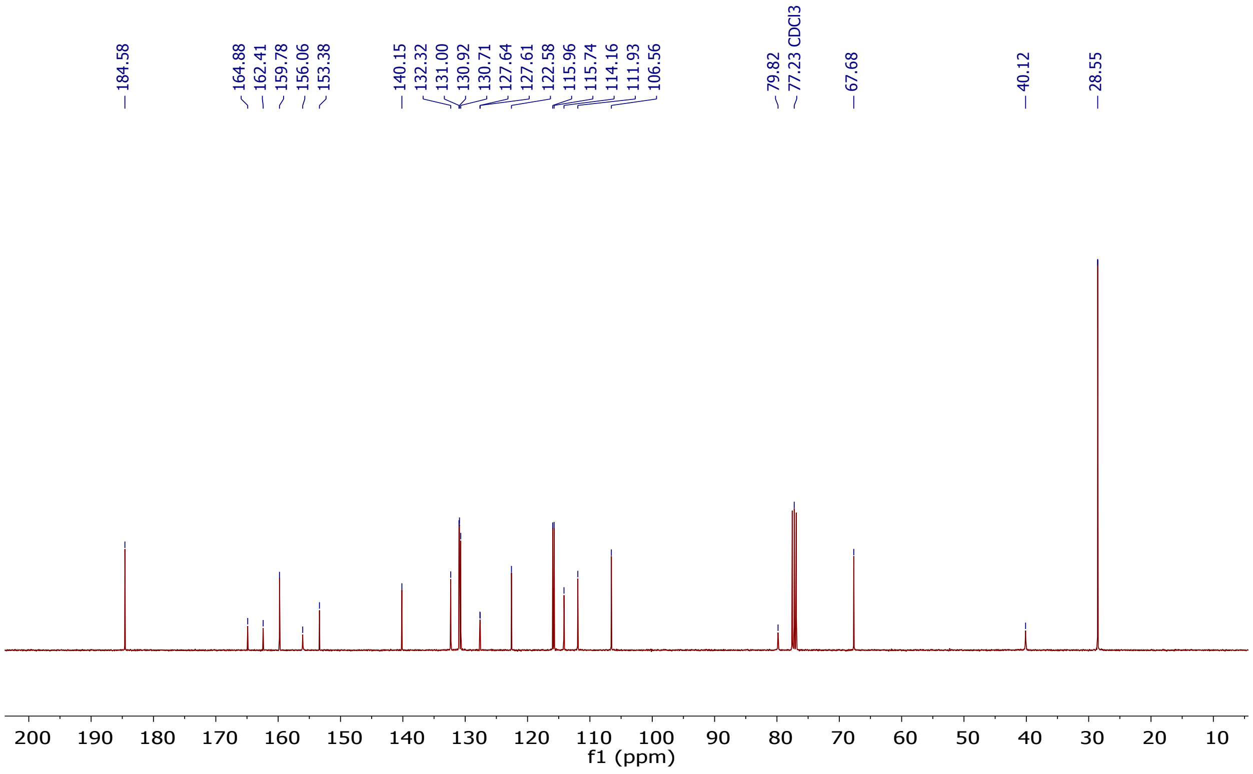

25c: ^{1}H NMR (400 MHz, $CDCl_3$):

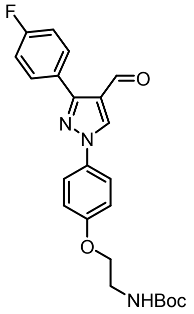

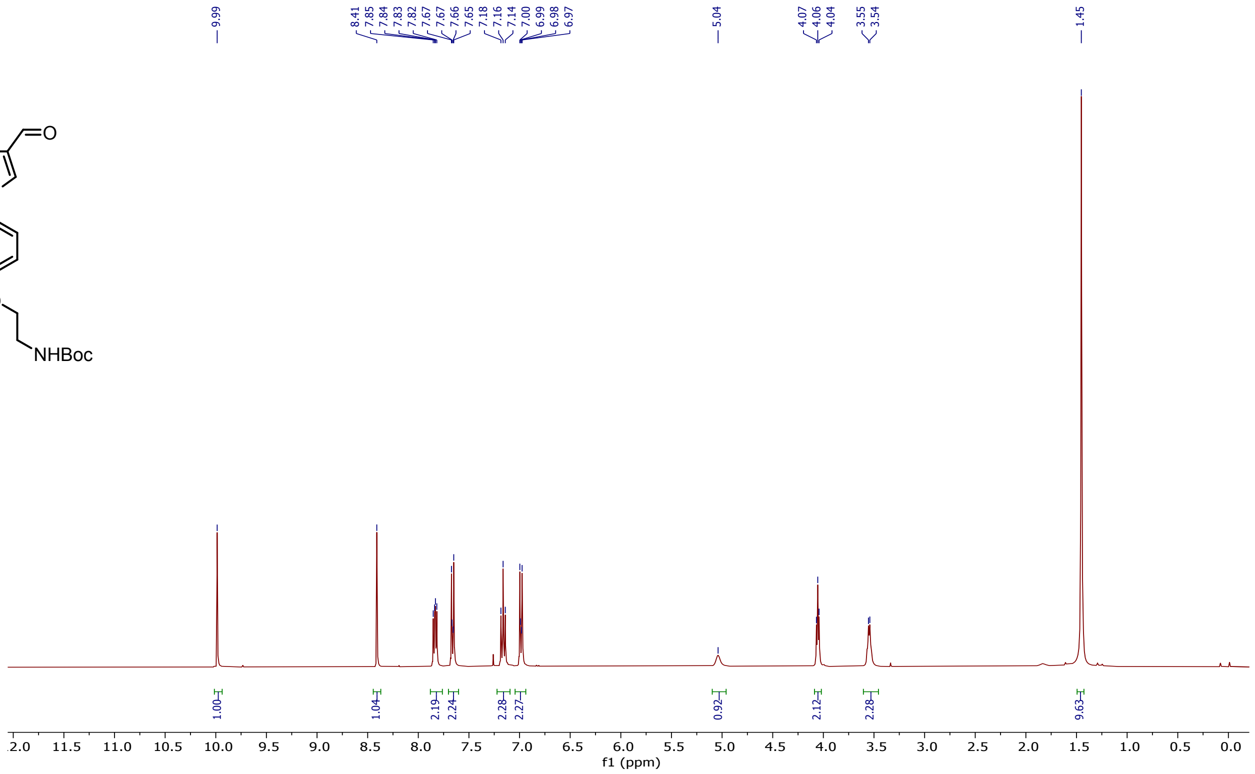

25c: ^{13}C NMR (101 MHz, $CDCl_3$):

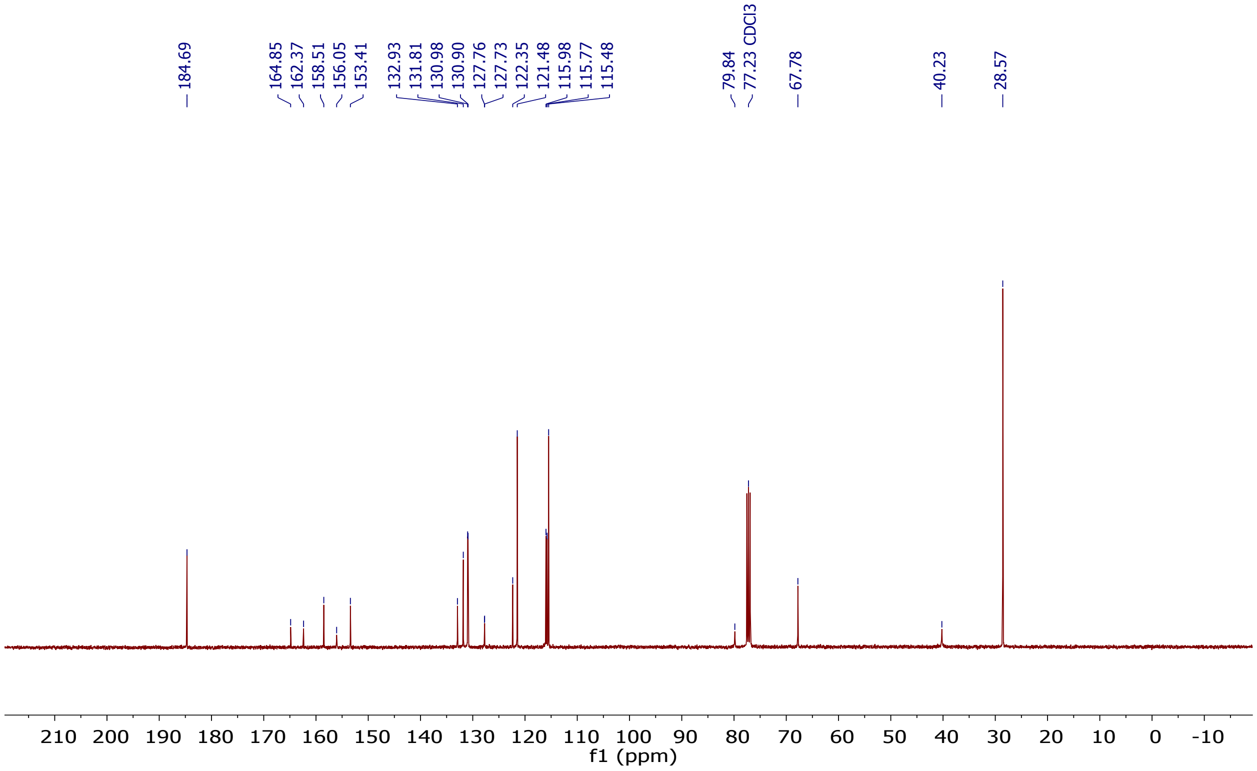

26a: ^{1}H NMR (400 MHz, $CDCl_3$):

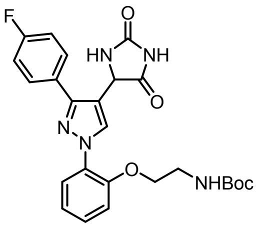

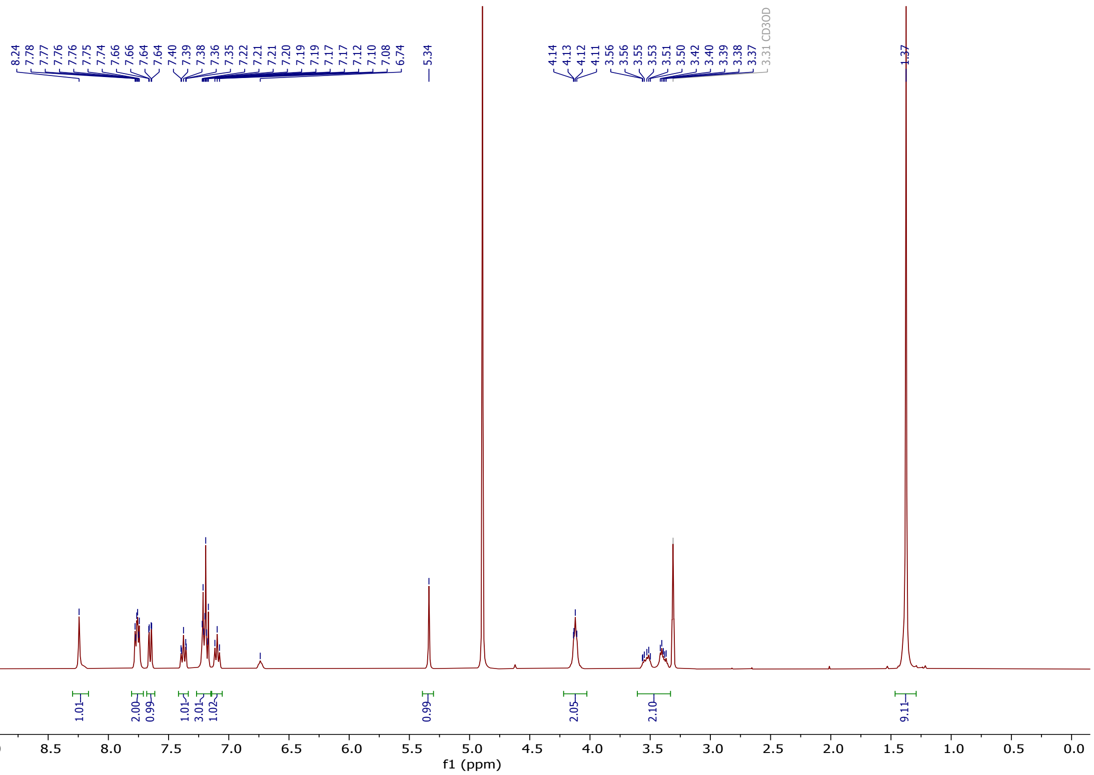

26a: ^{13}C NMR (101 MHz, $CDCl_3$):

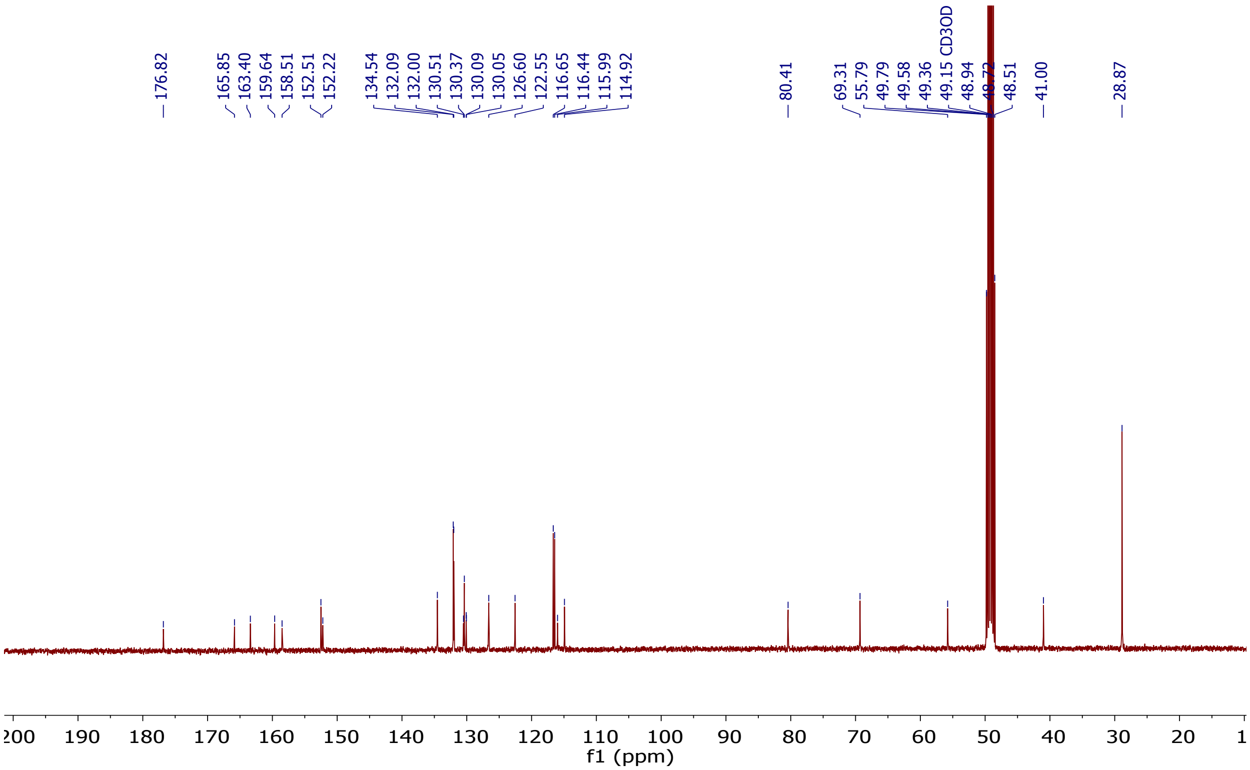

26b: ^{1}H NMR (400 MHz, $CDCl_3$):

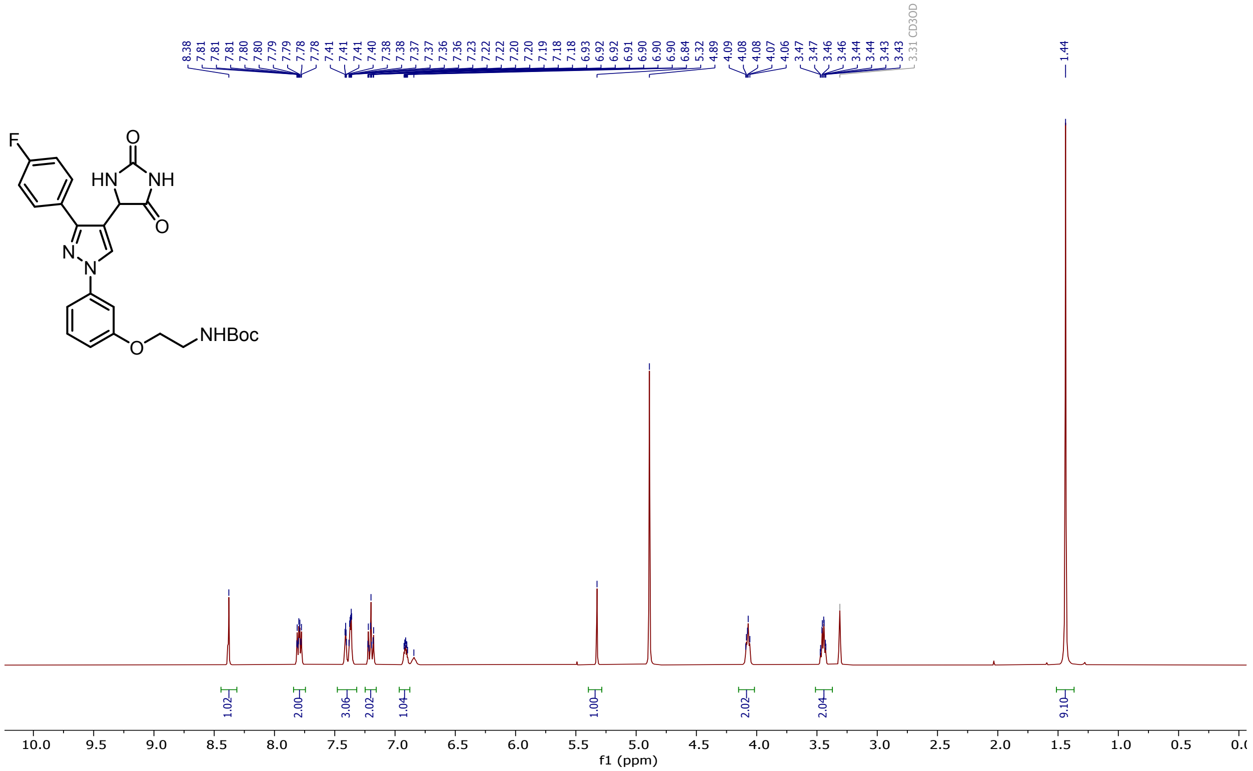

26b: ^{13}C NMR (101 MHz, $CDCl_3$):

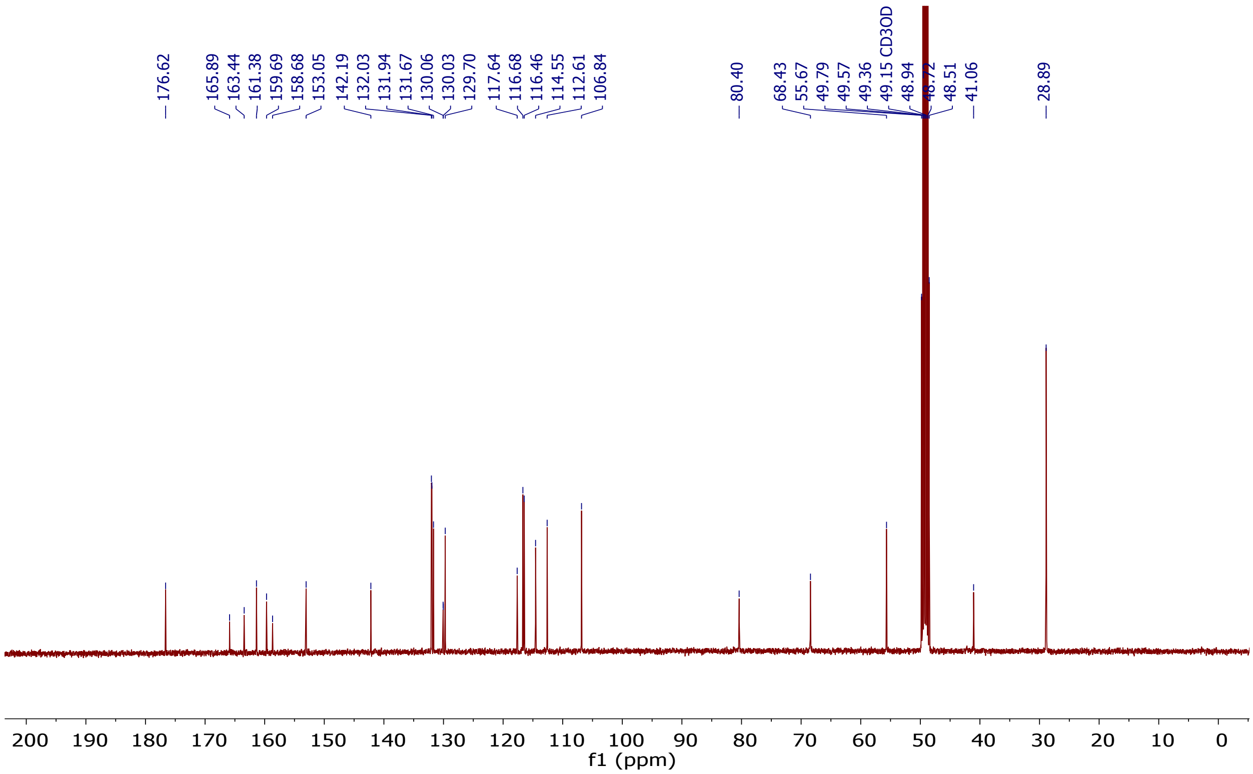

26c: ^{1}H NMR (400 MHz, $CDCl_3$):

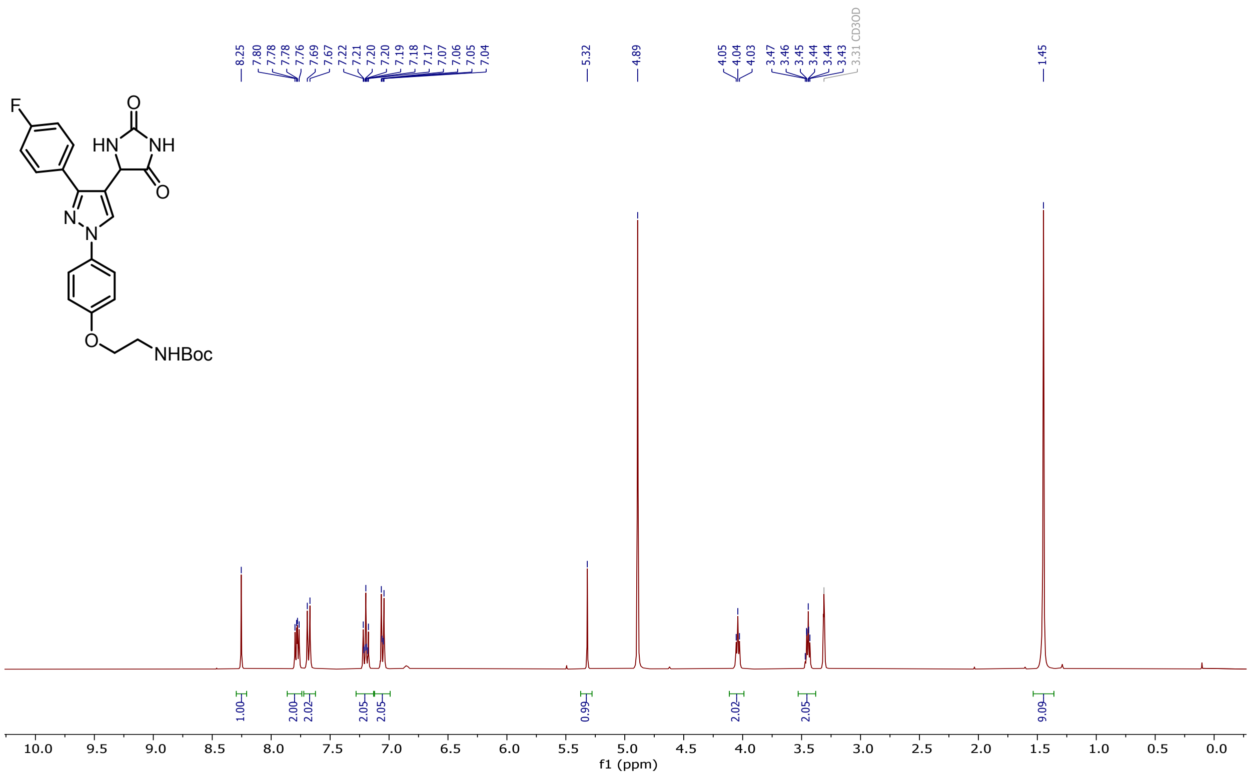

26c: ^{13}C NMR (101 MHz, $CDCl_3$):

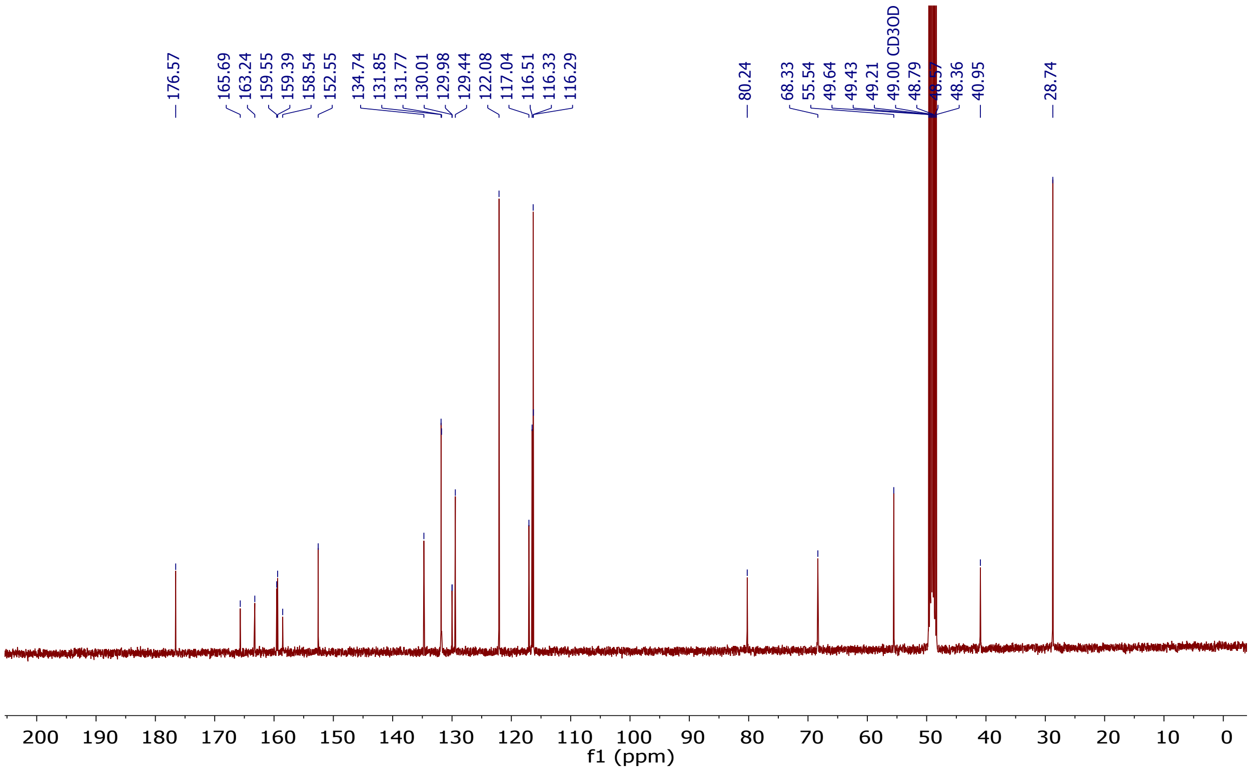

6: ^{1}H NMR (400 MHz, $CDCl_3$):

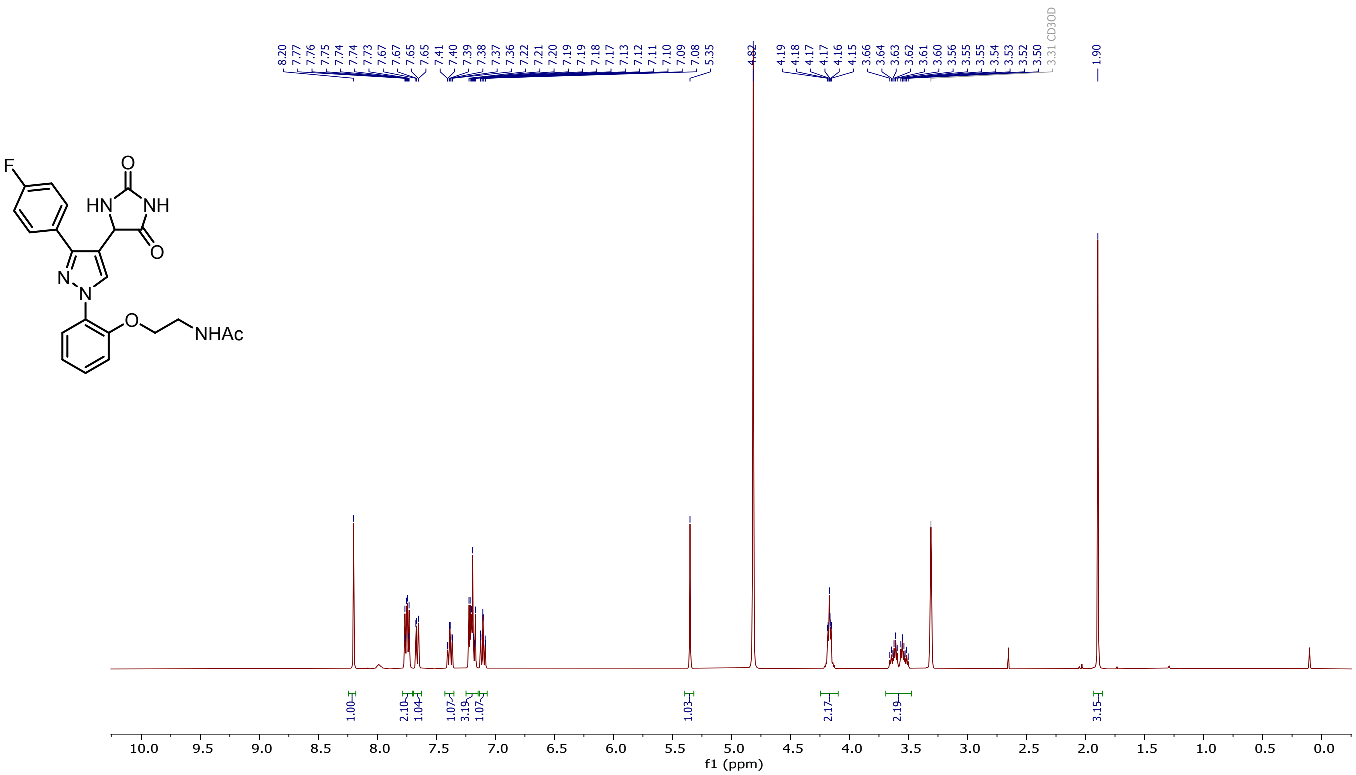

6: ^{13}C NMR (101 MHz, $CDCl_3$):

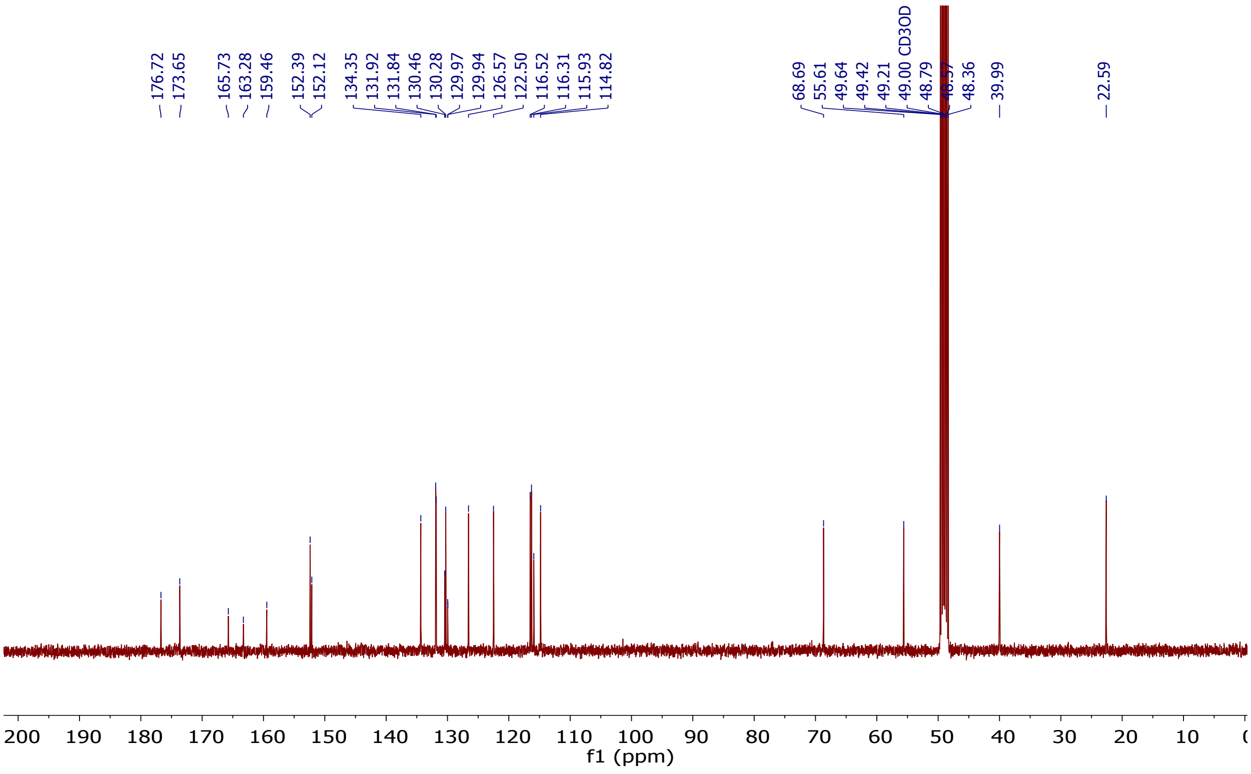

8: ^{1}H NMR (400 MHz, $CDCl_3$):

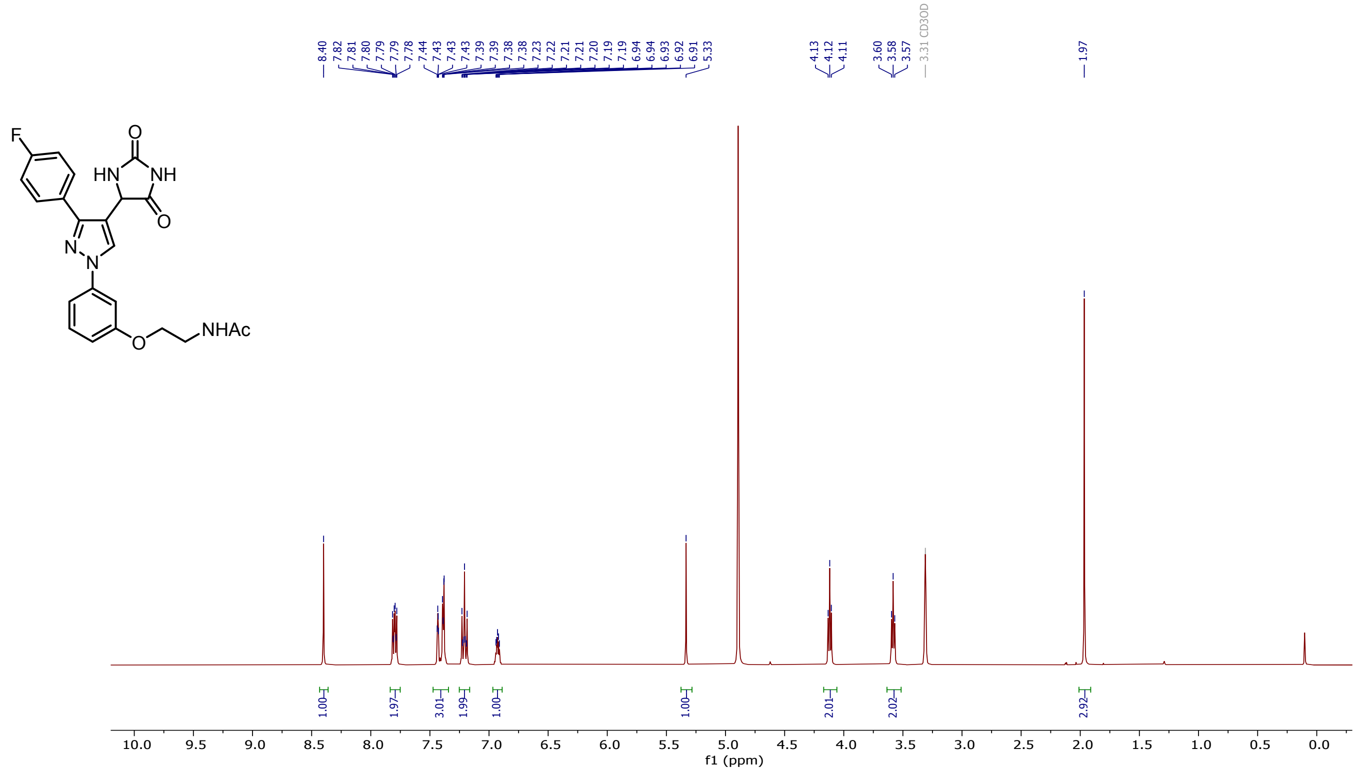

8: ^{13}C NMR (101 MHz, $CDCl_3$):

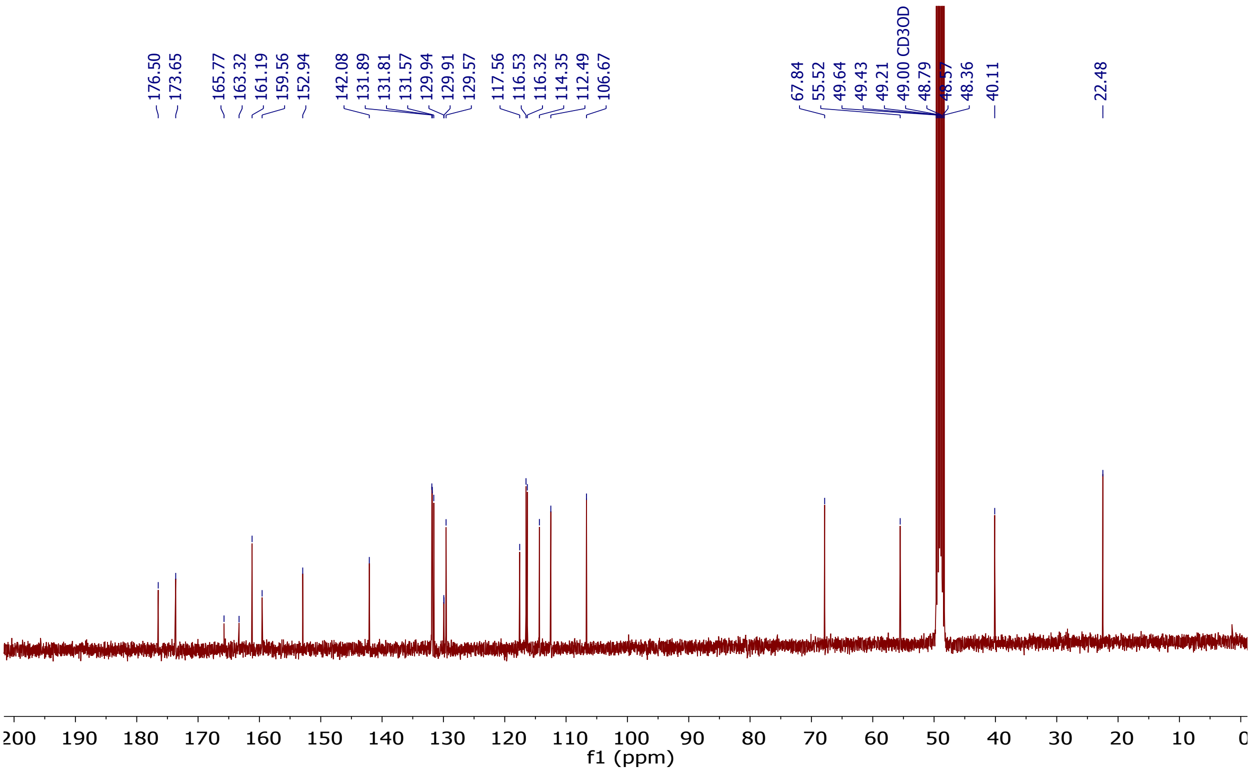

10: ^{1}H NMR (400 MHz, $CDCl_3$):

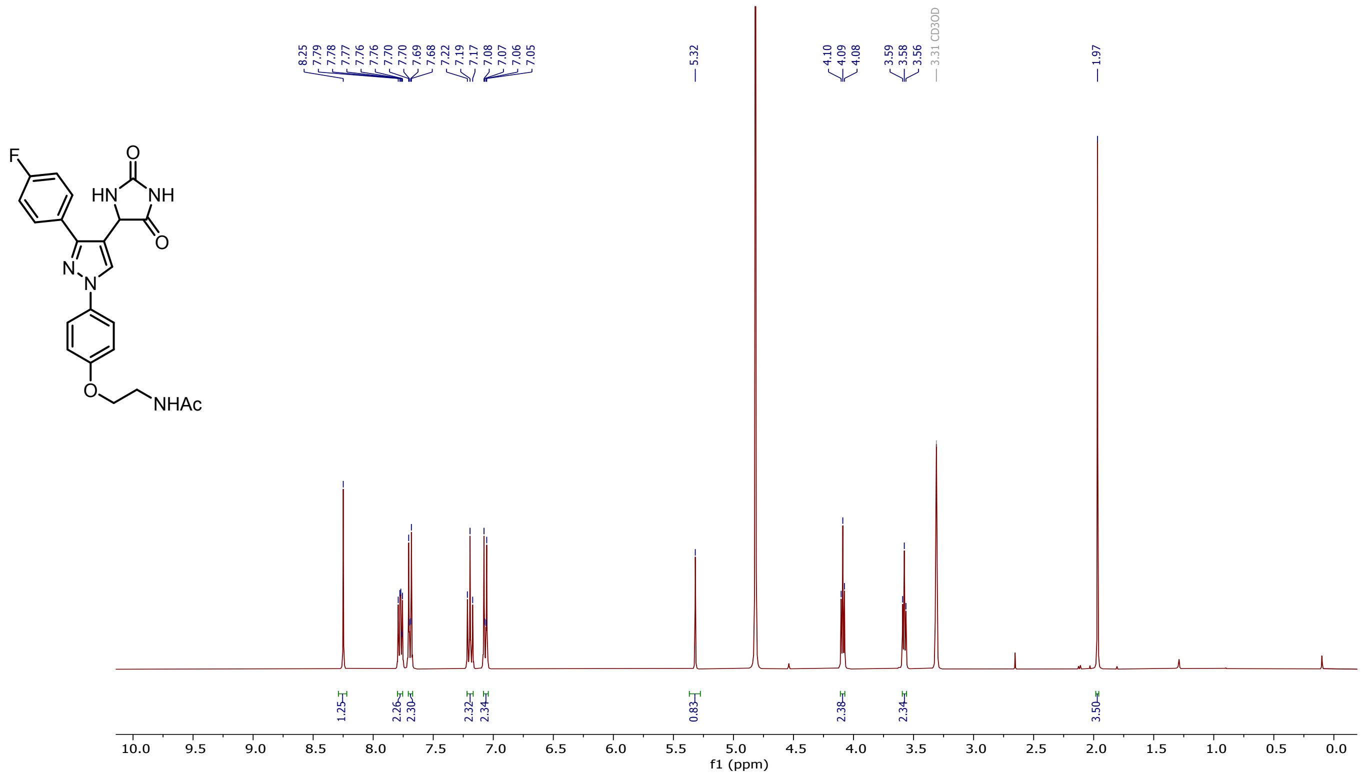

10: ^{13}C NMR (101 MHz, $CDCl_3$):

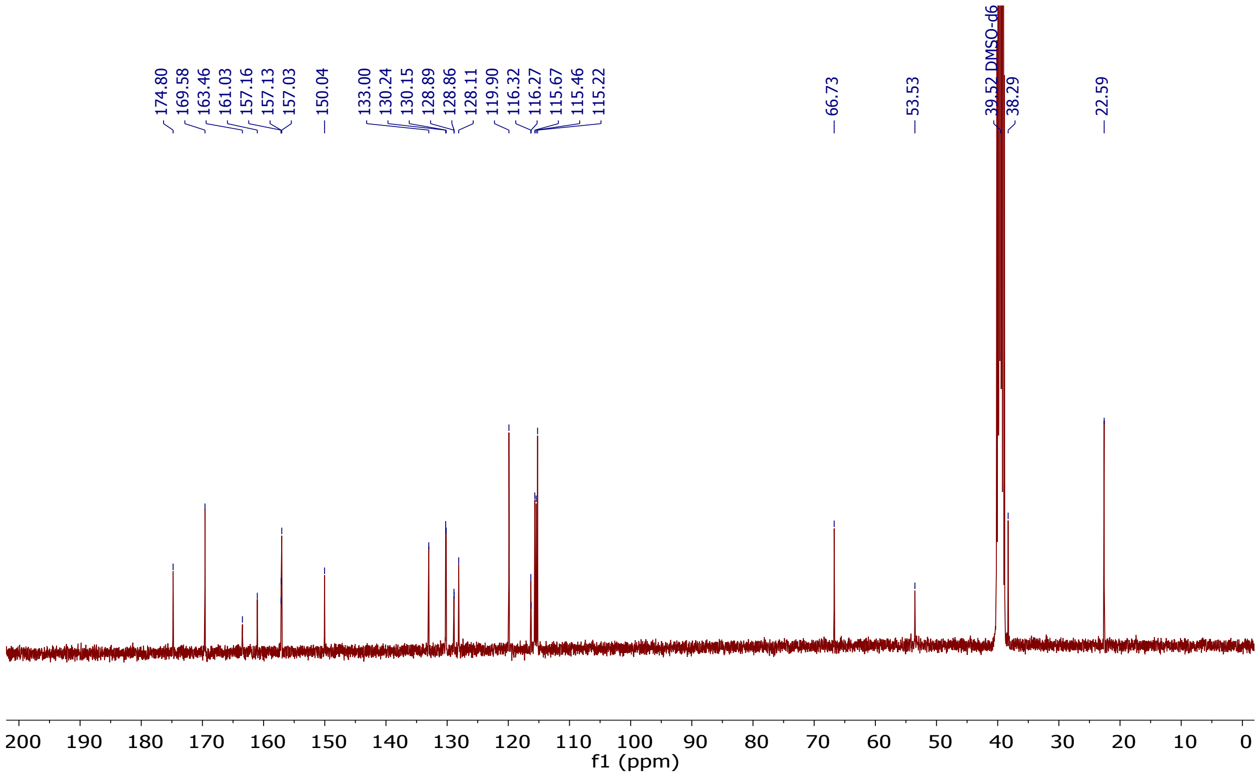

7: ^{1}H NMR (400 MHz, $CDCl_3$):

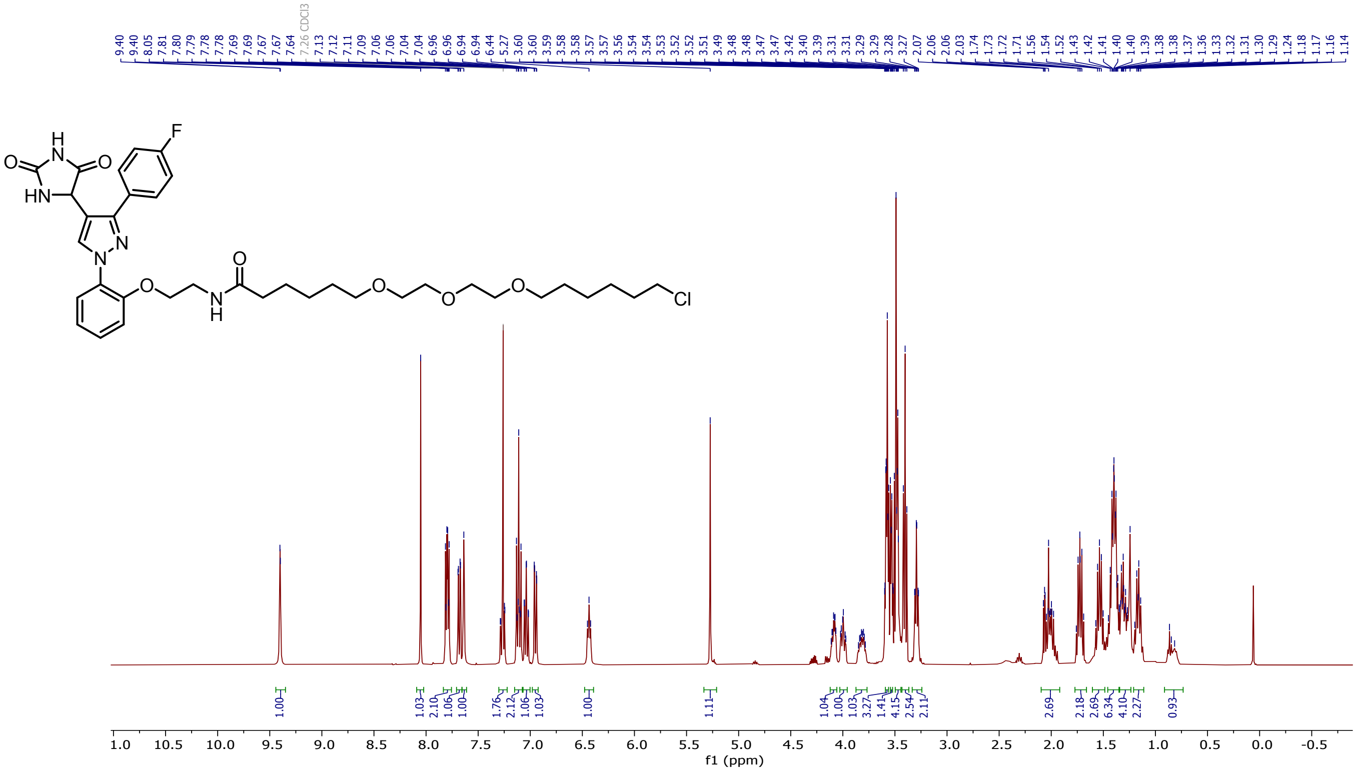

7: ^{13}C NMR (101 MHz, $CDCl_3$):

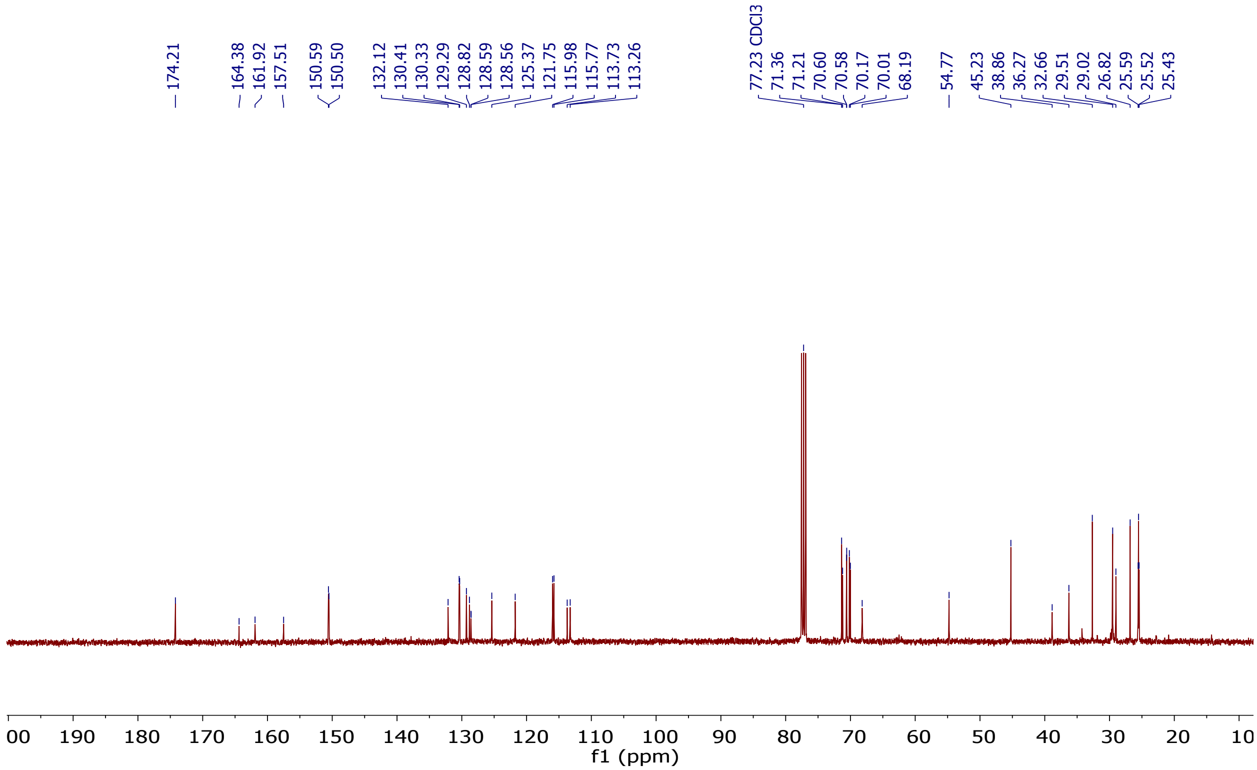

7: ^{19}F NMR (376 MHz, $CDCl_3$):

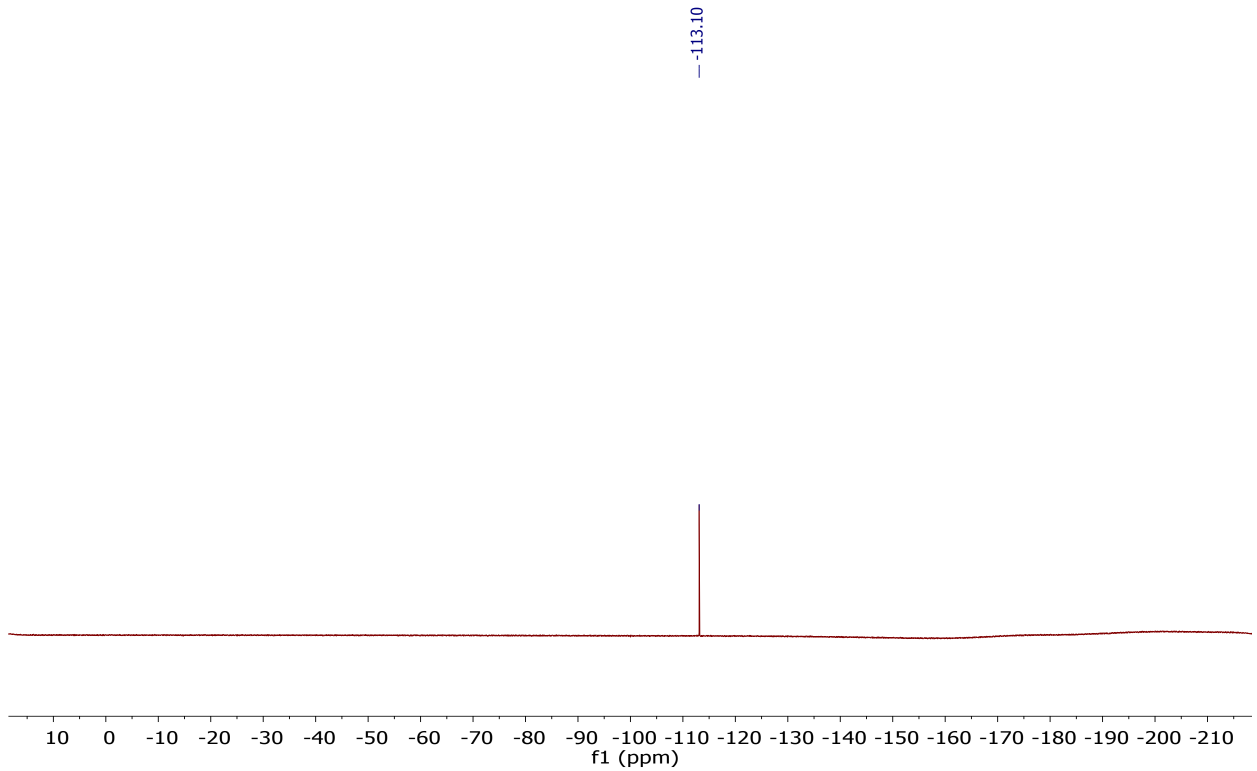

9: ^{1}H NMR (400 MHz, $CDCl_3$):

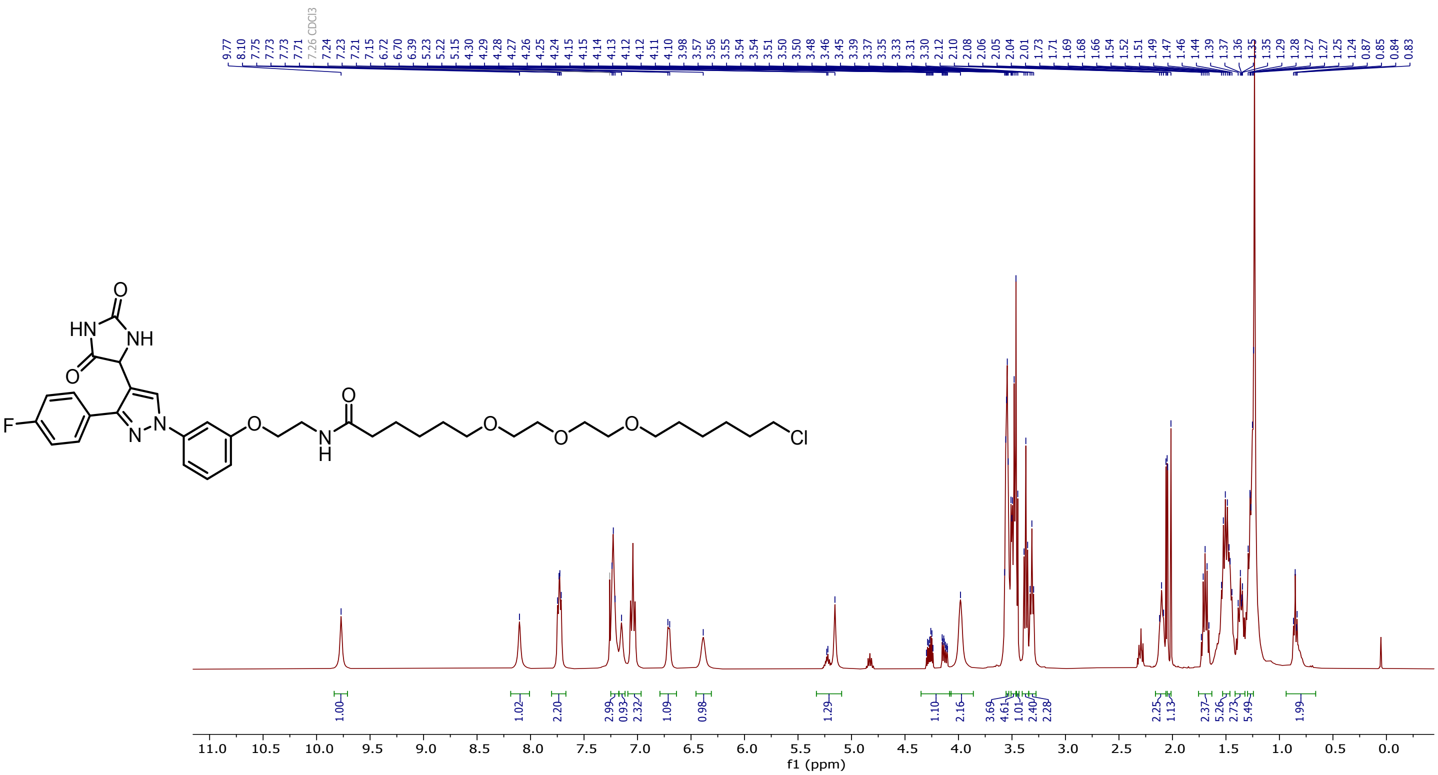

9: ^{13}C NMR (101 MHz, $CDCl_3$):

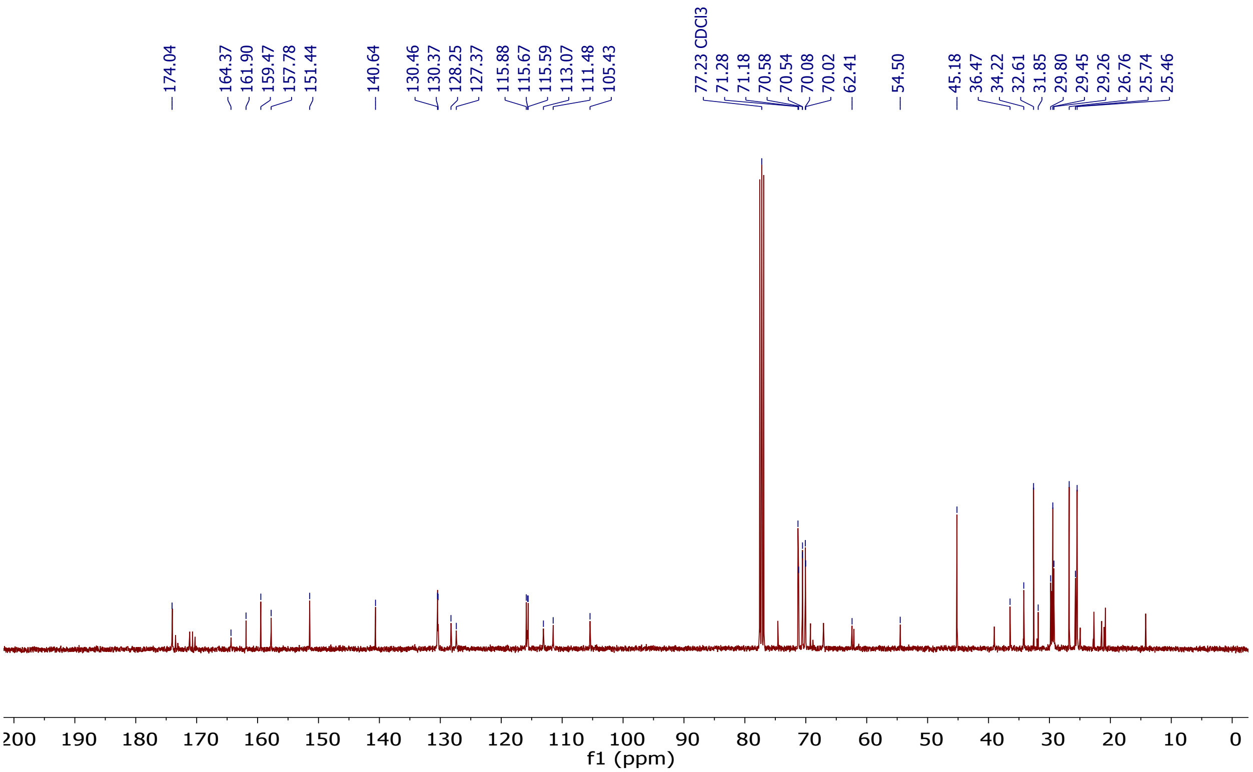

9: ^{19}F NMR (376 MHz, $CDCl_3$):

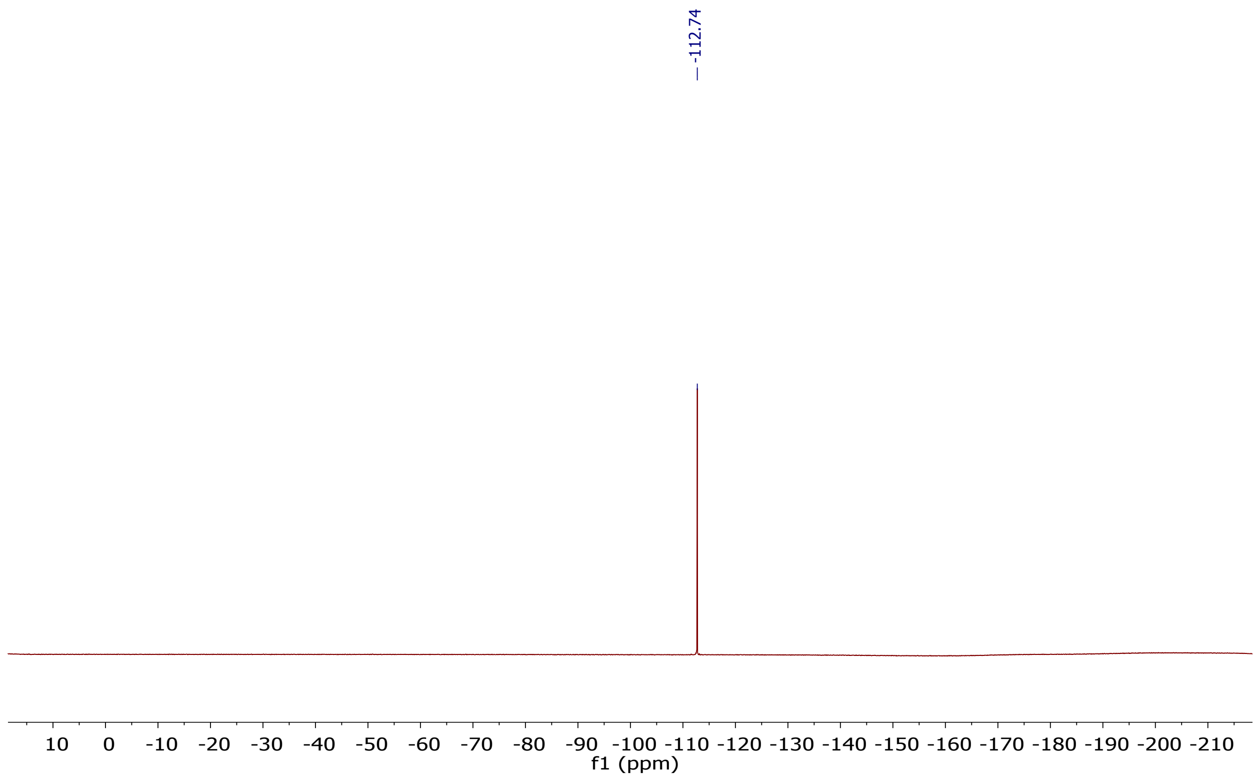

11: ^{1}H NMR (400 MHz, $CDCl_3$):

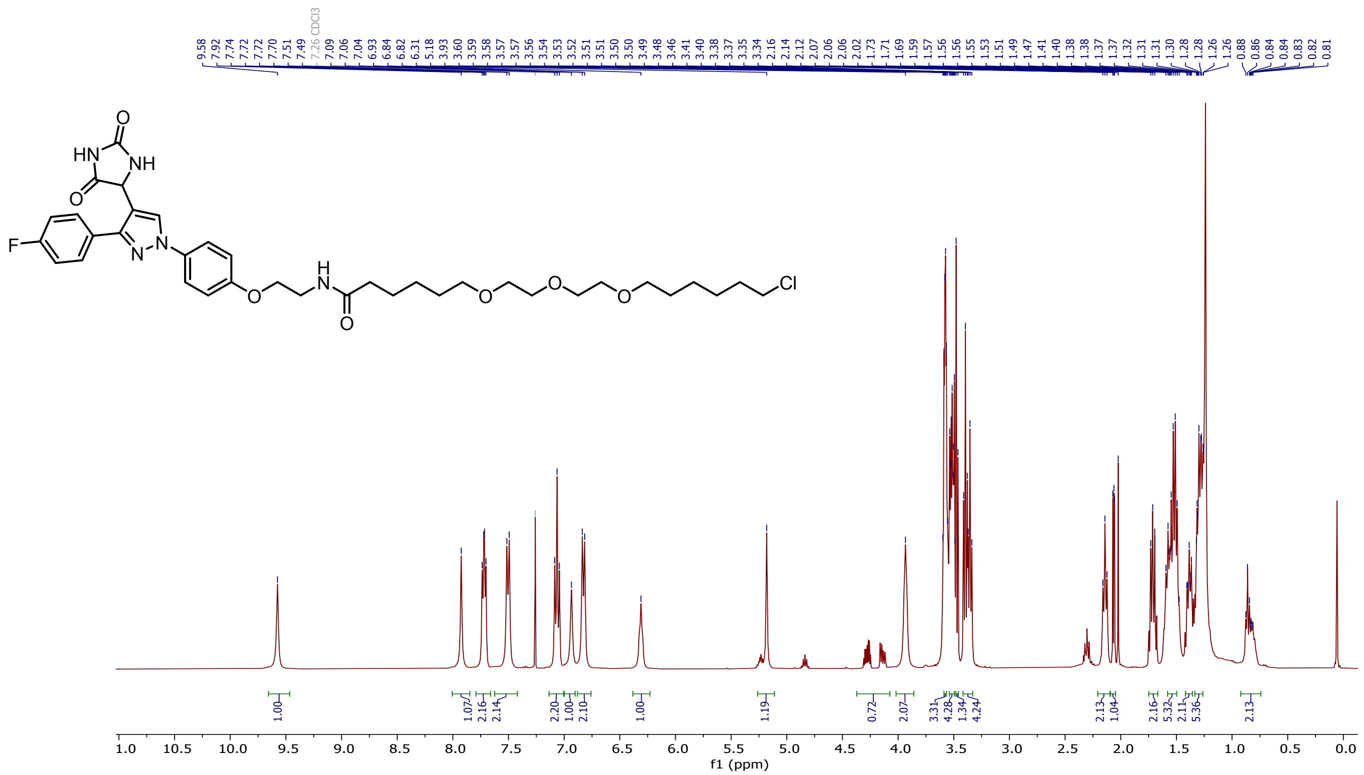

11: ^{13}C NMR (101 MHz, $CDCl_3$

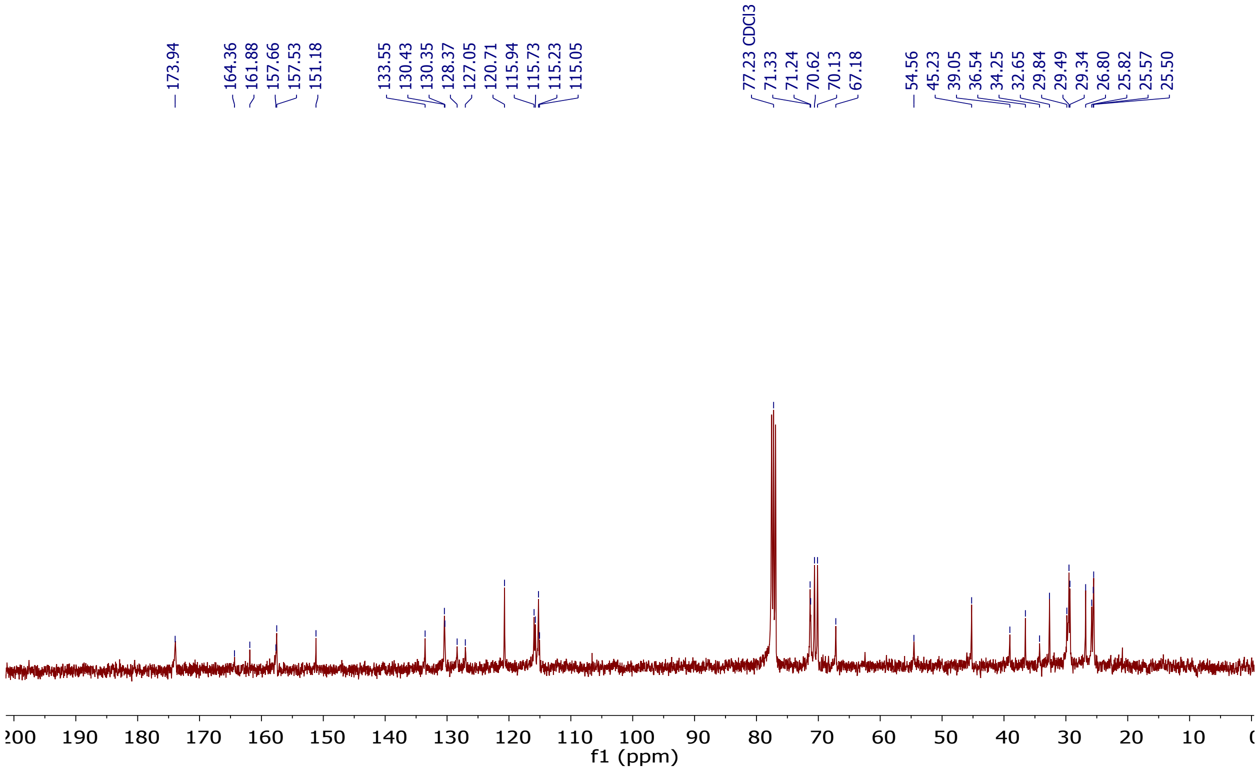

11: ^{19}F NMR (376 MHz, $CDCl_3$):

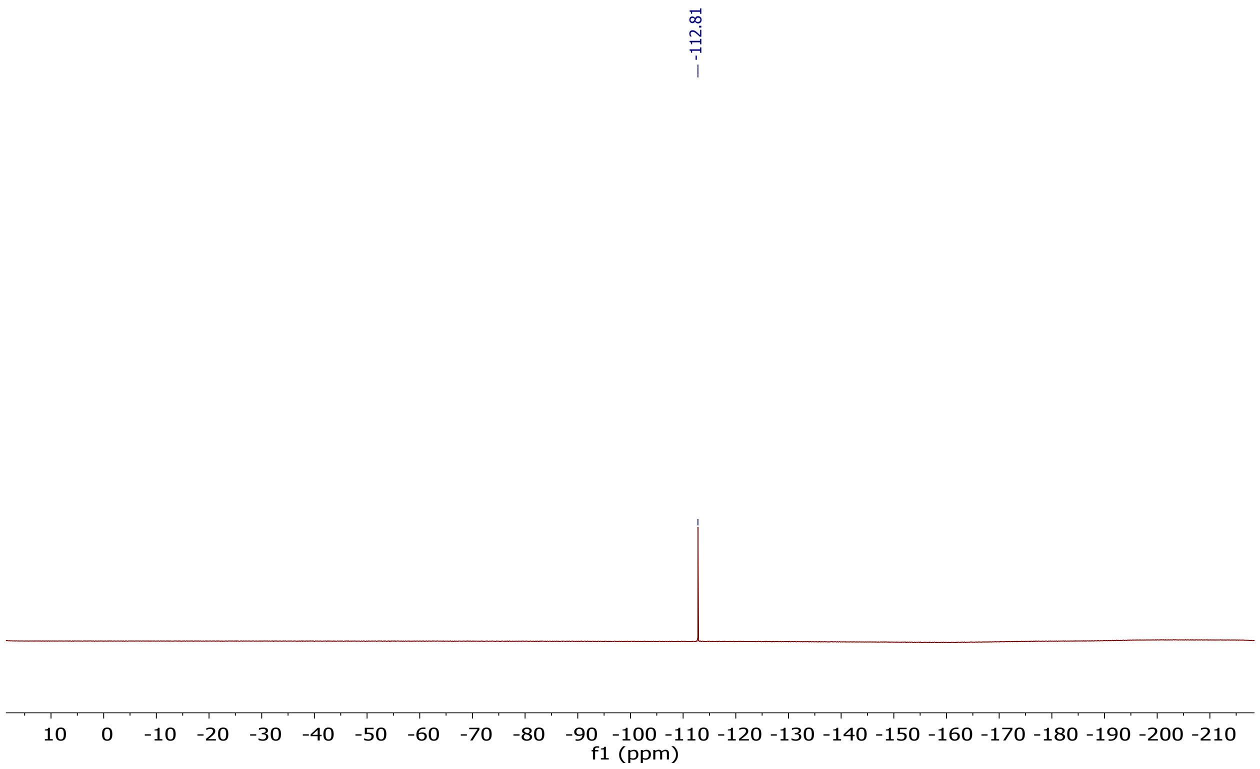

12: ^{1}H NMR (400 MHz, $CDCl_3$):

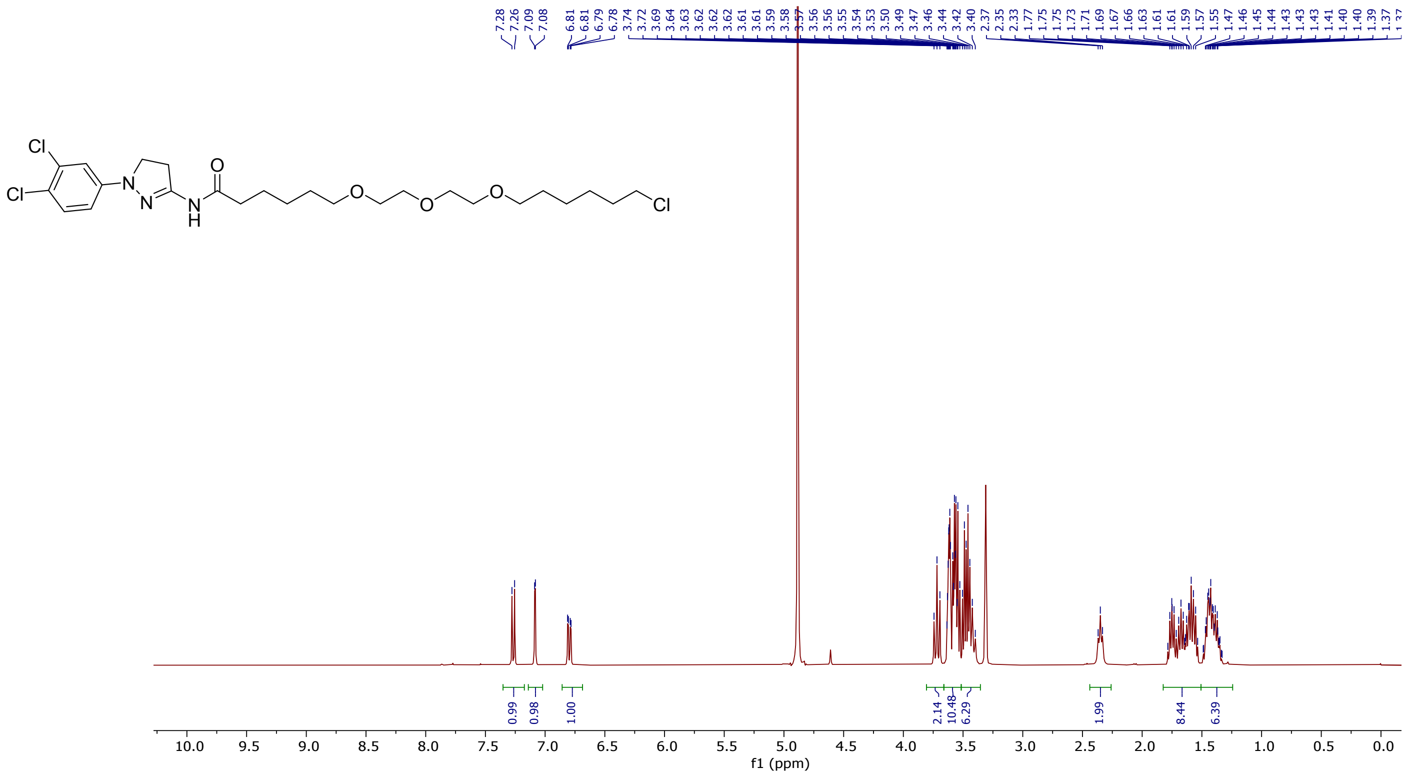

12: ^{13}C NMR (101 MHz, $CDCl_3$):

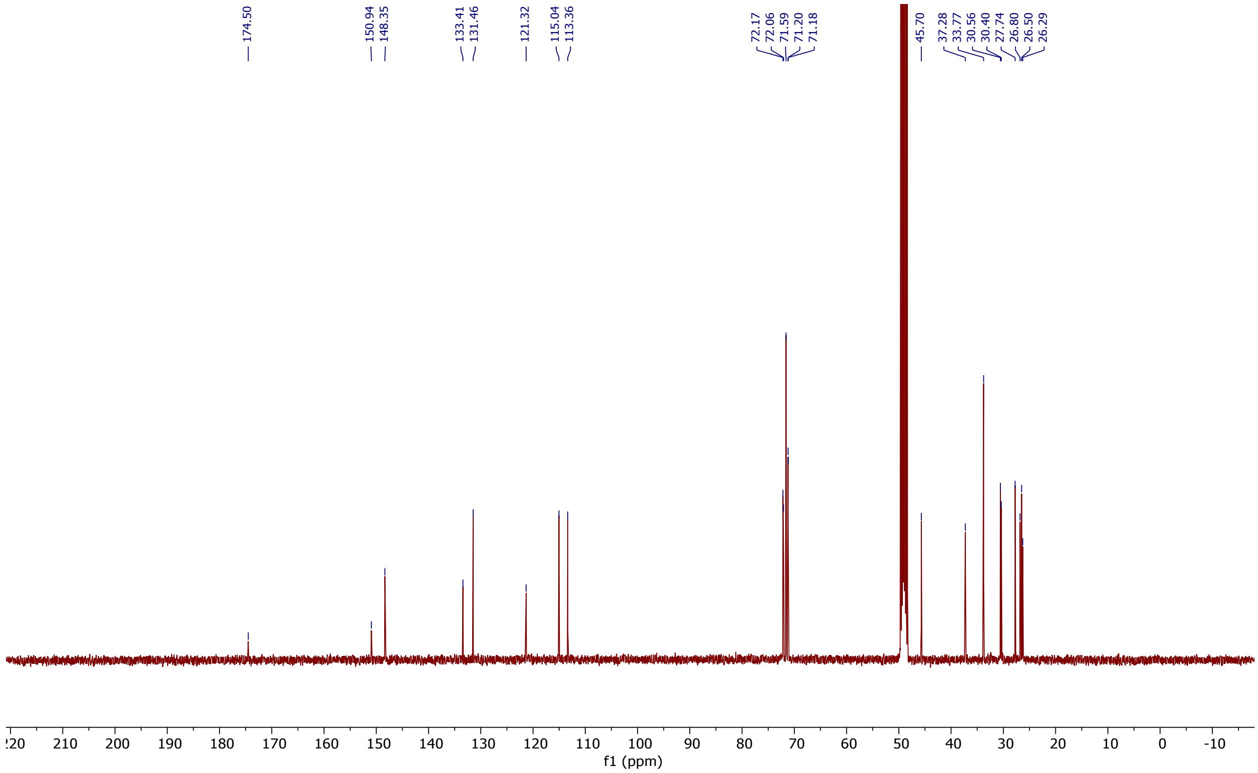

41: ^{1}H NMR (400 MHz, $CDCl_3$):

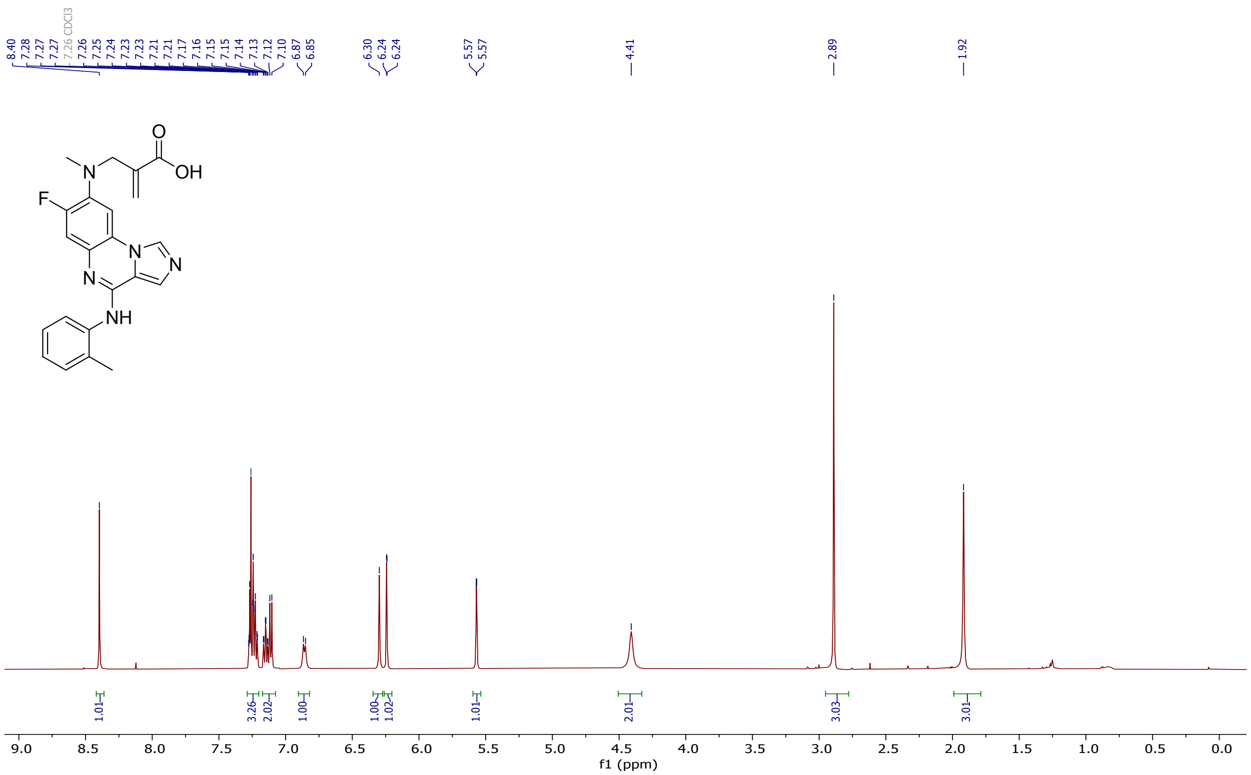

41: ^{13}C NMR (126 MHz, $CDCl_3$):

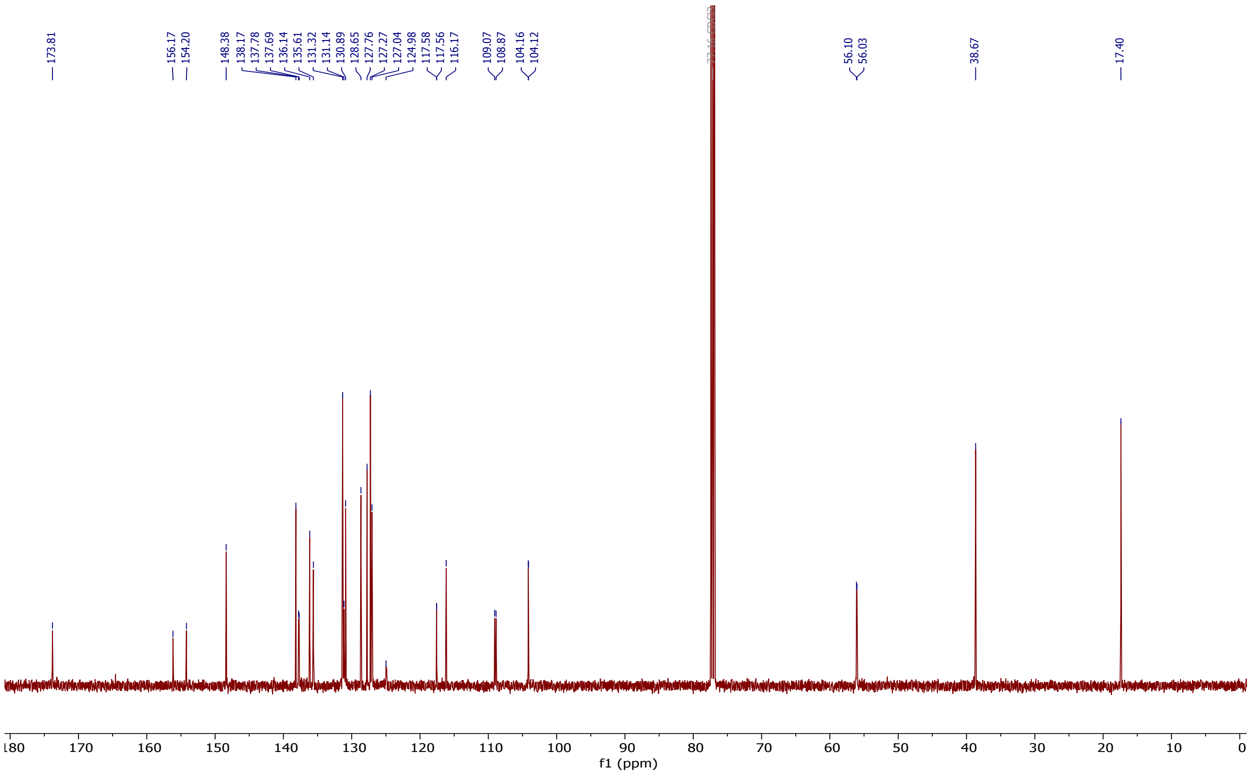

15: ^{1}H NMR (500 MHz, $CDCl_3$):

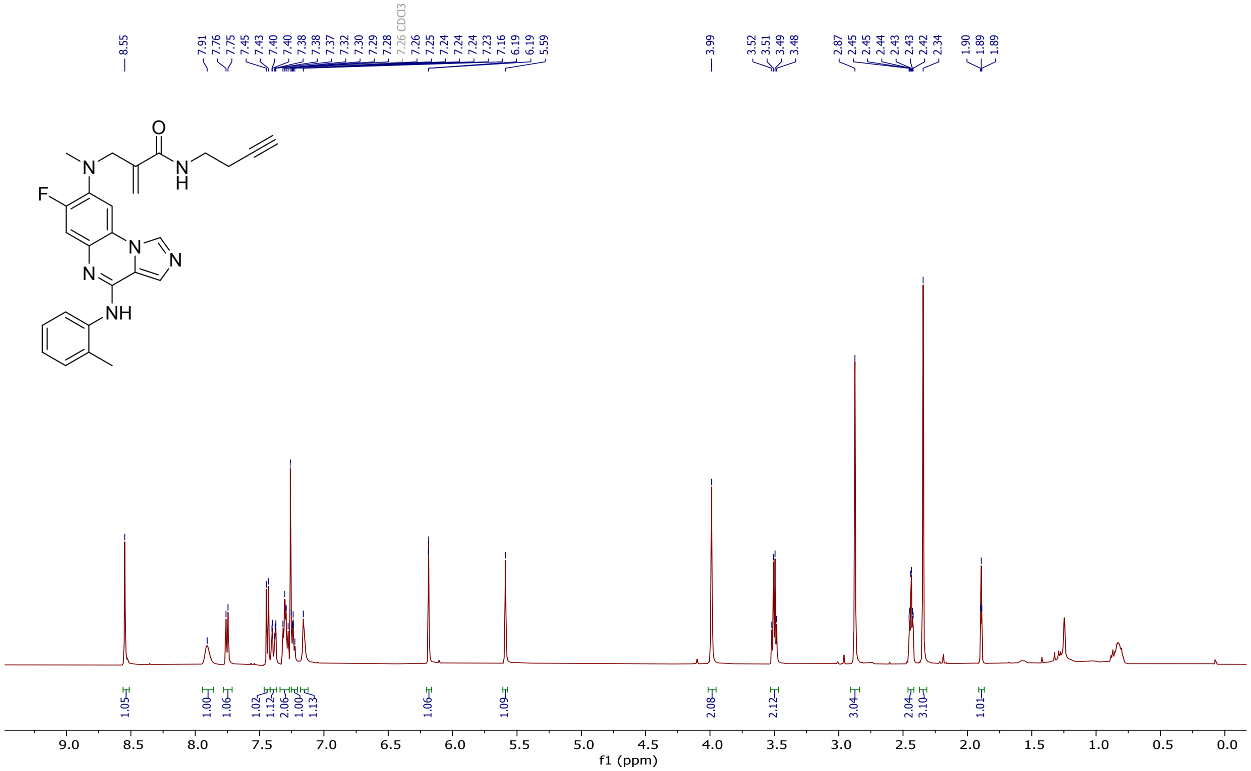

15: ^{13}C NMR (126 MHz, $CDCl_3$):

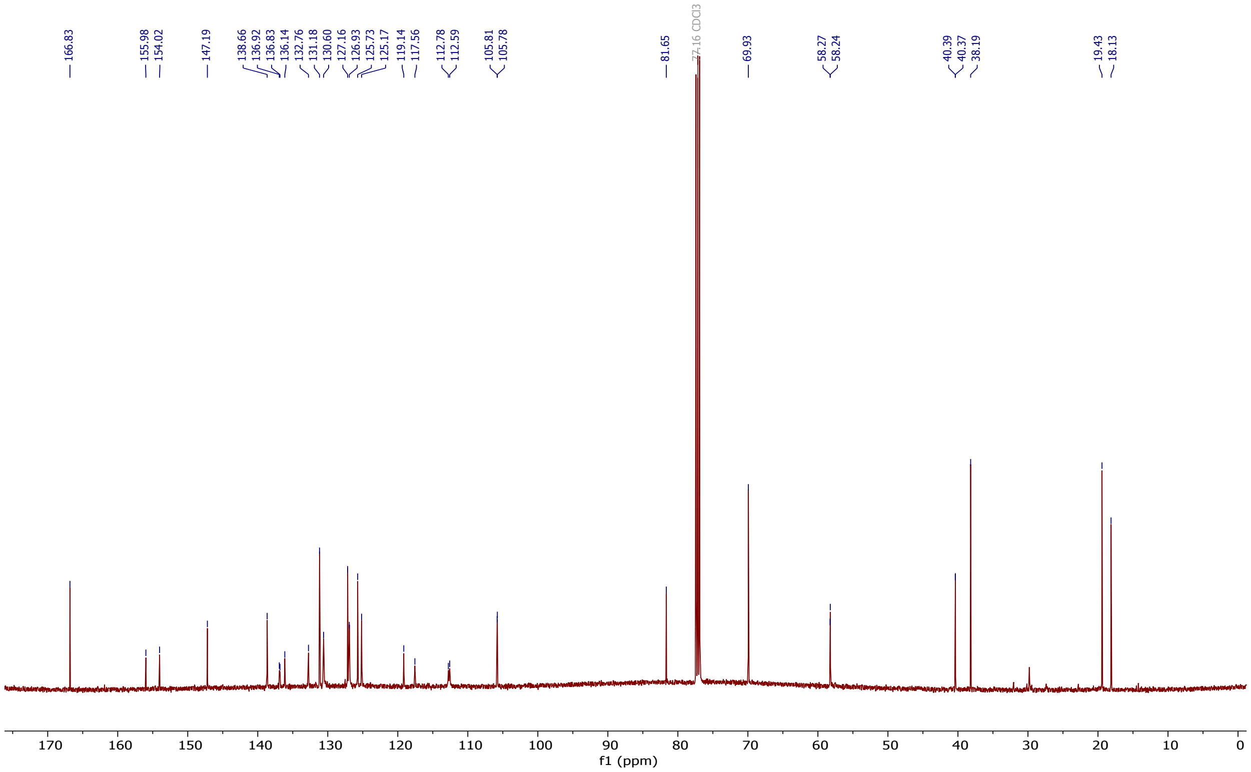

17: ^{1}H NMR (500 MHz, $CDCl_3$):

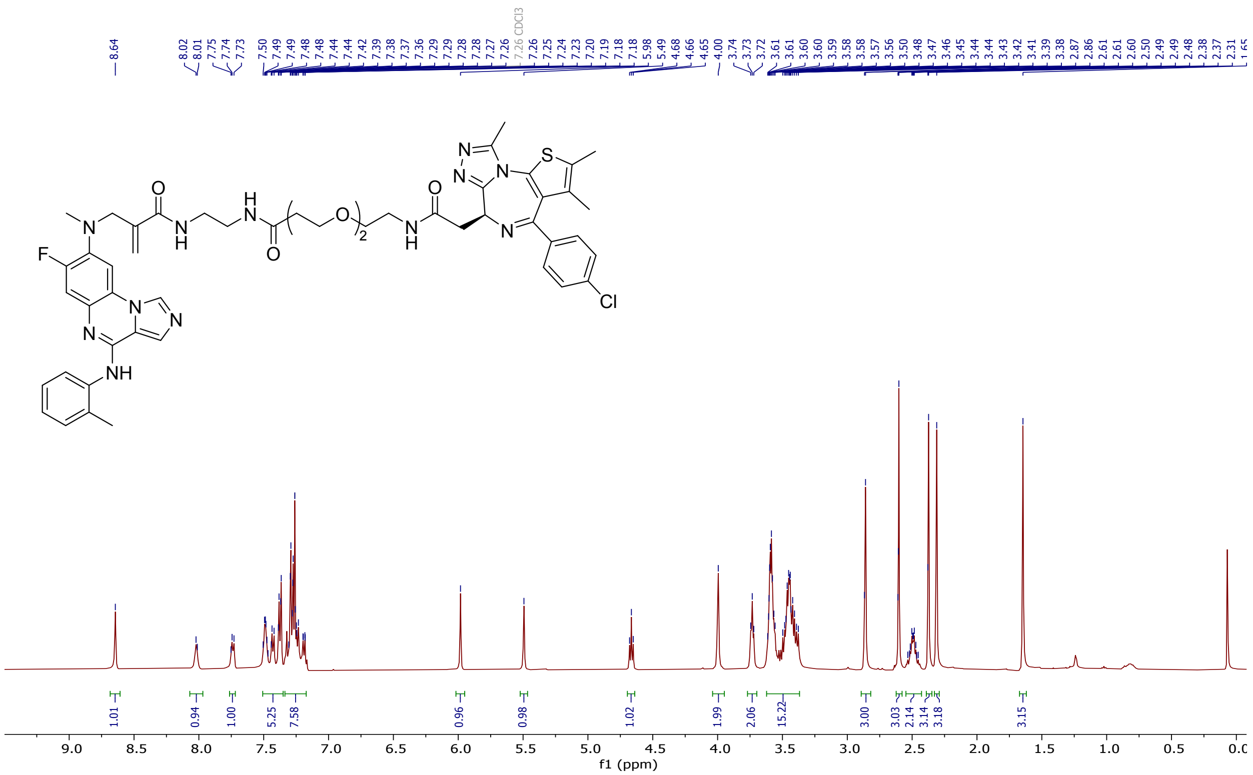

17: ^{13}C NMR (126 MHz, $CDCl_3$):

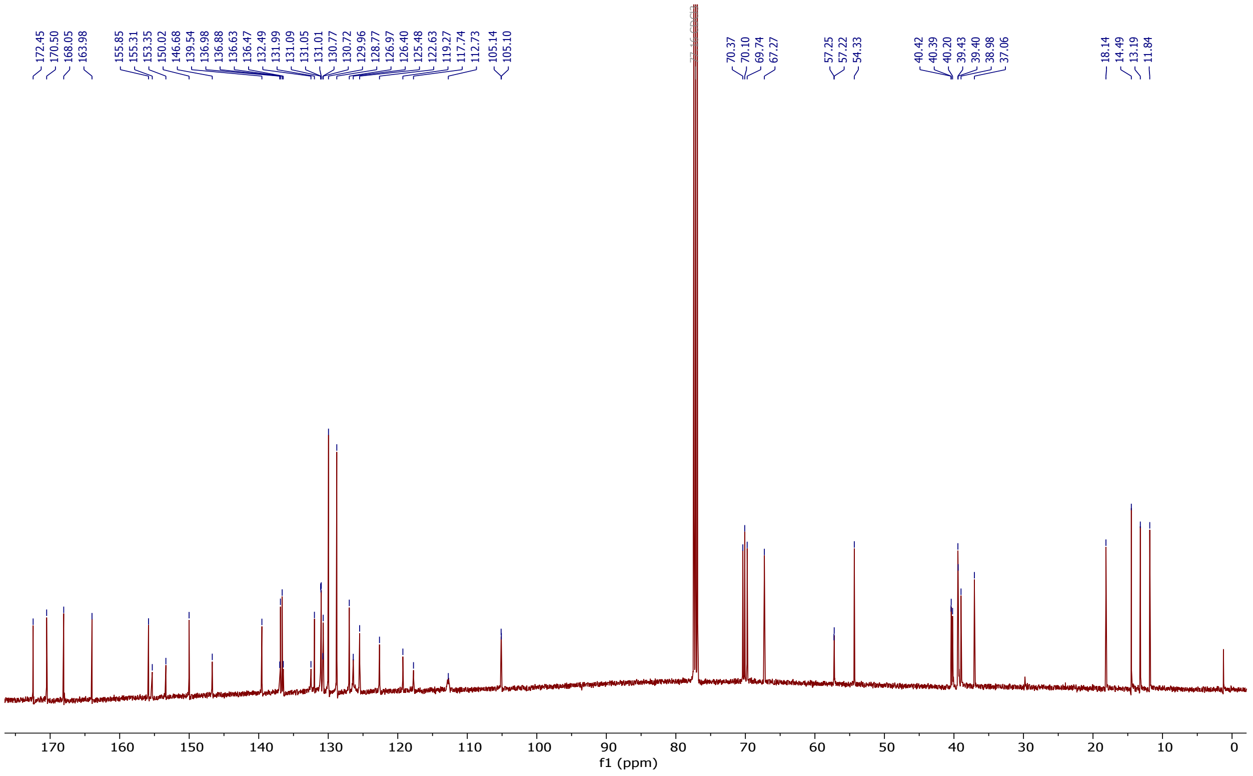

19: ^{1}H NMR (400 MHz, $CDCl_3$):

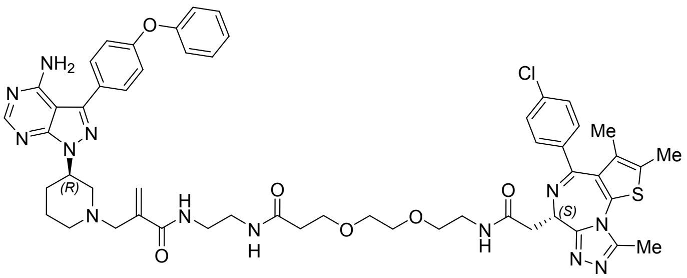

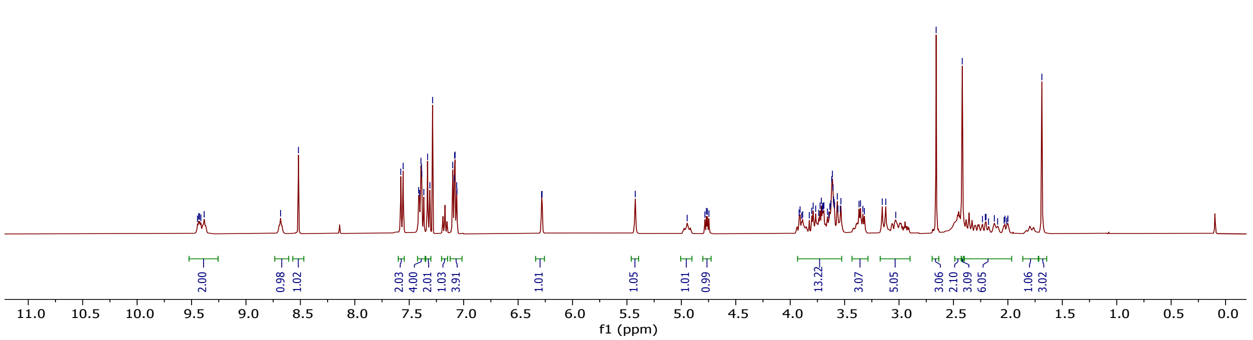

19: ^{13}C NMR (101 MHz, $CDCl_3$):

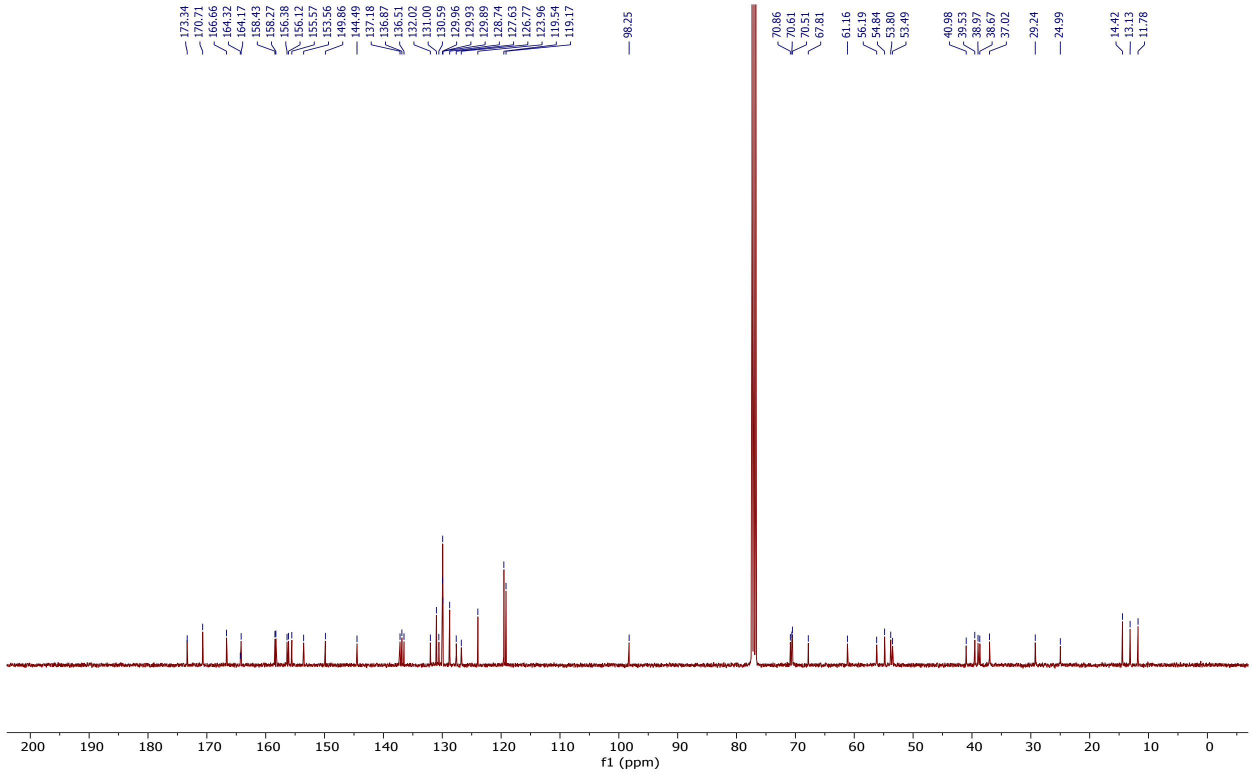

20: ^{1}H NMR (400 MHz, $CDCl_3$):

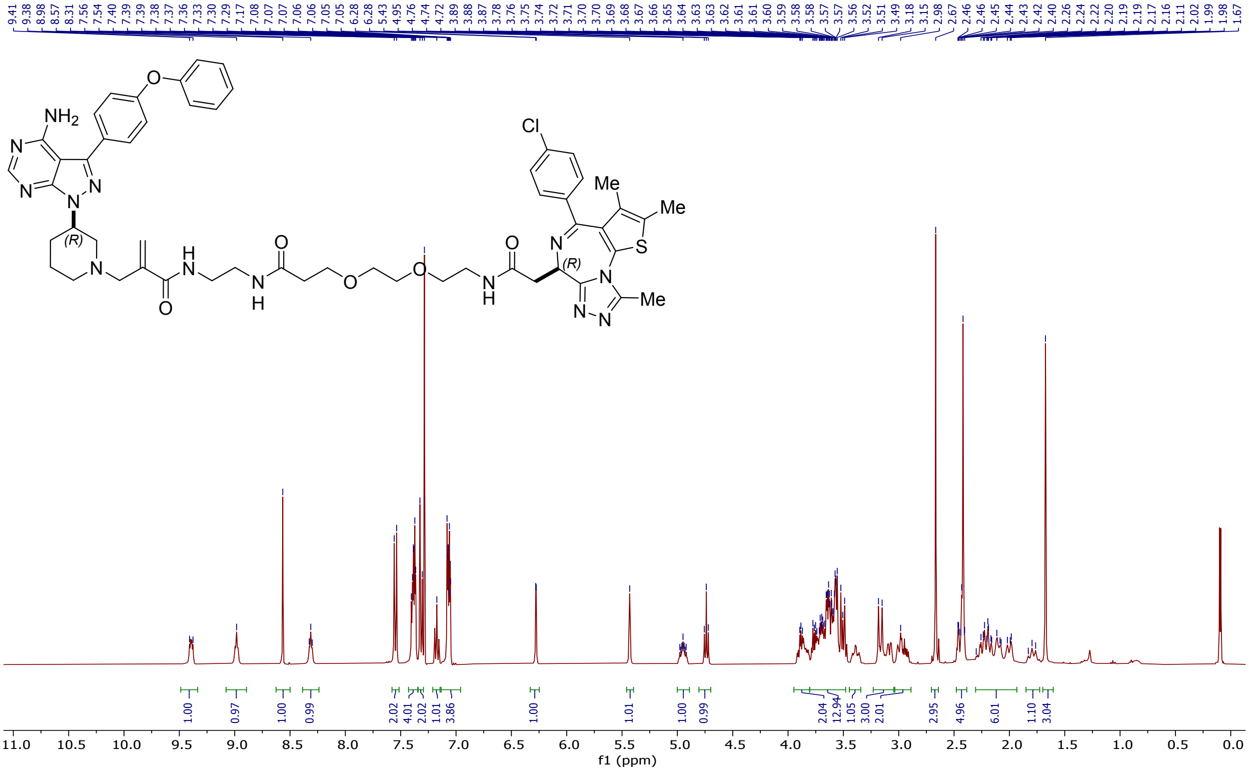

20: ^{13}C NMR (101 MHz, $CDCl_3$):

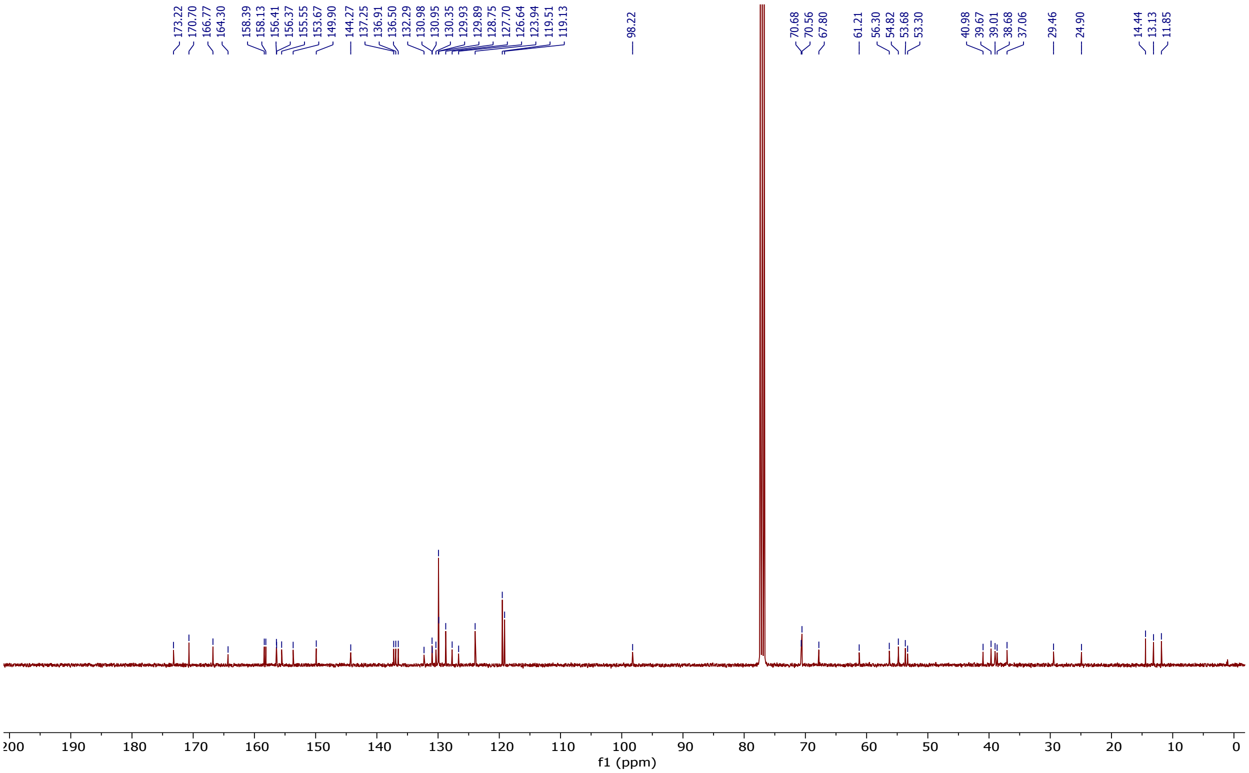

21: ^{1}H NMR (400 MHz, $CDCl_3$):

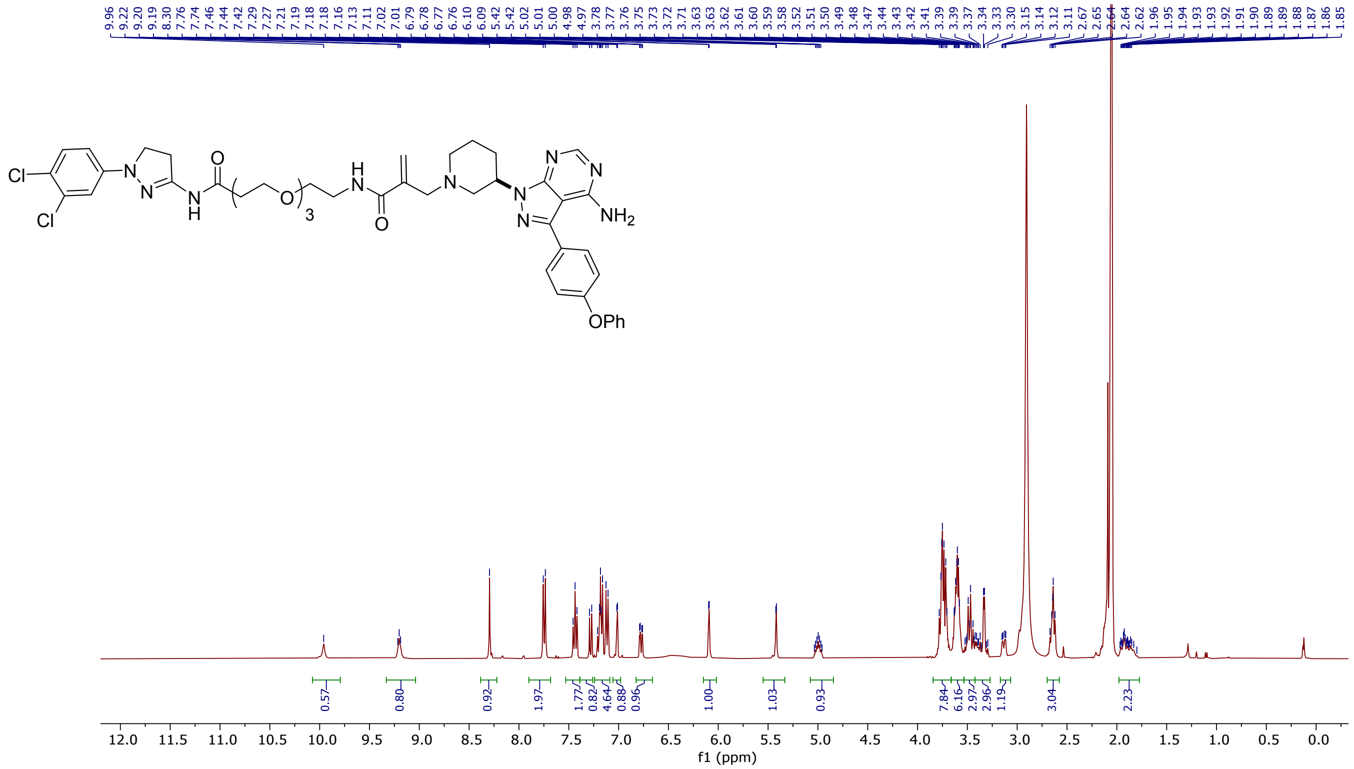

21: ^{13}C NMR (101 MHz, $CDCl_3$):

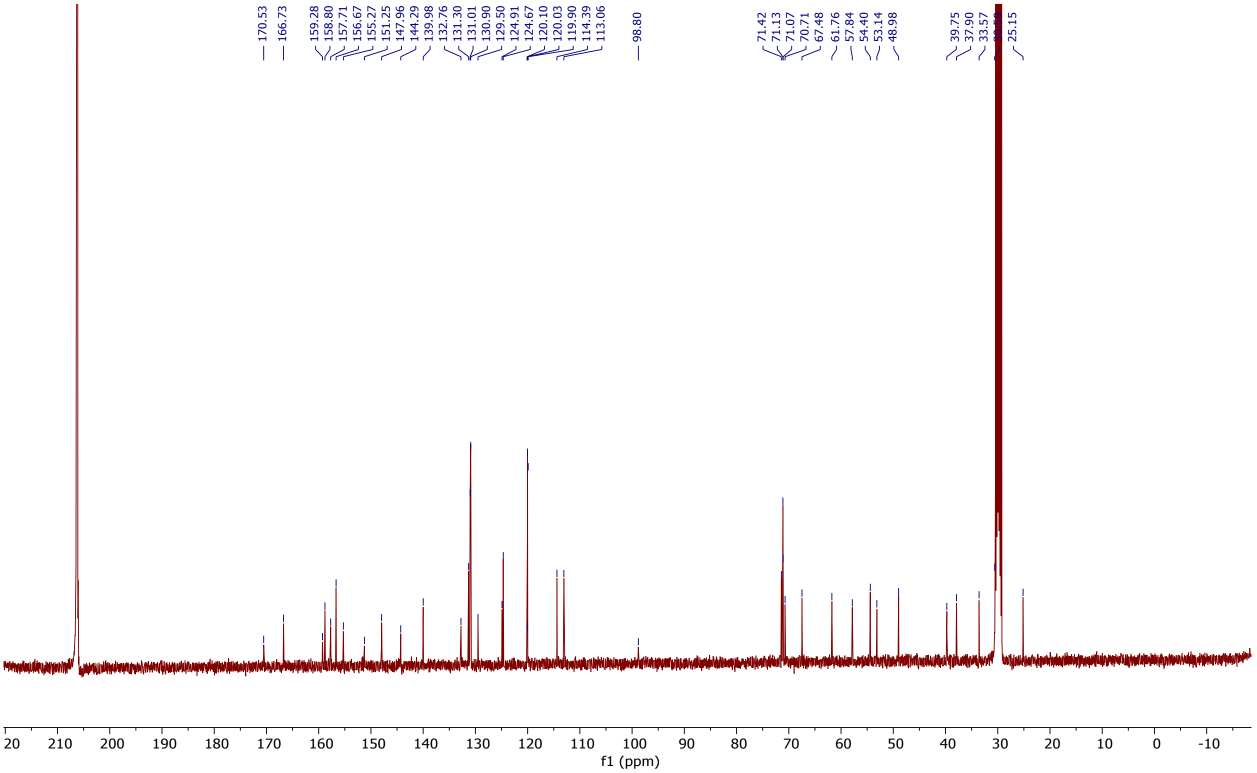

22: ^{1}H NMR (400 MHz, $CDCl_3$):

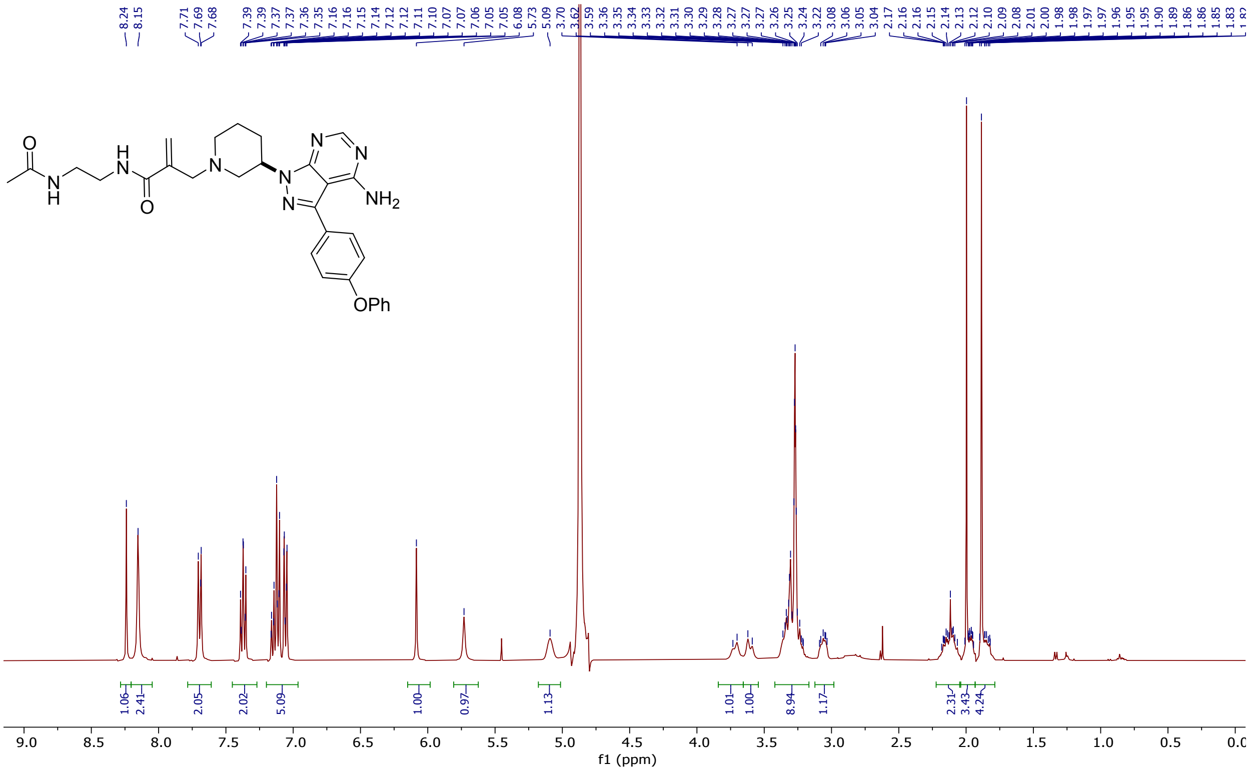

22: ^{13}C NMR (101 MHz, $CDCl_3$):

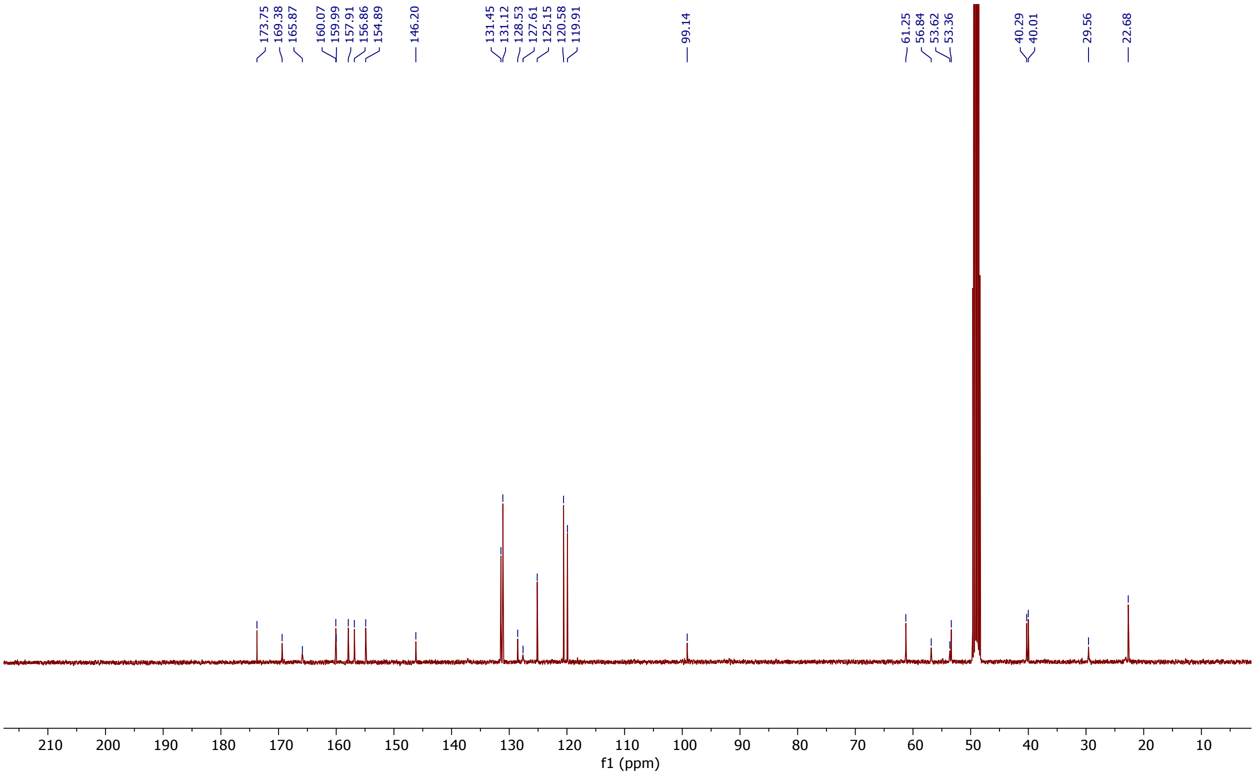

2. References.

(1) Simpson, G. L.; Bertrand, S. M.; Borthwick, J. A.; Campobasso, N.; Chabanet, J.; Chen, S.; Coggins, J.; Cottom, J.; Christensen, S. B.; Dawson, H. C.; et al. Identification and Optimization of Novel Small c-Abl Kinase Activators Using Fragment and HTS Methodologies. *J Med Chem* **2019**, *62* (4), 2154-2171.

(2) Kim, K. H.; Maderna, A.; Schnute, M. E.; Hegen, M.; Mohan, S.; Miyashiro, J.; Lin, L.; Li, E.; Keegan, S.; Lussier, J.; et al. Imidazo[1,5-a]quinoxalines as irreversible BTK inhibitors for the treatment of rheumatoid arthritis. *Bioorg Med Chem Lett* **2011**, *21* (21), 6258-6263.

(3) Wei, J.; Meng, F.; Park, K. S.; Yim, H.; Velez, J.; Kumar, P.; Wang, L.; Xie, L.; Chen, H.; Shen, Y.; et al. Harnessing the E3 Ligase KEAP1 for Targeted Protein Degradation. *J Am Chem Soc* **2021**, *143* (37), 15073-15083.

(4) Reddi, R. N.; Rogel, A.; Resnick, E.; Gabizon, R.; Prasad, P. K.; Gurwicz, N.; Barr, H.; Shulman, Z.; London, N. Site-Specific Labeling of Endogenous Proteins Using CoLDR Chemistry. *J Am Chem Soc* **2021**, *143* (48), 20095-20108.
